# A peri-tumoral TREM2-high myeloid state, myeloid continuum polarization, and white matter disconnection in long-term cognitive survivorship after diffuse glioma: a neuroimmune-connectomic cohort study

**DOI:** 10.3389/fimmu.2026.1861684

**Published:** 2026-09-30

**Authors:** Zheng Zhang, Yanyu Qi

**Affiliations:** 1 Department of Neurology, The Third People’s Hospital of Chengdu, Chengdu, Sichuan, China; 2 Department of Oncology, The Third People’s Hospital of Chengdu, Chengdu, Sichuan, China

**Keywords:** arcuate fasciculus, cognitive survivorship, connectome-based lesion-symptom mapping, glioma-associated myeloid cells, multiplex immunofluorescence, myeloid activation continuum, observational mediation analysis, prediction model

## Abstract

**Background:**

Whether a TREM2-high myeloid state and myeloid continuum polarization are associated with long-term cognitive survivorship in diffuse glioma has not been integrated with white matter connectomics and systemic neuroinflammation in a longitudinal framework.

**Methods:**

In a single-center retrospective cohort of 214 adults with WHO grade 2–4 diffuse glioma (168 IDH-mutant and 46 IDH-wildtype; median follow-up, 5.2 years), a 6-plex tyramide-amplified multiplex immunofluorescence panel was applied to 186 tumors. FlowSOM meta-clustering of 2.41 × 10^6^ myeloid cells across the tumor core, peri-tumoral white matter, and infiltrative margin generated a patient-level Glioma-Associated Myeloid Polarization Continuum Index (GAM-PCI). Connectome-based lesion-symptom mapping, longitudinal serum biomarker profiling, and observational mediation models were integrated. The tissue-connectomic mediation model included 162 complete cases, and the serum-path model included 45 patients with complete paired serum and tissue data.

**Results:**

FlowSOM resolved six marker-defined meta-clusters (bootstrap Jaccard, 0.78–0.88). C4 was TREM2-high with all other panel markers intermediate (17.1% of myeloid cells), was enriched in peri-tumoral white matter relative to tumor core (21.0% vs. 13.8%, P < 0.001), and remained associated with arcuate fasciculus (AF) disconnection after adjustment for GAM-PCI (β = 0.21 per 10-percentage-point increase, P = 0.002). CD163+CD206+ cells showed greater spatial colocalization with myelin basic protein fragments in peri-tumoral white matter (Morisita–Horn index, 0.71 [0.58–0.82]) than at the infiltrative margin (0.42 [0.31–0.55]; P < 0.001), and their mean distance to the white matter boundary was 38 ± 14 µm versus a null median of 87 µm (P < 0.001). In 45 paired serum–tissue cases, T2 serum sTREM2 was associated with the peri-tumoral C4 proportion (Spearman ρ = 0.42, P = 0.004). Long-term cognitive decline occurred in 88 of 214 patients (41.1%), and AF disconnection independently predicted impairment (adjusted odds ratio, 1.52 per 10% increase; 95% CI, 1.25–1.88). In 162 complete tissue-connectomic cases, the model-based serial indirect association through MBP loss and AF disconnection was β = −0.19 (95% CI, −0.29 to −0.10; P < 0.001). In a separate 45-patient serum-path model, the estimated indirect association through T2 sTREM2/IL-6 was β = −0.06 (95% CI, −0.11 to −0.02; P = 0.004). The AF × IDH interaction was significant (P = 0.032), whereas a difference in C4 proportion by IDH status was not detected and equivalence was not established (TOST P = 0.078).

**Conclusion:**

In this observational cohort, marker-defined peri-tumoral myeloid states, white matter disconnection, and systemic neuroimmune abnormalities were associated with long-term cognitive outcome. The mediation analysis provide model-dependent structural decompositions under stated assumptions and do not establish causal direction, cellular origin, or treatment effects.

## 1 Introduction

Diffuse gliomas are the most common primary malignant tumors of the central nervous system, with an age-adjusted incidence of 6.86 per 100,000 population and accounting for 22.9% of all primary brain tumors; glioblastoma alone represents 51.5% of malignant central nervous system tumors (1, 2). Advances in maximal safe resection, chemoradiation, and molecular stratification under the 2021 World Health Organization classification have improved median survival, particularly in isocitrate dehydrogenase (IDH)-mutant subtypes (3). Consequently, the survivorship burden has shifted: 50–80% of long-term survivors develop persistent cognitive deficits that impair return to work and quality of life (4, 5). However, the biological determinants distinguishing cognitively resilient from impaired survivors remain poorly defined, and current surgical planning relies on cortical eloquence mapping with limited consideration of subcortical network integrity. Voxel-based lesion-symptom mapping (VLSM) and connectome-based lesion-symptom mapping (CLSM) have advanced the anatomical localization of post-surgical deficits beyond the resection cavity (6). Recent studies have identified subcortical association tracts — notably the arcuate fasciculus (AF) and the inferior fronto-occipital fasciculus (IFOF) — as structural determinants of irreversible language and executive dysfunction in glioma survivors (7, 8). Yet existing CLSM analyses remain cross-sectional, use binary lesion stratification that ignores disconnection gradient, and treat white matter damage as a static anatomical endpoint rather than an active biological process shaped by the tumor microenvironment.

Concurrently, diffuse glioma has been reconceptualized as a neuroimmunological disease. Glioma-associated microglia and macrophages (GAMs) constitute 30–50% of the tumor mass and exist along a functional continuum rather than a dichotomous M1/M2 axis, with immunosuppressive CD163^+^CD206^+^ and TREM2-high disease-associated-microglia (DAM)-like states predominating at the tumor–brain interface (9–11). These myeloid populations secrete matrix metalloproteinases, reactive oxygen species, and pro-inflammatory cytokines that can injure peri-tumoral myelin and compromise axonal integrity (12). Adjuvant radiotherapy further amplifies neuroinflammation and glial activation in peri-tumoral white matter (13). Despite this convergent evidence, the tumor neuroimmune microenvironment and the structural connectomic landscape have been studied in parallel rather than as integrated determinants of cognitive survivorship, and circulating biomarkers of neuroinflammation and axonal injury have not been systematically incorporated into connectomic prediction models. To bridge this gap, we developed a neuroimmune-connectomic framework integrating structural disconnectomics, multiplex immunophenotyping of the GAM continuum, and longitudinal serum neuroimmune biomarkers (14, 15). In a retrospective longitudinal cohort of 214 adults with diffuse glioma (WHO grades 2–4; median follow-up, 5.2 years), with tissue phenotyping in 186 patients and longitudinal serum profiling in 72 patients, we tested three prespecified hypotheses: (i) AF disconnection probability is associated with 5-year cognitive impairment independently of clinical covariates; (ii) the observed GAM-PCI–language association is statistically consistent with indirect pathways involving peri-tumoral myelin loss, AF disconnection, and systemic biomarkers; and (iii) the prognostic association of the connectomic–immune signature differs by IDH mutation status.

## 2 Materials and methods

### 2.1 Study design and ethical approvals

The study was conducted in accordance with the Declaration of Helsinki and was approved by the Medical Ethics Committee of The Third People’s Hospital of Chengdu (approval number 2026-S-22). The retrospective use of clinical, neuroimaging, histopathological, and banked biospecimen data, as well as the longitudinal neuropsychological assessments administered at the preoperative baseline (T0), subacute postoperative (T1), and long-term follow-up (T2; median 5.2 years) time points, was authorized under the institutional retrospective data-use resolution 2021-R-18. This authorization explicitly covered the standardized neuropsychological battery performed at all three time points, including the 5-year follow-up assessment at T2, as part of routine long-term survivorship evaluation within the institutional glioma care pathway. Given the retrospective nature of the study and the use of de-identified data, written informed consent was waived by the ethics committee, and an opt-out notification was posted on the institutional research portal. Banked tissue and serum specimens were analyzed under the institutional biobank consent framework, which provides broad consent for secondary research use of residual diagnostic material and clinical samples. The manuscript conforms to the STROBE statement for observational studies and to the TRIPOD+AI reporting guideline for the prediction-model component.

### 2.2 Participants

Consecutive adult patients (aged 18–75 years) who underwent craniotomy for primary diffuse glioma between January 2018 and December 2020 were screened. Inclusion criteria were: (i) histopathologically confirmed World Health Organization (WHO) grade 2–4 diffuse glioma per the 2021 WHO classification; (ii) availability of high-resolution structural MRI at preoperative baseline (T0), 3–6 months postoperatively (T1), and >4 years post-surgery (T2); (iii) completion of the standardized neuropsychological battery at all three time points; and (iv) right-handedness. Exclusion criteria were prior neurosurgical intervention, pre-existing neurological or psychiatric disorders, multicentric or infratentorial tumor, severe postoperative complications, and MRI artifacts precluding reliable processing. Of 842 screened patients, 214 met all criteria (median follow-up 5.2 years; IQR 4.8–6.1). Formalin-fixed paraffin-embedded (FFPE) tumor tissue was available for 186 patients (immunohistochemistry sub-cohort); banked serum at all three time points for 72 patients (biomarker sub-cohort); and individual diffusion tensor imaging (DTI) data for 85 patients (DTI validation sub-cohort).

Representativeness of the two tissue- and serum-defined sub-cohorts relative to the primary cohort was pre-specified as a key methodological safeguard against selection bias. Demographic, clinical, molecular, treatment, and connectomic variables were systematically compared between the biomarker sub-cohort (n = 72) and the non-biomarker cohort (n = 142) using independent samples t-test, Mann–Whitney U test, or Pearson’s χ^2^/Fisher’s exact test as appropriate (**Supplementary Table S1**). The same pre-specified comparison was conducted between the immunohistochemistry sub-cohort (n = 186) and the 28 patients without available tumor tissue (**Supplementary Table S5**); reasons for tissue unavailability — insufficient residual diagnostic material (n = 15), quality degradation precluding immunostaining (n = 8), and administrative retrieval failure (n = 5) — were recorded to document a missing-completely-at-random mechanism.

### 2.3 Clinical and neuropsychological assessment

A comprehensive neuropsychological battery was administered by two trained neuropsychologists blinded to MRI, immunohistochemistry, and biomarker data, using alternate versions at each time point to minimize practice effects. The battery assessed five cognitive domains: language (Boston Naming Test, Category Verbal Fluency), verbal memory (Hopkins Verbal Learning Test–Revised immediate and delayed recall), executive function (Trail Making Test B, Stroop Color-Word Test), visuospatial attention (Trail Making Test A, Line Bisection), and processing speed (Digit Symbol Substitution Test). Raw scores were converted to age-, sex-, and education-adjusted Z-scores using normative data from a Chinese population-based healthy control sample (n = 500). Domain-specific Z-scores were computed as the mean of within-domain test Z-scores. Consistent with **Table 1**, long-term cognitive decline (LTCD) was defined as a decline of ≥1.5 SD in at least one cognitive domain at T2 relative to normative data. Global cognitive impairment, the binary outcome for the prognostic model, was defined as a composite Z-score < −1.5 SD at T2.

**Table 1:** Baseline demographics, clinical characteristics, and molecular profile of the study cohort stratified by long-term cognitive outcome.

Variable	Total cohort (N = 214)	Cognitive preserved (n = 126)	Cognitive impaired (n = 88)	P-value
Demographics				
Age at diagnosis, years, mean (SD)	44.2 (11.5)	41.5 (10.8)	48.1 (11.2)	**<0.001 a**
Sex, No. (%)				0.42 b
Male	118 (55.1)	67 (53.2)	51 (58.0)	
Female	96 (44.9)	59 (46.8)	37 (42.0)	
Education, years, median [IQR]	14 [12–16]	16 [12–18]	12 [9–15]	**<0.001 c**
KPS score, median [IQR]	90 [80–100]	90 [80–100]	80 [70–90]	**0.021 c**
Tumor characteristics				
Tumor laterality, No. (%)				**0.004 b**
Left hemisphere	112 (52.3)	58 (46.0)	54 (61.4)	
Right hemisphere	102 (47.7)	68 (54.0)	34 (38.6)	
Tumor location (lobe), No. (%)				0.15 b
Frontal	98 (45.8)	55 (43.7)	43 (48.9)	
Temporal	62 (29.0)	38 (30.2)	24 (27.3)	
Parietal/occipital	34 (15.9)	22 (17.5)	12 (13.6)	
Insular/limbic	20 (9.3)	11 (8.7)	9 (10.2)	
Tumor volume, cm³, median [IQR]	32.5 [18.2–55.4]	28.4 [15.1–48.2]	38.6 [22.5–65.1]	**0.012 c**
WHO classification and molecular profile				
WHO grade (2021), No. (%)				0.08 b
Grade 2	105 (49.1)	68 (54.0)	37 (42.0)	
Grade 3	74 (34.6)	42 (33.3)	32 (36.4)	
Grade 4 (glioblastoma)	35 (16.4)	16 (12.7)	19 (21.6)	
IDH mutation status, No. (%)				**0.035 b**
IDH-mutant	168 (78.5)	105 (83.3)	63 (71.6)	
IDH-wildtype	46 (21.5)	21 (16.7)	25 (28.4)	
1p/19q codeletion, No. (%)				0.28 b
Codeleted (oligodendroglioma)	82 (38.3)	52 (41.3)	30 (34.1)	
Non-codeleted	132 (61.7)	74 (58.7)	58 (65.9)	
MGMT methylation (when available), No. (%)	98/192 (51.0)	62/115 (53.9)	36/77 (46.8)	0.33 b
Treatment				
Extent of resection, No. (%)				0.09 b
Gross total resection (GTR)	142 (66.4)	89 (70.6)	53 (60.2)	
Subtotal resection (STR)	72 (33.6)	37 (29.4)	35 (39.8)	
Adjuvant radiotherapy, No. (%)	158 (73.8)	88 (69.8)	70 (79.5)	0.11 b
RT dose >54 Gy, No. (%)	68 (31.8)	32 (25.4)	36 (40.9)	**0.018 b**
Adjuvant temozolomide, No. (%)	185 (86.4)	110 (87.3)	75 (85.2)	0.65 b
Follow-up				
Follow-up duration, years, median [IQR]	5.2 [4.8–6.1]	5.3 [4.9–6.3]	5.1 [4.6–5.9]	0.34 c

Data are presented as mean (standard deviation), median [interquartile range], or number (percentage). Bold P-values indicate statistical significance (P < 0.05). Cognitive impaired was defined as a decline of ≥ 1.5 SD in at least one cognitive domain at 5-year follow-up relative to normative data; cognitive preserved included patients performing within the normal range. a, Independent samples t-test. b, Pearson’s χ² or Fisher’s exact test, as appropriate. c, Mann–Whitney U test. KPS, Karnofsky Performance Status; IDH, isocitrate dehydrogenase; WHO, World Health Organization; MGMT, O6-methylguanine-DNA methyltransferase; GTR, gross total resection; STR, subtotal resection; RT, radiotherapy; IQR, interquartile range; SD, standard deviation.

### 2.4 MRI acquisition, preprocessing, and lesion segmentation

MRI was performed on a 3.0-T Siemens Magnetom Prisma scanner with a 64-channel head coil. The protocol included 3D T1-weighted MPRAGE (1 mm isotropic), T2-weighted FLAIR, and for the DTI sub-cohort, single-shot echo-planar DTI (64 directions, b = 1000 s/mm^2^). T1-weighted images were registered to the MNI152 template (1 × 1 × 1 mm^3^) using the symmetric diffeomorphic registration algorithm implemented in Advanced Normalization Tools v2.3.5. Enantiomorphic normalization with cost-function masking mitigated tumor-induced deformation. Surgical cavities and residual tumor were manually segmented on T1-weighted images, cross-referenced with FLAIR to distinguish structural damage from vasogenic edema. Primary segmentation by a board-certified neuroradiologist (Reader 1) was independently verified by a senior neurosurgeon; a pre-specified stratified random sample of 20 patients was re-segmented by an independent Reader 2 for inter-rater reliability. Pre-specified acceptance criteria — mean Dice Similarity Coefficient ≥0.85, median 95th-percentile Hausdorff Distance ≤5 mm, and Intraclass Correlation Coefficient ICC(2,1) ≥0.90 with lower 95% CI bound ≥0.80 — were met across all reliability metrics.

### 2.5 Voxel- and connectome-based lesion-symptom mapping

Voxel-based lesion-symptom mapping (VLSM) was conducted in NiiStat, restricted to voxels damaged in ≥5% of patients. Voxel-wise comparisons used the non-parametric Brunner–Munzel test with total lesion volume as a covariate, and permutation-based false discovery rate (FDR) correction (5,000 permutations, q < 0.05). To quantify white matter disconnection beyond visible lesion boundaries, the Disconnectome approach implemented in the BCBtoolkit was applied (16). Each patient’s normalized lesion mask was embedded into the Human Connectome Project 842-subject normative streamline atlas to generate voxel-wise probabilistic disconnection maps (range 0–1). Tract-specific disconnection probabilities were extracted for the arcuate fasciculus (AF), inferior fronto-occipital fasciculus (IFOF), uncinate fasciculus (UF), superior longitudinal fasciculus (SLF I–III), anterior thalamic radiation (ATR), and frontal aslant tract (FAT). To address potential optimism bias, all primary regression analysis used tract disconnection probability as a continuous predictor (per 10% increment); binary stratification (tract intact vs. disconnected, threshold 0.50) was retained only for trajectory visualization.

In the 85-patient DTI sub-cohort, patient-specific probabilistic tractography was performed using MRtrix3 v3.0.4 (17), with anatomically constrained tractography (10 million streamlines) and SIFT2 filtering. Tract-specific fractional anisotropy values were correlated with CLSM-derived disconnection probabilities using Spearman rank correlation. The AF and UF correlation analyses are shown in **Figures 1d, e**, and the prespecified individual-patient Bland–Altman agreement analysis are shown in **Supplementary Figure S1**. Together, these analyses provided the methodological validation for use of the normative Disconnectome approach in clinical workflows lacking routine individual diffusion acquisition.

**Figure 1:**
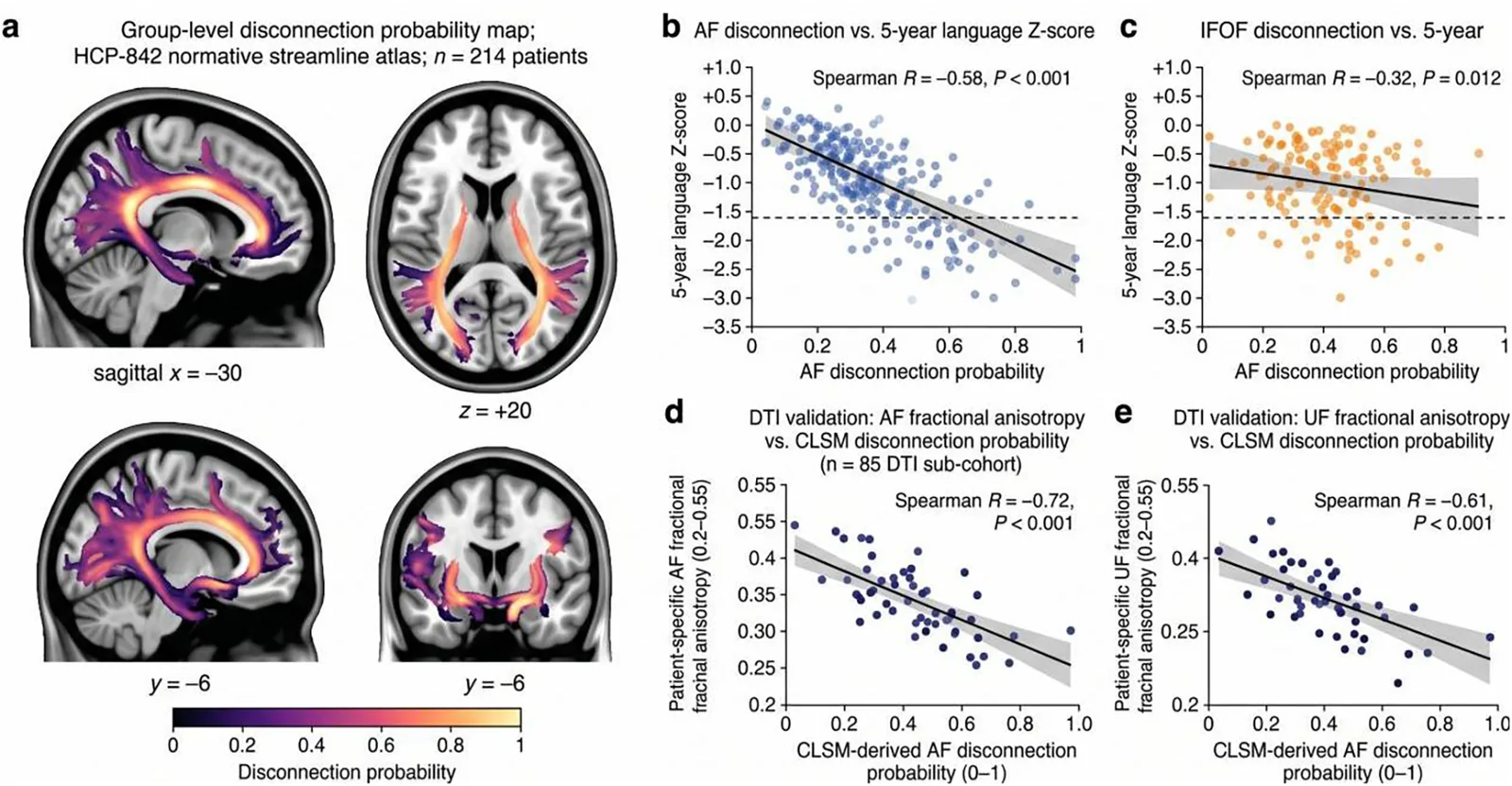
Connectome-based lesion-symptom mapping and patient-specific DTI validation. **(a)** Group-level disconnection probability map generated by projecting lesion masks from all 214 patients onto the HCP-842 normative streamline atlas. The color scale ranges from 0 to 1. **(b)** AF disconnection probability versus the 5-year Language Z-score (Spearman ρ = −0.58, P < 0.001; n = 214). **(c)** IFOF disconnection probability versus the 5-year Language Z-score (ρ = −0.32, P = 0.012; n = 214). Solid lines show fitted trends and shaded bands show 95% confidence intervals. **(d)** Patient-specific AF fractional anisotropy versus CLSM-derived AF disconnection probability in the DTI sub-cohort (ρ = −0.72, P < 0.001; n = 85). **(e)** Patient-specific UF fractional anisotropy versus CLSM-derived UF disconnection probability (ρ = −0.61, P < 0.001; n = 85). AF, arcuate fasciculus; CLSM, connectome-based lesion-symptom mapping; DTI, diffusion tensor imaging; HCP, Human Connectome Project; IFOF, inferior fronto-occipital fasciculus; UF, uncinate fasciculus.

### 2.6 Multiplex immunofluorescence phenotyping of the glioma-associated myeloid continuum

Because contemporary single-cell studies have established that glioma-associated microglia and macrophages (GAMs) exist along a functional continuum rather than a dichotomous M1/M2 axis, a 6-plex tyramide signal amplification-based multiplex immunofluorescence (mIF) assay was implemented on the Opal 7-color system (Akoya Biosciences). The panel simultaneously resolved pan-myeloid identity (CD68), pan-microglial identity (Iba1), alternative/immunosuppressive polarization (CD163, CD206), pro-inflammatory polarization (iNOS), and the TREM2 axis associated with the disease-associated-microglia (DAM) program within a single section. The panel was designed to resolve myeloid activation state and contains no ontogeny-informative marker; resident microglia and monocyte-derived macrophages are therefore not separable at the single-cell level in this dataset, and no meta-cluster designation should be read as a lineage assignment. Four-µm FFPE sections adjacent to those used for conventional immunohistochemistry were dewaxed, subjected to heat-induced epitope retrieval (Tris-EDTA pH 9.0, 97 °C, 20 min) on a Leica Bond RX autostainer, and sequentially stained in the pilot-optimized order: CD68 → Iba1 → CD163 → CD206 → iNOS → TREM2 → DAPI. Whole-slide multispectral images were acquired on a Vectra Polaris/PhenoImager HT at 20× magnification. Nuclear segmentation was performed with StarDist v0.8.3 integrated into QuPath v0.5.0 (18); approximately 2.4 × 10^6^ CD68- or Iba1-positive cells were retained across 186 patients.

The Opal 7-color system accommodates six antibody channels plus a DAPI counterstain, all of which were occupied by the panel above. P2RY12 was therefore assessed separately by chromogenic single-plex immunohistochemistry (rabbit polyclonal anti-P2RY12, Atlas Antibodies Cat# HPA014518, RRID: AB_10602264, 1:200) on serial sections cut 4 µm adjacent to the multiplex section, on a Leica Bond-III autostainer, in all 186 cases. Adjacent-section staining was performed without image registration and does not establish cell-level correspondence with mIF-defined clusters; it is reported only as regional evidence. P2RY12-immunoreactive cells were quantified per anatomical zone both as absolute density (cells per high-power field) and as a ratio to independently measured Iba1-positive cell density within the same anatomical compartment. Because absolute density is confounded by total myeloid cellularity, which differs approximately two-fold between zones, the ratio is the primary regional metric. Patient-level Spearman rank correlations were computed between the P2RY12^+^/Iba1^+^ ratio and the per-patient proportion of each of the six meta-clusters; because the six proportions sum to unity within each patient, the covariances of any external variable with them are constrained to sum to zero, and the weighted sum Σ(R × SD) is reported as a consistency check.

Single-cell marker intensities were arcsinh-transformed (cofactor 150) and z-scored across all 2.41 × 10^6^ cells; meta-clustering was performed on these cell-level z-scores, in the order stated. Cluster medians reported in **Figure 2b** and **Supplementary Table S12** are medians of cell-level z-scores, which is why the column means of the cluster-median matrix are not identically zero (for example, Iba1 mean +0.58, reflecting broad myeloid expression). A self-organising map was trained on a 4 × 5 grid (20 nodes) using FlowSOM, and node medians were meta-clustered by consensus clustering over k = 4–12 with 1,000 resamples at 80% subsampling. The previous description of meta-clustering “to k = 8” was a transcription error; no eight-cluster intermediate step was performed.

**Figure 2:**
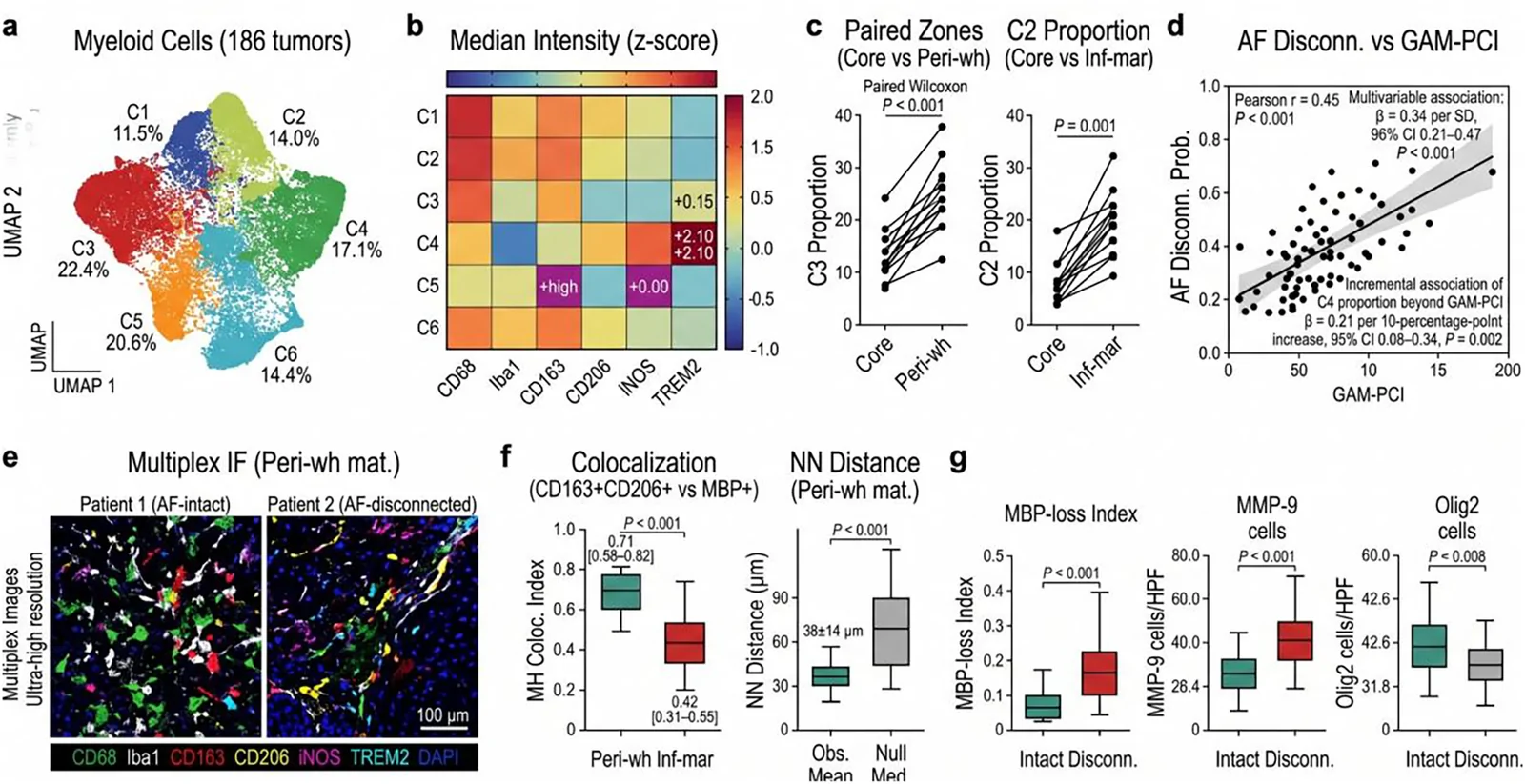
Multiplex immunofluorescence defines six marker-based glioma-associated myeloid states at the tumor–brain interface and demonstrates patient-level associations with myelin injury and arcuate fasciculus disconnection. **(a)** UMAP visualization of 2.41 × 10^6^ CD68+ or Iba1+ cells from 186 tumors. UMAP was used for visualization only. Overall cluster proportions were C1, 11.5%; C2, 14.0%; C3, 22.4%; C4, 17.1%; C5, 20.6%; and C6, 14.4%. **(b)** Cluster-median z-score heat map for CD68, Iba1, CD163, CD206, iNOS, and TREM2. C4 had the highest TREM2 z-score (+2.10); the next-highest cluster was +0.15. **(c)** Patient-level paired zone comparisons: C3, tumor core 17.6% versus peri-tumoral white matter 26.9% (paired Wilcoxon P < 0.001); C2, tumor core 11.2% versus infiltrative margin 19.3% (P = 0.001). **(d)** GAM-PCI versus AF disconnection probability (n = 186; Pearson r = 0.45, P < 0.001). The adjusted GAM-PCI coefficient was β = 0.34 per SD (95% CI, 0.21–0.47; P < 0.001), and C4 remained associated beyond GAM-PCI (β = 0.21 per 10-percentage-point increase; 95% CI, 0.08–0.34; P = 0.002). **(e)** Representative peri-tumoral mIF composites from an AF-intact and an AF-disconnected patient; channels are CD68, Iba1, CD163, CD206, iNOS, TREM2, and DAPI; scale bar, 100 µm. **(f)** Morisita–Horn colocalization of CD163+CD206+ cells with MBP+ fragments in peri-tumoral white matter versus infiltrative margin (0.71 [0.58–0.82] vs. 0.42 [0.31–0.55], P < 0.001) and nearest-neighbor distance from CD163+CD206+ cells to the white matter boundary (38 ± 14 µm vs. null median 87 µm, P < 0.001). **(g)** MBP-loss index in 162 evaluable cases (0.12 [0.05–0.22] vs. 0.34 [0.22–0.48], P < 0.001), MMP-9+ density (26.4 [SD 10.2] vs. 48.2 [SD 15.6] cells/HPF, P < 0.001), and Olig2+ density (42.6 [SD 13.8] vs. 31.8 [SD 11.2] cells/HPF, P = 0.008), shown as AF-intact versus AF-disconnected. Cluster names describe marker profiles and do not imply ontogeny or formal M1/M2 identity. AF, arcuate fasciculus; DAPI, 4′,6-diamidino-2-phenylindole; GAM-PCI, glioma-associated myeloid polarization continuum index; HPF, high-power field; MBP, myelin basic protein; mIF, multiplex immunofluorescence; UMAP, Uniform Manifold Approximation and Projection.

k was selected on three prospective criteria applied in order: minimisation of the proportion of ambiguous clustering (PAC); the relative change in the area under the consensus cumulative distribution function (ΔAUC = 0.35 at k = 5, 0.12 at k = 6 and 0.04 at k = 7, so that the seventh cluster adds less than the 0.05 threshold and k = 6 is the elbow); and phenotypic non-redundancy, defined as a minimum pairwise Euclidean distance greater than 1.2 in cell-level z-score space, which became binding at k = 7 where two clusters lay 0.98 apart. In a *post-hoc* check, running the same pipeline on a standard 10 × 10 grid (100 nodes) and meta-clustering to k = 6 returned the same six-state partition (adjusted Rand index 0.94), confirming that the 20-node grid was sufficient. Two-dimensional projection by UMAP (n_neighbors = 30, min_dist = 0.3) was used for visualisation only and played no part in defining clusters.

Meta-clusters are named by their marker profile using a pre-specified three-tier rule applied mechanically to the cluster-median z-score matrix: high if z ≥ +1.0, low if z ≤ −1.0, intermediate otherwise. Only markers reaching the high or low tier appear in a cluster name; intermediate markers are either omitted or stated explicitly as “int”. Because the panel resolves activation state and not ontogeny, cluster names carry no implication of lineage; interpretive designations are given separately in **Table 2** and identified as such. A per-patient GAM polarization continuum index (GAM-PCI) was defined as the first principal component of the arcsine-transformed meta-cluster proportion vector, oriented so that higher values indicate greater M2/TREM2-skewed composition. GAM-PCI replaced the conventional CD163^+^/CD68^+^ ratio as the primary immune exposure variable in all multivariable analysis. Five 1 mm × 1 mm representative regions of interest per anatomical zone (tumor core, peri-tumoral white matter within 5 mm of the tumor–brain interface, and infiltrative margin) were selected by a board-certified neuropathologist blinded to all clinical data. Morisita–Horn spatial colocalization indices between myeloid phenotypes and MBP-positive myelin fragments were computed using the spatstat R package v3.0-7. T-cell densities (CD3, CD8) were quantified on serial sections by conventional chromogenic single-plex immunohistochemistry on a Leica Bond-III autostainer, with whole-slide images analyzed in QuPath v0.5.0.

**Table 2:** Tumor immune microenvironment profile across anatomical zones and stratified by Arcuate Fasciculus disconnection status, including multiplex immunofluorescence-derived continuum markers.

Parameter	Tumor core AF-Int (n=120)	Tumor core AF-Dis (n=66)	Peri-WM AF-Int	Peri-WM AF-Dis	Infiltrative margin (all)	P †
Marker density, cells/HPF, mean (SD)						
CD68^+^	43.2 (18.1)	50.5 (21.4)	58.4 (20.1)	74.5 (23.8)	82.4 (28.6)	<0.001
Iba1^+^	36.5 (15.2)	41.3 (18.1)	64.8 (22.4)	82.8 (27.5)	58.6 (24.2)	<0.001
CD163^+^	26.1 (11.5)	32.6 (14.2)	35.8 (15.2)	54.8 (20.4)	52.1 (22.4)	<0.001
CD206^+^	18.4 (8.2)	24.8 (10.1)	28.6 (11.4)	46.2 (15.8)	35.2 (13.6)	<0.001
iNOS^+^	21.5 (9.4)	24.2 (10.8)	18.6 (8.5)	20.4 (9.1)	35.8 (14.2)	<0.001
TREM2^+^	15.8 (7.2)	19.4 (8.8)	22.5 (9.6)	38.6 (13.4)	28.4 (11.2)	<0.001
CD3^+^	17.8 (7.8)	19.8 (9.1)	21.2 (9.8)	24.6 (11.5)	25.8 (12.1)	0.12
CD8^+^	6.5 (3.8)	7.4 (4.8)	7.8 (4.9)	9.0 (6.2)	10.4 (6.8)	0.18
Composite polarization indices						
GAM-PCI, median [IQR] a	−0.38 [−0.75, 0.02]	−0.12 [−0.48, 0.35]	−0.15 [−0.52, 0.28]	+0.54 [0.18, 0.92]	+0.08 [−0.30, 0.45]	<0.001
M2/M1 ratio (CD163^+^/CD68^+^)	0.40 (0.11)	0.45 (0.13)	0.61 (0.14)	0.74 (0.13)	0.55 (0.14)	<0.001
FlowSOM C3 (CD163hi CD206hi), %	16.4 (6.2)	19.8 (7.4)	24.5 (8.8)	31.2 (10.4)	22.8 (8.5)	<0.001
FlowSOM C4 (TREM2hi), %	12.8 (5.4)	15.6 (6.8)	18.4 (7.6)	25.8 (9.2)	16.4 (6.8)	<0.001
Myelin injury and matrix remodeling						
MBP-loss index b	—	—	0.12 [0.05, 0.22]	0.34 [0.22, 0.48]	—	<0.001
Peri-tumoral MMP-9^+^, cells/HPF	—	—	26.4 (10.2)	48.2 (15.6)	38.5 (13.4)	<0.001
Olig2^+^, cells/HPF	46.2 (14.8)	38.6 (12.4)	42.6 (13.8)	31.8 (11.2)	28.4 (10.8)	0.008

Data are presented as mean (SD) unless otherwise indicated. Immunohistochemistry and multiplex immunofluorescence were performed on FFPE tissue from 186 of 214 patients. AF-intact and AF-disconnected tissue subgroups comprised 120 and 66 patients, respectively. GAM-PCI is the first principal component of the arcsine-transformed FlowSOM meta-cluster proportion vector and captured 47.2% of variance. The MBP-loss index was calculated in 162 cases with evaluable contralateral tissue. Meta-cluster names are based solely on the prespecified marker-intensity rule (high z ≥ +1.0; low z ≤ −1.0; intermediate otherwise): C1, CD68lo CD163lo CD206lo TREM2lo; C2, CD68hi iNOShi CD163lo CD206lo; C3, CD163hi CD206hi; C4, TREM2hi with other markers intermediate; C5, CD163hi iNOShi; and C6, Iba1hi CD68lo. These names describe activation-marker profiles and do not assign ontogeny or formal M1/M2 identity. Zone-level proportions are sample-size-weighted means of per-patient values. The primary P value compares AF-intact and AF-disconnected patients in peri-tumoral white matter. AF, arcuate fasciculus; DAM, disease-associated microglia; FFPE, formalin-fixed paraffin-embedded; GAM-PCI, glioma-associated myeloid polarization continuum index; HPF, high-power field; MBP, myelin basic protein; WM, white matter.

#### 2.6.1 Cluster validation and stability.

Internal validity of the meta-clustering solution was assessed across k = 4–12 by PAC, the relative change in consensus-CDF area, mean silhouette width, and the Calinski–Harabasz and Davies–Bouldin indices (**Supplementary Table S10**). Cluster-wise stability was quantified by non-parametric bootstrap using fpc::clusterboot with 500 patient-level resamples, the full pipeline (self-organising map training and meta-clustering) being re-run within each resample and clusters matched to the reference partition by maximum Jaccard overlap; a mean Jaccard coefficient of at least 0.75 was taken to indicate a stable cluster. Reproducibility of the partition was assessed by re-running the pipeline under 10 independent random seeds and computing the pairwise adjusted Rand index. Robustness of the substantive findings to the clustering solution was pre-specified as the decisive criterion: the peri-tumoral versus tumour-core enrichment of the TREM2-high population, and the association of its per-patient proportion with arcuate fasciculus disconnection probability adjusted for GAM-PCI, were re-estimated under every solution in the range k = 5–9, under 10 random seeds, and under Leiden clustering of the same cell-level z-scored matrix. In each alternative solution the TREM2-high population was re-identified without reference to the original labels, as the meta-cluster with the highest median TREM2 z-score, and per-patient proportions and GAM-PCI were recomputed within that partition (**Supplementary Table S11**).

#### 2.6.2 Cell yield, quality control and low-yield sensitivity analysis.

Five 1 mm × 1 mm regions of interest were acquired per anatomical zone and three zones were sampled per patient, giving a nominal sampling frame of 15 mm² per patient. Segmented objects were retained as myeloid if CD68- or Iba1-positive after per-slide thresholding. The number of retained cells was recorded for every patient–zone unit and for every zone × meta-cluster combination (**Supplementary Table S13**). Meta-cluster proportions were computed within each patient–zone unit and then summarised across patients as sample-size-weighted means of per-patient proportions, so that patients contributing more cells do not dominate zone-level estimates; zone contrasts were tested by paired Wilcoxon signed-rank test, each patient contributing both zones. Two low-yield thresholds were pre-specified at the patient–zone level (200 and 500 retained cells); because no unit fell below either, a third analysis targeted at the level at which proportional estimates are least stable was performed, excluding patient–zone units in which any single meta-cluster contained fewer than 50 cells (**Supplementary Table S14**).

To examine anatomical specificity and compare measured myeloid and adaptive-immune signals, a prespecified patient-level Spearman correlation matrix was constructed between six peri-tumoral immune measures (CD68+ density, CD163+ density, Iba1+ density, CD3+ density, CD8+ density, and the legacy CD163+/CD68+ ratio) and disconnection probabilities across five association fasciculi (AF, IFOF, UF, SLF, and FAT). The matrix and the AF-specific effect summary are presented in **Supplementary Figure S2**. In the biomarker sub-cohort, Spearman rank correlations were calculated between six log10-transformed T2 serum biomarkers (NfL, GFAP, S100B, IL-6, TNF-α, and IL-1β) and seven prespecified connectomic or cognitive variables: AF, IFOF, and UF disconnection probabilities and the Language, Memory, Executive Function, and Global Composite Z-scores. This produced 42 prespecified pairwise analyses. Each correlation was estimated using pairwise-complete observations without imputation. The signed Spearman coefficient, pairwise-complete sample size, and numerical two-sided P value were obtained from the same analysis output and are reported in **Supplementary Table S7**. Nominal P values are reported for transparency and are interpreted as exploratory because of multiple correlated comparisons.

To verify that the protein-level TREM2-high meta-cluster corresponds to a transcriptionally coherent DAM-like program, mIF cluster annotations were compared with two publicly available glioma single-cell RNA-sequencing resources: the Abdelfattah et al. atlas (GSE182109; 201,986 cells from 18 patients) (19) and the GBmap harmonized integrated atlas, which integrates 26 single-cell datasets comprising more than 1.1 million cells from 240 patients (20). Raw count matrices were processed in R v4.2.0 with Seurat v5.0.1 (21); myeloid-lineage cells were identified by canonical markers (PTPRC, CD68, ITGAM, AIF1, CX3CR1, P2RY12) and re-clustered using SCTransform and Leiden clustering (resolution 0.6). A DAM-like signature score was computed per cell using UCell v2.4, based on the canonical Keren-Shaul/Krasemann program (TREM2, APOE, LPL, SPP1, CST7, ITGAX, AXL, CD9, LILRB4). Correspondence between the mIF and scRNA-seq populations was evaluated on proportion, panel-gene expression, and spatial niche enrichment (tumor core vs. peri-tumoral) using the GBmap 10× Visium subset. Because the comparison is between a protein-defined cluster in this cohort and a transcriptionally defined population in independent cohorts, it constitutes in silico triangulation across datasets rather than molecular validation in our own material.

### 2.7 Tissue myelin injury and matrix remodeling markers

Serial FFPE sections were stained for myelin basic protein (MBP; Abcam ab218011, 1:1000), matrix metalloproteinase-9 (MMP-9; Abcam ab76003, 1:200), and Olig2 (Millipore AB9610, 1:500) on a Leica Bond-III autostainer with appropriate positive and negative controls. Whole-slide images were acquired on an Aperio AT2 scanner at 40× magnification and analyzed in QuPath. Peri-tumoral MBP optical density was quantified as the mean DAB-positive fraction within the peri-tumoral white matter region of interest, normalized to a contralateral normal-appearing white matter reference on the same section. A peri-tumoral MBP-loss index was defined as 1 − (peri-tumoral MBP-positive fraction/contralateral MBP-positive fraction). MMP-9-positive cell density was quantified per high-power field; Olig2-positive cell density provided a denominator for oligodendrocyte-normalized myelin metrics.

### 2.8 Serum neuroimmune biomarker panel

Peripheral venous blood (5 mL) had been collected at each time point in serum separator tubes (BD Vacutainer SST II Advance), processed within 2 h by centrifugation (2000 × g, 10 min, 4 °C), aliquoted, and stored at −80 °C. Freeze–thaw cycles were limited to one prior to measurement. Samples from all three time points per patient were analyzed on the same plate to minimize within-subject drift; laboratory staff were blinded to cognitive and imaging data. The core panel comprised neurofilament light chain (NfL) and glial fibrillary acidic protein (GFAP), measured on the Simoa HD-X analyzer using the NF-Light Advantage PLUS and GFAP Advantage PLUS assays (Quanterix); S100B, measured using the Roche Elecsys electrochemiluminescence immunoassay; and interleukin-6 (IL-6), tumor necrosis factor-alpha (TNF-α), and interleukin-1β (IL-1β), measured using the Bio-Rad Bio-Plex Pro Human Cytokine 3-plex panel on the Luminex xMAP platform. The extended panel included matrix metalloproteinase-9 (MMP-9), active TGF-β1, IL-10, soluble TREM2 (sTREM2), and CHI3L1/YKL-40. Intra- and inter-assay coefficients of variation were <10% and <15%, respectively, for all analytes. Values below the lower limit of quantification were imputed as LLOQ/√2; concentrations were log_10_-transformed prior to statistical modeling.

A pre-specified biomarker analysis framework was designed to evaluate both longitudinal trajectories and cross-sectional correlations with downstream outcomes. For each analyte, log-transformed concentrations at T0, T1, and T2 were compared between AF-intact and AF-disconnected subgroups using linear mixed-effects models with main effects of Time and Group and a Time × Group interaction term. For pro-inflammatory cytokines with non-significant Time × Group interactions (TNF-α, IL-1β), the detailed trajectory and cross-sectional analyses are presented in **Supplementary Figure S3** (panels a–b: longitudinal trajectories with main-effect and interaction P-values; panels c–d: T2 cytokine concentrations against 5-year Language Z-score). A comprehensive Spearman correlation matrix between all six T2 core serum biomarkers (NfL, GFAP, S100B, IL-6, TNF-α, IL-1β) and disconnection probabilities across three association tracts (AF, IFOF, UF) plus four cognitive outcomes (Language, Memory, Executive Function, and Global Composite Z-scores) was pre-specified as the primary biomarker–outcome discovery analysis; the full matrix is provided in **Supplementary Table S7**.

### 2.9 IDH mutation status and molecular stratification

IDH1/IDH2 mutation status was determined by immunohistochemistry using the anti-IDH1 R132H monoclonal antibody (clone H09, Dianova); immunohistochemistry-negative cases were confirmed by Sanger sequencing of IDH1 codon 132 and IDH2 codon 172. The cohort comprised 168 IDH-mutant and 46 IDH-wildtype patients. MGMT promoter methylation, 1p/19q codeletion (by fluorescence *in situ* hybridization), and ATRX status were retrieved from the institutional pathology database for 192 patients (89.7%). Pre-specified IDH-stratified analysis included re-estimation of the multivariable prediction model within each IDH subgroup, a formal multiplicative interaction test (AF disconnection × IDH status) in the pooled cohort, comparison of GAM continuum phenotypes and GAM-PCI distributions between IDH subgroups, stratified sequential mediation analysis, and a pre-specified three-way Education × AF disconnection × time interaction test within each IDH subgroup for the language domain.

### 2.10 Statistical analysis

All statistical analyses were performed in R v4.2.0 and MATLAB R2023a. Two-sided P-values < 0.05 were considered statistically significant. The pre-specified primary endpoint was long-term global cognitive impairment at T2 (composite Z-score < −1.5 SD). The primary hypothesis was that AF disconnection probability predicts this outcome in a multivariable logistic regression adjusting for age, sex, education, tumor volume, IDH status, and adjuvant radiotherapy dose.

Longitudinal trajectory modeling. Linear mixed-effects models (LMMs) were fitted with the lme4 R package v1.1-35, including Time (T0, T1, T2) and tract disconnection status as fixed effects and subject as a random effect with random intercept and random slope for time. The Time × Lesion Status interaction tested whether recovery trajectories differed by white matter tract integrity. For the five cognitive domains, the main effect of Time and the Time × Group interaction were FDR-corrected by the Benjamini–Hochberg method at q < 0.05. Covariates adjusted were age, sex, education, tumor volume, and adjuvant therapy type. The full LMM output for all five cognitive domains — fixed-effect coefficients, P-values, FDR-corrected q-values, effect sizes (Cohen’s d for T0→T1 decline and T1→T2 recovery within intact and disconnected subgroups), random-effects structure, and marginal/conditional R^2^ — is presented in **Supplementary Table S6**.

Threshold sensitivity framework. Because any Youden-optimal AF threshold derived on the same cohort used for outcome modeling is subject to optimism bias, a three-layered sensitivity framework was pre-specified: (i) AF disconnection probability as a continuous predictor (primary); (ii) binary stratification at six candidate thresholds (0.30, 0.40, 0.50, 0.60, 0.70, and 0.80); and (iii) restricted cubic spline modeling with four knots at the 5th, 35th, 65th, and 95th percentiles, with a likelihood ratio test comparing spline vs. linear-term models. Operating characteristics (sensitivity, specificity, positive and negative predictive value, Youden Index, Time × Lesion interaction coefficient, and binary-model AUC) for each candidate threshold are presented in **Supplementary Table S8**; the Youden-optimal threshold of ≥0.50 was selected on the basis of the receiver operating characteristic curve for long-term cognitive decline (**Supplementary Figure S6**).

Observational sequential mediation analysis. A tissue-connectomic complete-case model included 162 patients with available GAM-PCI, peri-tumoral MBP-loss index, AF disconnection probability, 5-year Language Z-score, and prespecified covariates. GAM-PCI was specified as the exposure, MBP-loss index as mediator 1, AF disconnection probability as mediator 2, and the 5-year Language Z-score as the outcome. Model-based indirect associations were estimated with 2,000 bootstrap resamples using the mediation framework. A separate serum-path structural equation model was fitted in 45 patients with complete GAM-PCI, T2 sTREM2, T2 IL-6, Language Z-score, and covariate data. Sensitivity to residual mediator–outcome confounding was assessed by the Imai ρ method, with a prespecified robustness threshold of ρ = 0.20. All models adjusted for age, sex, education, tumor volume, IDH status, and radiotherapy dose.

Identification assumptions. Temporal ordering was not empirically established. GAM-PCI and MBP-loss were measured in surgical tissue, and AF disconnection was derived from the postoperative lesion mask; therefore, the ordering GAM-PCI → MBP loss → AF disconnection was specified on biological grounds rather than directly observed. In the serum-path model, T2 sTREM2, T2 IL-6, and the 5-year Language Z-score were measured at the same long-term follow-up interval, so reverse ordering and common-cause explanations cannot be excluded. Adjustment for extent and location of resection does not restore temporal ordering or eliminate exposure-induced mediator–outcome confounding. The reported estimates are therefore model-based structural decompositions under stated assumptions rather than identified causal effects.

Prognostic models. A multivariable logistic regression model was constructed for the primary endpoint. Variables with P < 0.10 in univariable analysis were entered using backward stepwise selection. Multicollinearity was assessed by variance inflation factors (all retained predictors VIF < 2.5). Model discrimination was evaluated by the area under the receiver operating characteristic curve with 10-fold cross-validation repeated 100 times; internal validation used 1,000 bootstrap resamples to estimate the optimism-corrected C-index. Calibration was assessed by the Hosmer–Lemeshow goodness-of-fit test and calibration plots. Clinical utility was assessed by decision curve analysis across the threshold range 15–85%. The nomogram was built with the rms R package v6.7-1. An extended nomogram incorporating T2 serum NfL and IL-6 was developed in the 72-patient biomarker sub-cohort (events-per-variable ratio ≈4.3) and was designated *a priori* as exploratory and hypothesis-generating. Incremental predictive value of biomarker addition was evaluated using nested model comparison with AUC change, DeLong’s test for paired ROC curves, net reclassification improvement (NRI), integrated discrimination improvement (IDI), and Akaike Information Criterion (**Supplementary Table S3**). Bootstrap internal validation statistics (optimism, optimism-corrected C-index, 10-fold cross-validated AUC, calibration slope, Brier score) were computed for both the base model (full cohort, N = 214) and within each IDH subgroup (n = 168 IDH-mutant; n = 46 IDH-wildtype), and are presented in **Supplementary Table S4**. The structure of the extended nomogram, calibration curve comparison, and decision curve analysis between base and extended models are presented in **Supplementary Figure S5** (panels a–c, respectively). Multivariable linear regression with the 5-year cognitive domain Z-scores as dependent variables was used to quantify the independent contribution of each serum biomarker after adjustment for clinical and connectomic covariates; the full regression output is provided in **Supplementary Table S9**.

Missing data, survivor selection, and confounding. Multiple imputation by chained equations used 40 imputations and 20 iterations. Survivor selection was evaluated using stabilized inverse-probability-of-inclusion weights. The probability model was estimated in a 466-patient baseline-eligible source population comprising the 214 patients alive and observed in the long-term analytic cohort and 252 patients who died or were not observed before 3 years. The binary model outcome was inclusion in the long-term analytic cohort, and predictors were age, IDH status, WHO grade, and extent of resection. Weights were truncated by percentile-based capping, not by deleting observations. In the primary analysis, values below the 1st percentile and above the 99th percentile were set to the corresponding percentile cut points; the sensitivity analysis used 5th/95th-percentile truncation. No trimming was performed. **Supplementary Figure S7** displays the raw and truncated weight distributions, the number of capped observations, effective sample-size annotations, and standardized mean-difference diagnostics.

Hierarchical hypothesis testing and power. A pre-specified hierarchical testing strategy controlled family-wise error: (i) primary gatekeeper — AF disconnection probability predicts the primary endpoint; (ii) secondary Hochberg-corrected hypotheses (GAM-PCI correlates with AF disconnection; peri-tumoral MBP-loss index mediates the GAM-PCI → AF disconnection → language pathway; T2 sTREM2 and CHI3L1 are elevated in AF-disconnected patients); (iii) exploratory analysis, including the Education × AF × IDH three-way interaction (visualization presented in **Supplementary Figure S4**) and the biomarker-augmented nomogram. The Benjamini–Hochberg FDR correction at q = 0.05 was additionally applied across main-text P-values. With 88 events and six candidate predictors (events-per-variable ratio 14.7), *post-hoc* power analysis using pwrss v0.3.1 indicated >95% power to detect an AF disconnection odds ratio of 1.40 per 10% increment at α = 0.05.

### 2.11 Data and code availability

Analysis code, the data dictionary, and non-identifying aggregate source data supporting the tables and figures will be deposited in Zenodo at https://doi.org/10.5281/zenodo.22243158. Individual-level clinical, cognitive, biomarker, imaging-derived, and multiplex-immunofluorescence data are not publicly available because the retrospective ethics and data-use approvals do not authorize unrestricted public dissemination and residual re-identification risk remains. Requests for a de-identified individual-level dataset or whole-slide images should be directed to the corresponding author and will be considered subject to institutional ethics approval and a data-use agreement. Raw clinical records and raw MRI data will not be shared publicly.

## 3 Results

### 3.1 Cohort characteristics and univariable determinants of long-term cognitive outcome

Of 842 patients screened between January 2018 and December 2020, 214 met all eligibility criteria and constituted the primary analysis cohort (**Figure 3A**, CONSORT flow diagram). The lesion overlap heatmap confirmed adequate anatomical coverage of the left perisylvian and paralimbic networks (**Figure 3B**). Median follow-up was 5.2 years (IQR 4.8–6.1). At T2, 88 of 214 patients (41.1%) met the pre-specified criterion for long-term cognitive decline (LTCD; **Table 1**). LTCD patients were older than cognitively preserved patients (mean 48.1 vs. 41.5 years, P < 0.001), had fewer years of education (median 12 vs. 16, P < 0.001), more frequently had left-hemispheric tumors (61.4% vs. 46.0%, P = 0.004), larger preoperative tumor volumes (median 38.6 vs. 28.4 cm^3^, P = 0.012), were more frequently IDH-wildtype (28.4% vs. 16.7%, P = 0.035), and more frequently received adjuvant radiotherapy >54 Gy (40.9% vs. 25.4%, P = 0.018). Extent of resection did not differ between groups (gross total resection 60.2% vs. 70.6%, P = 0.09).

**Figure 3:**
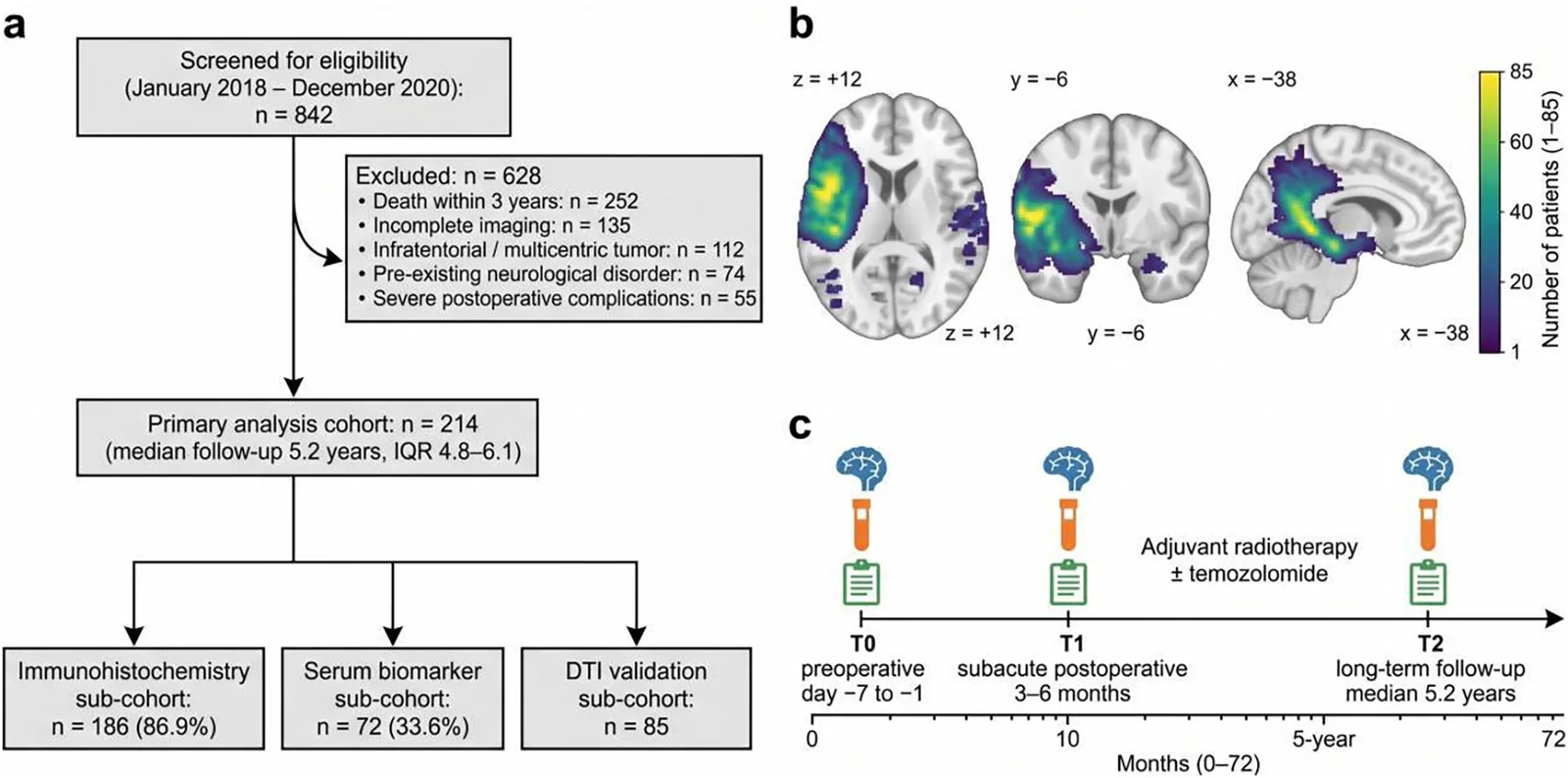
Study flow diagram, lesion overlap heatmap, and longitudinal assessment timeline. **(a)** Study flow diagram. Of 842 patients screened between January 2018 and December 2020, 628 were excluded: death within 3 years (n = 252), incomplete imaging (n = 135), infratentorial or multicentric tumor (n = 112), pre-existing neurological disorder (n = 74), and severe postoperative complications (n = 55). The final long-term analysis cohort comprised 214 patients (median follow-up 5.2 years, IQR 4.8–6.1), including tissue immunohistochemistry/mIF data for 186, longitudinal serum data for 72, and individual DTI data for 85. **(b)** Lesion-overlap heatmap from normalized lesion masks of the 214 patients in MNI152 space. The color scale represents the number of patients with lesion involvement at each voxel (range 1–85). **(c)** Assessment timeline showing T0 preoperative evaluation (day −7 to −1), T1 subacute postoperative evaluation at 3–6 months, adjuvant radiotherapy with or without temozolomide, and T2 long-term follow-up at a median of 5.2 years. DTI, diffusion tensor imaging; IQR, interquartile range; mIF, multiplex immunofluorescence; MNI, Montreal Neurological Institute.

The biomarker sub-cohort (n = 72) and the immunohistochemistry sub-cohort (n = 186) were both representative of the primary cohort on all key demographic, clinical, molecular, treatment, and connectomic variables (all P > 0.05; **Supplementary Tables S1**, **S5**). The biomarker sub-cohort showed 41.7% LTCD vs. 40.8% in the non-biomarker cohort (P = 0.91) and matched median follow-up (5.3 vs. 5.2 years, P = 0.65; **Supplementary Table S1**, Cognitive Outcome rows). Among 28 patients without available tissue for immunohistochemistry, the numerical trend toward lower AF disconnection probability (median 0.22 vs. 0.38 in tissue-available patients, P = 0.08; **Supplementary Table S5**, Connectome Metrics rows) did not reach statistical significance; tissue unavailability was attributable to insufficient residual diagnostic material (n = 15), quality degradation precluding immunostaining (n = 8), or administrative retrieval failure (n = 5). Compared with 628 excluded patients, the included cohort was representative on age, sex, tumor laterality, and IDH mutation frequency (all standardized mean differences < 0.10), with an expected over-representation of lower-grade tumors associated principally by the 252 patients excluded due to death before 3 years.

### 3.2 Inter-rater and intra-rater segmentation reliability

In the pre-specified stratified random sample of 20 patients, Reader 1 and the independent Reader 2 produced highly concordant lesion masks. Mean Dice Similarity Coefficient was 0.89 (bootstrap 95% CI 0.86–0.92), meeting the pre-specified threshold of ≥0.85. Median 95th-percentile Hausdorff distance was 3.8 mm (IQR 2.5–5.4), within the pre-specified ≤5 mm criterion. The intraclass correlation coefficient ICC(2,1) for segmented volume was 0.93 (95% CI 0.87–0.97), and Bland–Altman 95% limits of agreement for the volume ratio were 0.84–1.22, both within pre-specified criteria. The intra-rater sub-study (Reader 1 re-scoring after 8-week washout, n = 10) yielded Dice 0.93 (95% CI 0.90–0.96), establishing an upper bound on achievable agreement. The pre-specified robustness analysis in which the full multivariable model was re-estimated using Reader 2’s masks yielded an AF disconnection adjusted odds ratio of 1.49 (95% CI 1.21–1.85), overlapping the primary estimate of 1.52 (concordance correlation for per-patient AF disconnection probability ρ_c_ = 0.94). Primary findings were therefore not attributable to single-reader idiosyncrasy.

### 3.3 Longitudinal cognitive trajectories diverge by arcuate fasciculus integrity

Longitudinal tracking across T0 (baseline), T1 (3–6 months), and T2 (median 5.2 years) revealed domain-specific recovery patterns (**Table 3**; full linear mixed-effects model output across all five cognitive domains provided in **Supplementary Table S6**). In the overall cohort, the Language and Executive Function domains demonstrated V-shaped trajectories, with Z-scores declining sharply at T1 and recovering significantly by T2 (both P < 0.001 for main effect of Time). Verbal Memory (HVLT-R Delayed Recall) showed persistent decline with no significant recovery from T1 to T2 (T0 vs. T2 Z-score change = −0.50, P = 0.008, Cohen’s d = 0.42), indicating permanent deficit in this domain.

**Table 3:** Longitudinal trajectories of neuropsychological performance across preoperative, subacute, and long-term follow-up time points.

Cognitive domain/test	T0 (pre-op)	T1 (3–6 mo)	T2 (5 yr)	P (Time) a	P (T0–T2) b	Cohen’s d
Global composite (Z)	−0.24 (0.85)	−1.15 (1.02)	−0.58 (0.94)	<0.001	<0.001	0.38
Language						
BNT raw (max 60)	54.2 (4.5)	46.8 (8.2)	51.5 (6.1)	<0.001	0.003	—
BNT Z-score	−0.32 (0.75)	−1.55 (1.36)	−0.77 (1.01)	<0.001	<0.001	0.51
Verbal fluency raw	18.5 (4.2)	14.1 (5.1)	16.8 (4.8)	<0.001	0.082	—
Verbal fluency Z	−0.15 (0.68)	−1.10 (1.12)	−0.45 (0.92)	<0.001	0.055	0.37
Verbal memory						
HVLT-R immediate raw (22)	24.5 (5.1)	20.2 (6.3)	22.8 (5.8)	<0.001	0.124	—
HVLT-R immediate Z	−0.41 (0.92)	−1.25 (1.45)	−0.72 (1.15)	<0.001	0.041	0.30
HVLT-R delayed raw (12)	8.2 (2.4)	5.8 (3.1)	7.1 (2.9)	<0.001	0.015	—
HVLT-R delayed Z	−0.55 (1.05)	−1.68 (1.52)	−1.05 (1.33)	<0.001	0.008	0.42
Executive function						
TMT-B raw (s) †	85.4 (28.2)	115.6 (42.5)	94.2 (31.4)	<0.001	0.068	—
TMT-B Z	−0.35 (0.88)	−1.30 (1.65)	−0.62 (1.10)	<0.001	0.024	0.27
Attention and processing speed						
DSST raw	48.5 (11.2)	40.2 (12.5)	45.1 (11.8)	<0.001	0.092	—
DSST Z	−0.28 (0.82)	−0.95 (1.05)	−0.52 (0.94)	<0.001	0.076	0.27

Values are presented as mean (standard deviation). Linear mixed-effects models included Time as fixed effect, subject as random effect (random intercept and random slope for time), adjusted for age, education, and tumor volume. a, P (Time): significance of main effect of Time across the three time points, Benjamini–Hochberg FDR-corrected across five cognitive domains. b, P (T0–T2): Bonferroni-corrected pairwise comparison (adjusted α = 0.017). Cohen’s d: standardized effect size for T0–T2 change (Z-scores only). † For TMT-B, higher raw scores (seconds) indicate worse performance; Z-scores were sign-inverted so that negative values consistently indicate impairment. — Not applicable. T0, preoperative baseline; T1, subacute postoperative phase (3–6 months); T2, long-term follow-up (median 5.2 years); BNT, Boston Naming Test; HVLT-R, Hopkins Verbal Learning Test-Revised; TMT-B, Trail Making Test Part B; DSST, Digit Symbol Substitution Test; FDR, false discovery rate.

When patients were stratified by white matter integrity, trajectories diverged (**Figure 4A**; population mean trajectories). The linear mixed-effects model identified a significant Time × Lesion Status interaction for the Language domain (β = 1.24, 95% CI 0.82–1.66, P < 0.001; marginal R^2^ = 0.31, conditional R^2^ = 0.68; **Supplementary Table S6**, Language column). Patients with intact dorsal streams (AF disconnection probability < 0.50, n = 142) followed a V-shaped pattern (T0→T1 Cohen’s d = 1.45; T1→T2 d = 1.12), returning to within 0.3 SD of baseline. Patients with AF disconnection probability ≥0.50 (n = 72) exhibited an L-shaped pattern with sharp postoperative decline (d = 1.52) followed by a plateau with minimal recovery (T1→T2 d = 0.18, P = 0.42; **Figure 4A**). Spaghetti plots of individual trajectories confirmed that the absence of recovery was systemic within the disconnected group rather than driven by outliers (**Figures 4B, C**).

**Figure 4:**
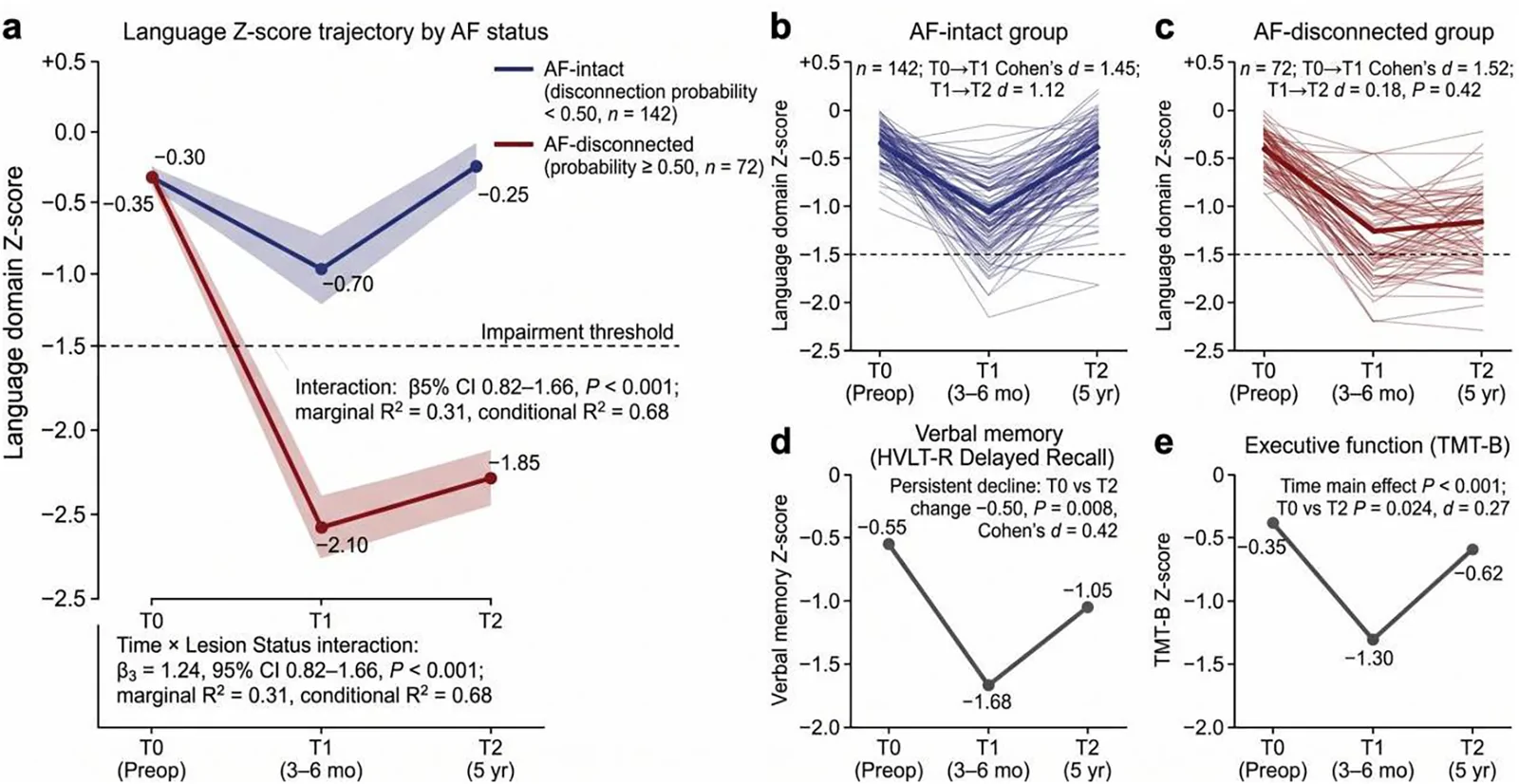
Longitudinal cognitive trajectories diverge by arcuate fasciculus integrity: “V-shaped” versus “L-shaped” recovery patterns. **(a)** Group-level language Z-score trajectories across the three assessment time points (T0 preoperative, T1 subacute 3–6 months postoperative, T2 long-term ≥ 4 years), stratified by arcuate fasciculus (AF) disconnection probability (< 0.50, “AF-Intact”, n = 142, blue; ≥ 0.50, “AF-Disconnected”, n = 72, red). Solid lines represent group means with shaded 95% confidence intervals derived from linear mixed-effects modeling (random intercept and random slope per subject). Annotations display Cohen’s d effect sizes for T0→T1 and T1→T2 transitions within each group, and the Time × Lesion-Status interaction P-value (P < 0.001). **(b)** Individual patient trajectories (spaghetti plot) for the AF-Intact group, demonstrating broadly homogeneous “V-shaped” recovery across patients. **(c)** Individual patient trajectories for the AF-Disconnected group, demonstrating the characteristic “L-shaped” pattern of sharp initial decline followed by a plateau with minimal recovery. Thick black lines overlay group means for reference. All Z-scores are age-, sex-, and education-adjusted relative to a regional Chinese normative sample (n = 500). The Time × AF-Status interaction coefficient (β3 = 1.24, 95% CI 0.82–1.66, P < 0.001) is derived from the primary linear mixed-effects model with marginal R2 = 0.31 and conditional R2 = 0.68. **(d)** Verbal memory (HVLT-R delayed recall) Z-score trajectory in the overall cohort, showing persistent decline without significant recovery from T1 to T2 (T0 versus T2 change −0.50, P = 0.008, Cohen’s d = 0.42). **(e)** Executive function (Trail Making Test B) Z-score trajectory in the overall cohort (main effect of Time P < 0.001; T0 versus T2 P = 0.024, Cohen’s d = 0.27). T0, preoperative baseline; T1, subacute postoperative phase (3–6 months); T2, long-term follow-up (median 5.2 years); AF, arcuate fasciculus; CI, confidence interval.

The Time × Lesion Status interaction remained statistically significant across all five cognitive domains after FDR correction (all q ≤ 0.010; **Supplementary Table S6**). IPW-adjusted estimates were concordant with the primary unweighted models: Language Time × Lesion β = 1.18 after P1/P99 weight truncation and 1.21 after P5/P95 truncation (primary β = 1.24), Memory β = 0.78 and 0.82 (primary β = 0.85), and Executive Function β = 0.88 and 0.90 (primary β = 0.92); all P < 0.001. After P1/P99 truncation, weights had mean 1.12, SD 0.48, median 1.01, and range 0.28–3.85; four observations were capped. After P5/P95 truncation, weights had mean 1.08, SD 0.35, median 0.99, and range 0.35–2.92; 20 observations were capped. **Supplementary Figure S7** also presents covariate-balance and effective sample-size diagnostics.

### 3.4 Voxel-based lesion-symptom mapping identifies domain-specific topographies

VLSM analysis identified distinct voxel clusters significantly associated with LTCD after FDR correction at q < 0.05 (**Figure 5**; cluster statistics in **Table 4**). For the Language domain, significant clusters mapped to the left posterior inferior frontal gyrus (Broca area), the posterior superior temporal gyrus (Wernicke area), and the subcortical white matter tracing the hook-shaped trajectory of the arcuate fasciculus (peak Z = 6.42 at MNI coordinates [−38, −12, 24]; cluster volume 4,250 mm^3^; **Figure 5A**). For the Verbal Memory domain, significant clusters were anatomically dissociated from those for Language, localizing to the left medial temporal lobe — hippocampus, parahippocampal gyrus, and uncinate fasciculus (peak Z = 4.95 at [−28, 4, −18]; cluster volume 2,340 mm^3^; **Figure 5B**). For Executive Function, significant clusters involved the bilateral Frontal Aslant Tract with left-hemispheric dominance (left Z = 5.88 vs. right Z = 4.12; **Table 4**, Executive Function rows).

**Figure 5:**
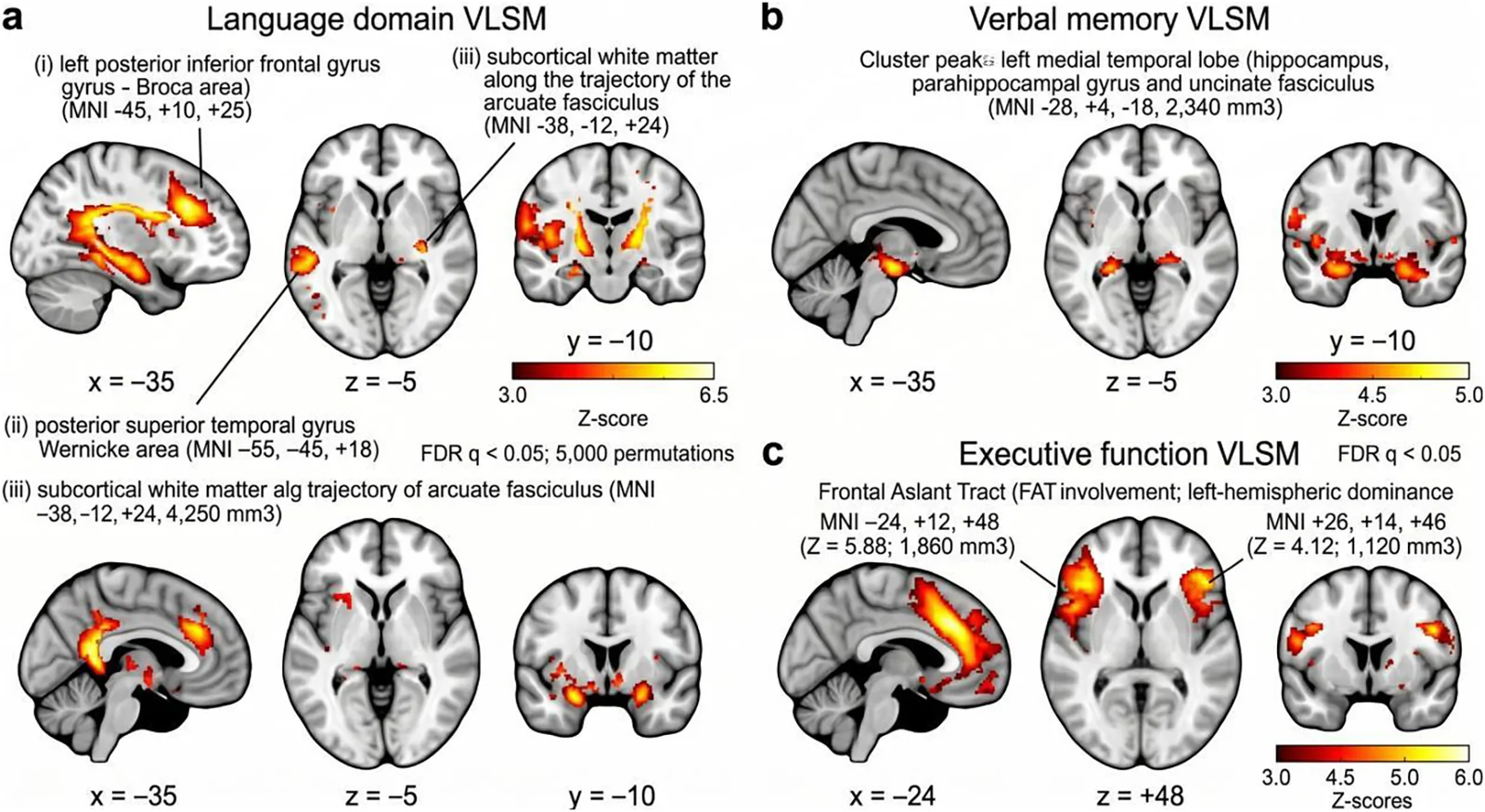
Voxel-based lesion-symptom mapping identifies anatomically distinct substrates for long-term language, verbal memory, and executive function impairments. **(a)** Significant voxel clusters associated with 5-year language Z-scores following voxel-based lesion-symptom mapping (VLSM) with Brunner–Munzel non-parametric testing and 5,000-permutation FDR correction at q < 0.05. Hot-color overlay on the MNI152 template identifies three clusters in the left hemisphere: posterior inferior frontal gyrus (Broca’s area), posterior superior temporal gyrus (Wernicke’s area), and the subcortical white matter tracing the hook-shaped trajectory of the arcuate fasciculus. Peak Z-score 6.42 at MNI coordinates [−38, −12, 24]; cluster volume 4,250 mm3. **(b)** Significant voxel clusters associated with 5-year verbal memory (HVLT-R delayed recall) Z-scores, mapping to the left medial temporal lobe — specifically the hippocampus, parahippocampal gyrus, and the rostral fibers of the uncinate fasciculus. Peak Z-score 4.95 at MNI coordinates [−28, 4, −18]; cluster volume 2,340 mm3. **(C)** Significant voxel clusters associated with 5-year executive function Z-scores, involving the bilateral frontal aslant tract with left-hemispheric dominance (left peak Z = 5.88 at [−24, 12, 48]; right peak Z = 4.12 at (14, 23, 24)). Lesion volume was included as a covariate in all analysis to control for lesion-size confounding. Color bars represent Z-score magnitude. Axial slices displayed at standardized z-coordinates; L = left, R = right. VLSM, voxel-based lesion-symptom mapping; FDR, false discovery rate; HVLT-R, Hopkins Verbal Learning Test-Revised; MNI, Montreal Neurological Institute.

**Table 4:** Anatomical specificity of significant white matter clusters associated with long-term cognitive deficits (VLSM and CLSM results).

Cognitive domain/tract	Hemi	Cluster (mm³)	Peak MNI (x, y, z)	Peak Z	Overlap prob.	R (CLSM)	Core hub
Language (naming and fluency)							
Arcuate Fasciculus (AF)	L	4,250	−38, −12, 24	6.42	92%	−0.58	Yes
IFOF	L	2,180	−34, 18, −12	5.15	85%	−0.32	Yes
ILF	L	1,450	−42, −58, −8	3.84	76%	−0.21	No
SLF-III	L	980	−44, 8, 22	3.25	68%	−0.18	No
Executive function							
Frontal Aslant Tract (FAT)	L	1,860	−24, 12, 48	5.88	94%	−0.45	Yes
Frontal Aslant Tract (FAT)	R	1,120	26, 14, 46	4.12	81%	−0.28	Yes
Anterior thalamic radiation	L	850	−18, 26, 14	3.56	62%	−0.22	No
Cingulum bundle (dorsal)	L	640	−8, −14, 38	3.10	55%	−0.15	No
Verbal memory							
Uncinate Fasciculus (UF)	L	2,340	−28, 4, −18	4.95	88%	−0.41	Yes
Fornix (crus)	L	420	−12, −34, 10	4.42	95%	−0.38	Yes
Ventral amygdalofugal	L	380	−22, −4, −16	3.15	60%	−0.19	No
Visuospatial attention							
SLF-II	R	1,650	36, −42, 34	4.78	78%	−0.35	No
Optic radiation	R	1,210	32, −64, 4	3.65	70%	−0.24	No

All coordinates refer to the Montreal Neurological Institute (MNI152) standard space. Results represent significant clusters surviving false discovery rate correction at q < 0.05 (5,000 permutations). Cluster size: volume of the significant lesion cluster intersecting the specific white matter tract. Overlap probability: maximum probabilistic likelihood (from the HCP-842 atlas) that the identified cluster belongs to the specified tract. R (CLSM): Spearman correlation coefficient between tract-level disconnection probability and 5-year cognitive Z-score. Core hub: defined as Peak Z > 4.0 AND overlap > 80%, identifying non-redundant neural substrates critical for long-term recovery. L, left hemisphere; R, right hemisphere; AF, arcuate fasciculus; IFOF, inferior fronto-occipital fasciculus; ILF, inferior longitudinal fasciculus; SLF, superior longitudinal fasciculus; FAT, frontal aslant tract; UF, uncinate fasciculus; CLSM, connectome-based lesion-symptom mapping; VLSM, voxel-based lesion-symptom mapping.

### 3.5 Connectome-based disconnection and DTI cross-validation

Connectome-based lesion-symptom mapping projected individual lesions onto the HCP-842 normative connectome to generate a group-level probabilistic disconnection map (**Figure 1a**). AF disconnection probability showed the strongest negative association with the 5-year language Z-score (Spearman ρ = −0.58, P < 0.001; **Figure 1b**). IFOF disconnection showed a weaker association (ρ = −0.32, P = 0.012; **Figure 1c**), identifying the AF as the principal structural bottleneck for long-term language recovery in this cohort.

Validation against patient-specific diffusion tensor imaging in the 85-patient DTI sub-cohort showed inverse correlations between CLSM-derived disconnection probability and fractional anisotropy for the AF (Spearman ρ = −0.72, P < 0.001; **Figure 1d**) and UF (ρ = −0.61, P < 0.001; **Figure 1e**). Bland–Altman analysis showed minimal systematic bias (AF mean bias +2.4, 95% limits of agreement −18.0 to +22.8; UF mean bias +3.8, 95% limits of agreement −20.9 to +28.5; **Supplementary Figure S1**), supporting the use of the normative Disconnectome approach when individual diffusion imaging is unavailable.

### 3.6 Anatomical dissociation between transient and permanent deficits

A spatial dissociation was observed between lesion topographies associated with transient (T1) and permanent (T2) deficits (**Figure 6**). Lesions associated with transient deficits (**Figure 6A**) were spatially extensive, predominantly affecting cortical gray matter — particularly the supplementary motor area and superior frontal gyrus — and superficial U-fibers. In contrast, lesions associated with permanent deficits (**Figure 6B**) were spatially focal and strictly confined to the deep white matter skeleton. The overlay map (**Figure 6C**) demonstrated a core–periphery organization: cortical damage permits functional remodeling through perilesional or contralateral compensation, whereas damage to deep bottleneck hubs — specifically the stem of the AF and the subcortical limbic entry points — was associated with irreversible cognitive decline.

**Figure 6:**
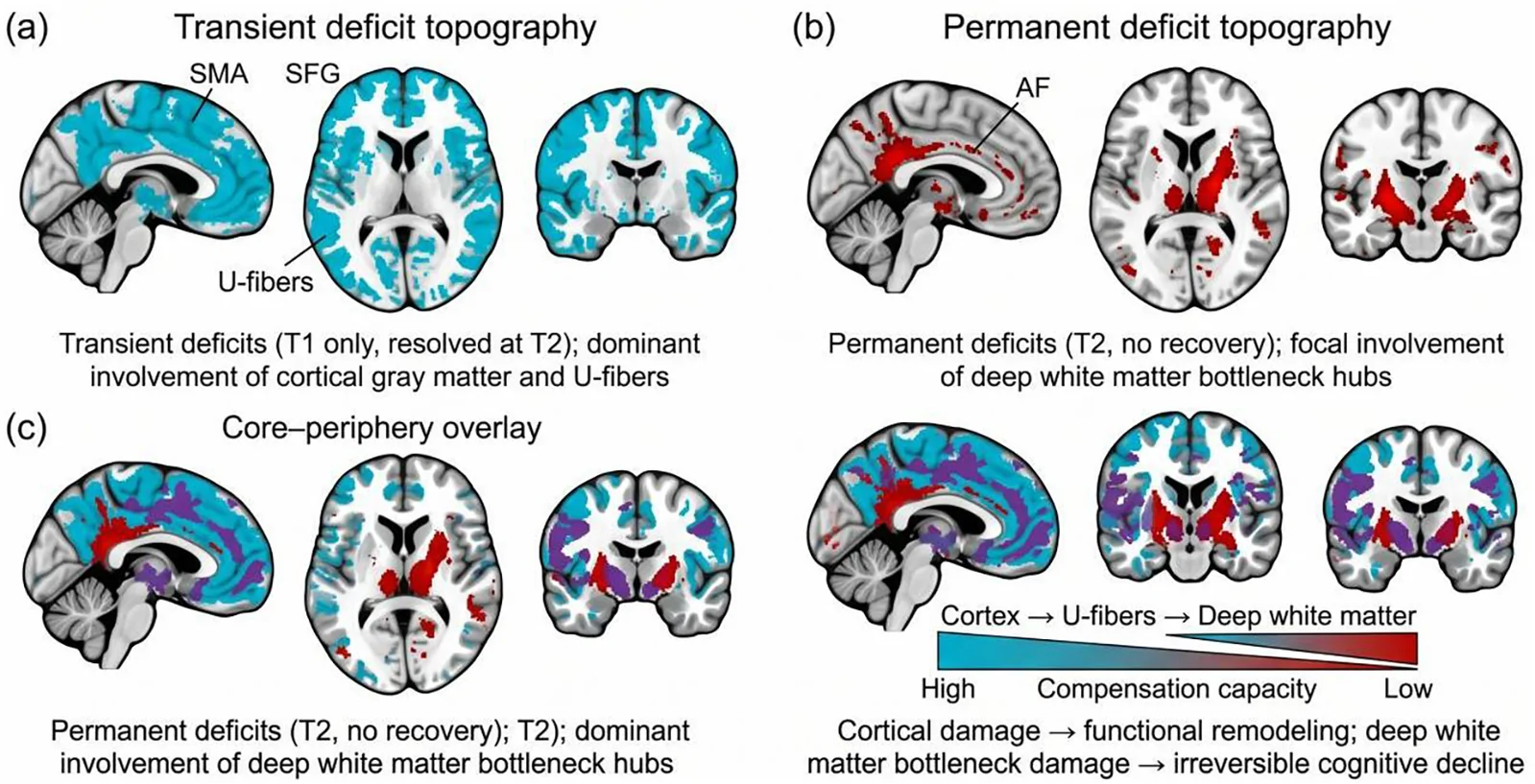
Spatial dissociation between lesion topographies associated with transient versus permanent cognitive deficits reveals a core–periphery organization. **(a)** Composite lesion map (cyan) for patients who demonstrated cognitive deficits at T1 (subacute phase) but recovered by T2 (long-term follow-up), n = 68. The spatially extensive topography predominantly involves cortical gray matter — most notably the supplementary motor area, superior frontal gyrus, and pre/postcentral gyri — and superficial U-fibers. **(b)** Composite lesion map (red) for patients who exhibited persistent cognitive deficits at T2 without intervening recovery, n = 88. In contrast to panel A, the topography is spatially focal and strictly confined to deep white matter, including the stem of the arcuate fasciculus, the uncinate fasciculus, and subcortical limbic entry points. **(c)** Overlay map with cyan and red regions superimposed, revealing a “Core–Periphery” organization: cortical damage (periphery) is amenable to functional remodeling through perilesional or contralateral compensation, whereas damage to deep bottleneck hubs (core) results in irreversible cognitive decline. Color intensity reflects the number of overlapping patient lesions at each voxel. All projections are on the MNI152 template; axial, sagittal, and coronal slices at standardized coordinates. SMA, supplementary motor area; UF, uncinate fasciculus; AF, arcuate fasciculus; T1, subacute postoperative phase; T2, long-term follow-up.

### 3.7 Multiplex immunofluorescence defines six marker-based glioma-associated myeloid states

#### 3.7.1 Unsupervised clustering resolves six stable meta-clusters

The 6-plex Opal multiplex immunofluorescence assay was performed on FFPE tumor sections from 186 patients. A total of 2.41 × 10^6^ CD68+ or Iba1+ cells from tumor core, peri-tumoral white matter, and infiltrative margin were subjected to FlowSOM meta-clustering and UMAP visualization (**Figure 2a**). Six marker-defined meta-clusters were resolved: C1, CD68lo CD163lo CD206lo TREM2lo (11.5%); C2, CD68hi iNOShi CD163lo CD206lo (14.0%); C3, CD163hi CD206hi (22.4%); C4, TREM2hi with all other panel markers intermediate (17.1%); C5, CD163hi iNOShi dual-high (20.6%); and C6, Iba1hi CD68lo (14.4%). The names describe measured marker profiles and do not imply ontogeny or formal M1/M2 identity. The heat map in **Figure 2b** shows the cluster-median marker-intensity matrix; C4 had the highest TREM2 z-score (+2.10), whereas the next-highest cluster had a TREM2 z-score of +0.15.

C5 reaches the high tier simultaneously for an alternative-activation marker (CD163) and a classical-activation marker (iNOS), demonstrating co-expression that a mutually exclusive M1/M2 assignment cannot accommodate, while C3 and C4 are separately resolved on CD163/CD206 and on TREM2 respectively. These observations concern the adequacy of a descriptive classification and are consistent with a continuum model of myeloid activation; they do not by themselves establish a functional continuum.

Orthogonal chromogenic P2RY12 immunohistochemistry on adjacent sections was available in all 186 cases. Expressed as a ratio to Iba1-positive cell density in the same compartment, the P2RY12^+^ fraction was highest at the infiltrative margin (median 54.2% of Iba1^+^ cells), intermediate in peri-tumoral white matter (48.6%) and lowest in the tumour core (22.4%; Friedman P < 0.001); the corresponding absolute densities were 31.8, 34.6 and 8.6 cells per high-power field. Absolute density peaks in peri-tumoral white matter because total myeloid cellularity is highest there, whereas the P2RY12^+^ fraction is lowest in the tumour core — that is, among the myeloid cells present, fewer retain the homeostatic marker in the core than at the tumour–brain interface or the infiltrative margin. The two quantities answer different questions, and their divergence illustrates why absolute density is not comparable across zones of differing cellularity.

Patient-level Spearman correlations between the P2RY12+/Iba1+ ratio and the per-patient proportion of each meta-cluster were: C1, ρ = +0.28 (P = 0.0001); C6, ρ = +0.22 (P = 0.0022); C2, ρ = −0.11 (P = 0.133); C3, ρ = −0.15 (P = 0.040); C4, ρ = −0.18 (P = 0.013); and C5, ρ = −0.06 (P = 0.415). Because the six proportions sum to unity, these correlations are compositionally constrained (standard deviations of the cluster proportions 0.042, 0.048, 0.065, 0.058, 0.052, and 0.046 for C1–C6, respectively). The negative association with C4 was directionally consistent with lower P2RY12 expression in TREM2-high states, although part of any negative correlation in compositional data is structural. These regional findings do not constitute cell-level or ontogenetic validation of any meta-cluster.

#### 3.7.2 Cluster validation and stability of the TREM2-high population

Across k = 4–12 the proportion of ambiguous clustering was minimised at k = 6 (PAC 0.042); mean silhouette width at k = 6 was 0.48; and the relative change in consensus-CDF area fell from 0.35 at k = 5 to 0.12 at k = 6 and 0.04 at k = 7, placing the elbow at k = 6 (**Supplementary Table S10**). All cluster pairs satisfied the pre-specified non-redundancy threshold, the smallest pairwise Euclidean distance being 1.97 (C1 versus C6) and the next smallest 2.85; the criterion became binding at k = 7, where two clusters lay 0.98 apart. Bootstrap cluster-wise stability across 500 patient-level resamples gave mean Jaccard coefficients of 0.84 (C1), 0.81 (C2), 0.88 (C3), 0.86 (C4), 0.78 (C5) and 0.82 (C6), all above the conventional 0.75 threshold, and the pairwise adjusted Rand index across 10 random seeds was 0.89 (range 0.84–0.93). Re-running the pipeline on a 10 × 10 grid returned the same partition (adjusted Rand index 0.94).

The two substantive myeloid findings were robust to the clustering solution. Under every alternative solution in the range k = 5–9, under 10 random seeds, and under Leiden clustering of the same matrix, the TREM2-high population — re-identified in each solution as the meta-cluster with the highest median TREM2 z-score — remained enriched at the peri-tumoral white matter interface relative to the tumour core (enrichment range +6.1 to +8.4 percentage points, all P < 0.001; primary estimate +7.2 percentage points), and its per-patient proportion remained associated with arcuate fasciculus disconnection probability after adjustment for the recomputed GAM-PCI (β range 0.18 to 0.24 per 10% proportion, all P < 0.01) (**Supplementary Table S11**).

#### 3.7.3 Cell yield per patient and per anatomical zone

The median yield was 12,950 retained myeloid cells per patient (IQR 9,800–16,200; range 3,120–22,400) and 4,310 per patient–zone unit across 558 units (IQR 3,100–5,500; minimum 840). No patient–zone unit fell below either pre-specified threshold of 200 or 500 cells. Across the 3,348 zone × meta-cluster combinations the median contribution was 745 cells (IQR 420–1,180; 5th percentile 112; minimum 28), and 42 combinations distributed across 31 patient–zone units in 18 patients contained fewer than 50 cells, 11 of those units carrying two or more low-count clusters (**Supplementary Table S13**). Because no unit fell below the total-count thresholds, sensitivity was assessed at the level at which proportional estimates are least stable, by excluding the 31 patient–zone units containing any meta-cluster with fewer than 50 cells. Estimates were materially unchanged: the peri-tumoral versus tumour-core proportion of the TREM2-high cluster was 20.9% versus 13.9% (enrichment +7.0 percentage points, paired Wilcoxon P < 0.001) against a primary estimate of 21.0% versus 13.8% (+7.2 percentage points, P < 0.001), and the multivariable association of its proportion with arcuate fasciculus disconnection probability was β = 0.21 (95% CI 0.07–0.35, P = 0.003) against a primary β = 0.21 (95% CI 0.08–0.34, P = 0.002) (**Supplementary Table S14**).

#### 3.7.4 Spatial distribution along the tumor–brain interface

Meta-cluster proportions differed by anatomical zone (**Figure 2c**, **Table 2**). **Figure 2c** displays the paired C3 comparison between tumor core and peri-tumoral white matter (17.6% vs. 26.9%, P < 0.001) and the paired C2 comparison between tumor core and infiltrative margin (11.2% vs. 19.3%, P = 0.001). **Table 2** additionally shows enrichment of C4 in peri-tumoral white matter relative to tumor core (21.0% vs. 13.8%, P < 0.001). The Morisita–Horn colocalization index between CD163+CD206+ cells and MBP+ fragments was 0.71 [0.58–0.82] in peri-tumoral white matter and 0.42 [0.31–0.55] at the infiltrative margin (P < 0.001). The mean nearest-neighbor distance from CD163+CD206+ cells to the peri-tumoral white matter boundary was 38 ± 14 µm, compared with a null median of 87 µm (P < 0.001). These spatial analyses were performed on CD163+CD206+ cells, not on the C4 cluster, and the distance endpoint was the white matter boundary rather than an MBP fragment.

**Supplementary Figure S2** presents the patient-level Spearman matrix relating six peri-tumoral immune measures to disconnection probabilities across AF, IFOF, UF, SLF, and FAT, together with an AF-specific summary. The strongest reported AF associations were observed for CD163+ density (ρ = 0.42, P < 0.001), Iba1+ density (ρ = 0.38, P < 0.001), CD68+ density (ρ = 0.35, P < 0.001), and the legacy CD163+/CD68+ ratio (ρ = 0.38, P < 0.001). The legacy ratio was also associated with IFOF disconnection (ρ = 0.26, P < 0.05). CD3+ and CD8+ densities had no reported association with the five tracts (all |ρ| ≤ 0.12). These data support marker-specific associations but do not establish immune-cell causation.

#### 3.7.5 GAM polarization continuum index is independently associated with AF disconnection

The per-patient GAM polarization continuum index (GAM-PCI; first principal component of the meta-cluster proportion vector, oriented so that higher values indicate M2/TREM2-skewed composition) captured 47.2% of total variance in myeloid composition. GAM-PCI was inversely correlated with the iNOS^+^ cluster proportion (Spearman ρ = −0.83, P < 0.001), confirming its intended directionality. The per-patient M2/M1 ratio computed from conventional CD163^+^/CD68^+^ staining correlated strongly with GAM-PCI (ρ = 0.78, P < 0.001). GAM-PCI was more strongly associated with AF disconnection probability (Pearson R = 0.45, P < 0.001; **Figure 2D**) than the legacy M2/M1 ratio (R = 0.38, P < 0.001), indicating that the continuum representation captures prognostically relevant information lost in dichotomous reduction. In multivariable linear regression adjusting for tumor volume, WHO grade, and IDH status, GAM-PCI was independently associated with AF disconnection probability (β = 0.34 per SD, 95% CI 0.21–0.47, P < 0.001). The proportion of the C4 TREM2^hi^ cluster was an predictor of AF disconnection beyond overall GAM-PCI (β = 0.21 per 10% proportion, 95% CI 0.08–0.34, P = 0.002).

### 3.8 Peri-tumoral myelin injury and matrix remodeling link myeloid polarization to disconnection

Chromogenic immunohistochemistry for MBP, MMP-9, and Olig2 was performed on serial tissue sections. **Figure 2g** shows patient-level distributions by AF status. The MBP-loss index, calculated in 162 cases with evaluable contralateral white matter, was 0.12 [0.05–0.22] in AF-intact patients and 0.34 [0.22–0.48] in AF-disconnected patients (P < 0.001). Peri-tumoral MMP-9+ cell density was 26.4 (10.2) versus 48.2 (15.6) cells/HPF (P < 0.001), and Olig2+ density was 42.6 (13.8) versus 31.8 (11.2) cells/HPF (P = 0.008), respectively. The MBP-loss index was associated with GAM-PCI (Spearman ρ = 0.52, P < 0.001), and MMP-9+ density was associated with GAM-PCI (ρ = 0.47, P < 0.001). When the 5-year language Z-score was regressed simultaneously on GAM-PCI, MBP-loss index, and AF disconnection probability, the MBP-loss index retained independent significance (β = −0.21, 95% CI −0.33 to −0.09, P = 0.001) alongside AF disconnection probability (β = −0.28 per 10% increment, P < 0.001), while GAM-PCI lost significance (β = −0.06, P = 0.42) — a pattern of effect attenuation consistent with the hypothesized serial mediation pathway (GAM-PCI → MBP-loss → AF disconnection → cognitive outcome).

### 3.9 Longitudinal serum biomarker profiles

#### 3.9.1 Core panel

In the biomarker sub-cohort (n = 72; AF-intact n = 48; AF-disconnected n = 24), **Figures 7a–c** shows NfL, GFAP, and IL-6 trajectories across T0, T1, and T2. At T2, median NfL was 16.5 [10.8–24.1] versus 28.4 [18.2–42.6] pg/mL (P = 0.002), GFAP was 124.5 [72.4–215.8] versus 198.2 [108.5–345.6] pg/mL (P = 0.008), and IL-6 was 4.2 [2.5–7.8] versus 8.5 [4.8–14.2] pg/mL (P = 0.006), comparing AF-intact with AF-disconnected patients. Concentrations were log10-transformed for modeling; **Figure 7** displays the reported concentration scale. TNF-α and IL-1β showed significant main effects of Time (both P < 0.001) but non-significant Time × Group interactions (TNF-α P = 0.085; IL-1β P = 0.068). At T2, median TNF-α was 5.8 pg/mL in the AF-intact group and 7.8 pg/mL in the AF-disconnected group (P = 0.032), and median IL-1β was 1.5 vs. 2.8 pg/mL (P = 0.041; **Table 5**; **Supplementary Figures S3a, b**). Cross-sectional associations with the 5-year Language Z-score were weak and not statistically significant (TNF-α: ρ = −0.18, P = 0.13; IL-1β: ρ = −0.22, P = 0.06; **Supplementary Figures S3c, d**). The complete Spearman correlation matrix is presented in **Supplementary Table S7**, with the signed correlation coefficient, pairwise-complete sample size, and numerical two-sided P value reported for each analysis. NfL showed the largest correlation with AF disconnection probability (ρ = 0.48, P = 0.000020) and the largest inverse correlation with the 5-year Language Z-score (ρ = −0.42, P = 0.000240). IL-6 was correlated with AF disconnection probability (ρ = 0.35, P = 0.002580), whereas its correlation with the Language Z-score was smaller (ρ = −0.24, P = 0.042295). The AF disconnection correlations were ρ = 0.38 for GFAP (P = 0.000993), ρ = 0.32 for S100B (P = 0.006140), ρ = 0.22 for TNF-α (P = 0.063324), and ρ = 0.25 for IL-1β (P = 0.034179).

**Figure 7:**
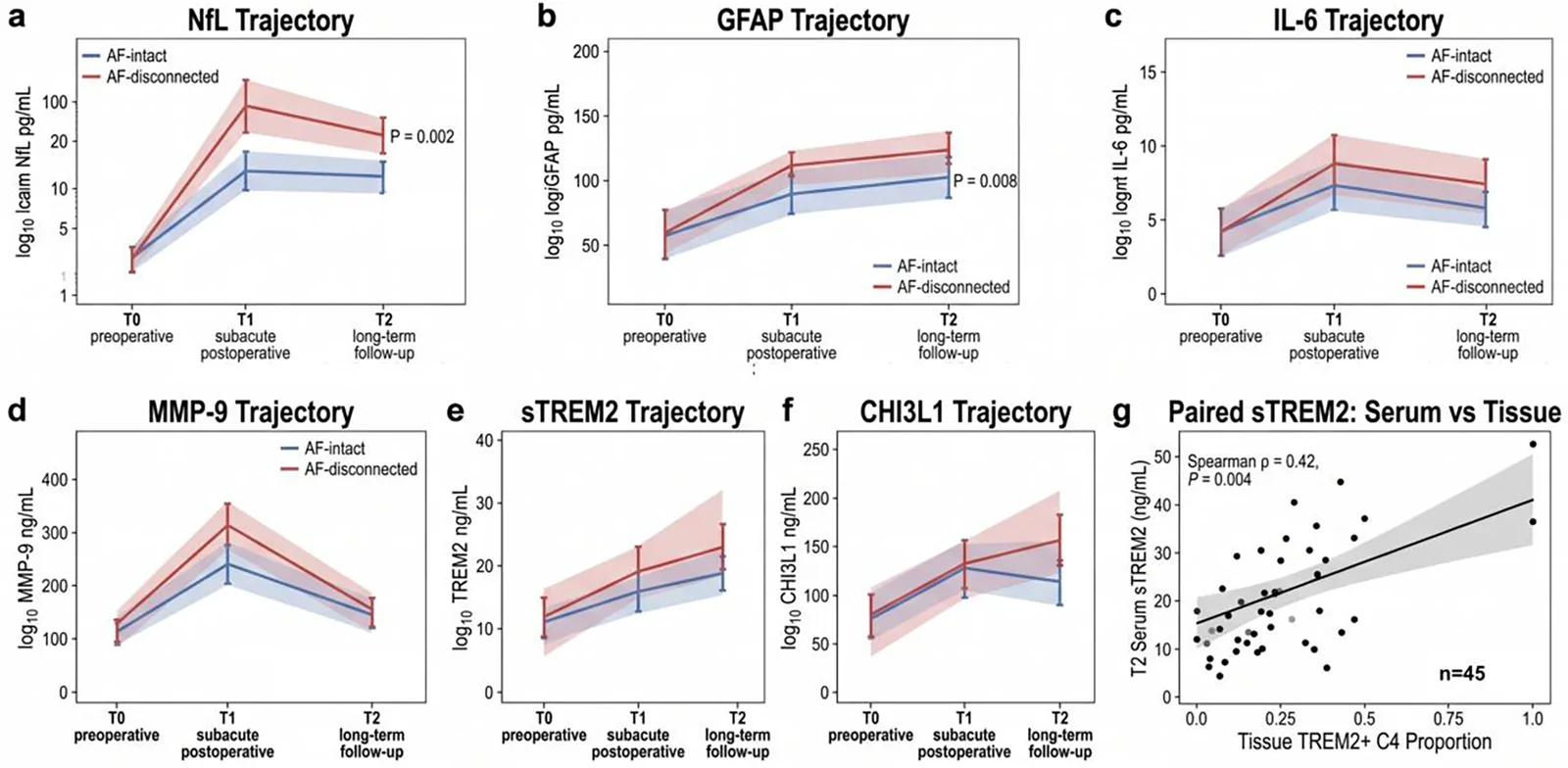
Longitudinal serum neuroimmune biomarker trajectories and the paired serum–tissue sTREM2 association. The biomarker sub-cohort comprised 72 patients (AF-intact, n = 48; AF-disconnected, n = 24) with samples at T0, T1, and T2. **(a)** NfL trajectory; T2 medians were 16.5 [10.8–24.1] and 28.4 [18.2–42.6] pg/mL (P = 0.002). **(b)** GFAP trajectory; T2 medians were 124.5 [72.4–215.8] and 198.2 [108.5–345.6] pg/mL (P = 0.008). **(c)** IL-6 trajectory; T2 medians were 4.2 [2.5–7.8] and 8.5 [4.8–14.2] pg/mL (P = 0.006). **(d)** MMP-9 trajectory; T1 medians were 268 and 425 ng/mL (P < 0.001), whereas the T2 difference was not significant (P = 0.31). **(e)** sTREM2 trajectory; T2 medians were 11.2 and 18.4 ng/mL (P = 0.003). **(f)** CHI3L1 trajectory; T2 medians were 82 and 148 ng/mL (P < 0.001). Values are listed as AF-intact followed by AF-disconnected. **(g)** Complete-pair serum–tissue analysis in 45 patients: T2 serum sTREM2 versus peri-tumoral C4 proportion (Spearman ρ = 0.42, P = 0.004). This association does not establish the cellular origin of circulating sTREM2. AF, arcuate fasciculus; CHI3L1, chitinase-3-like protein 1; GFAP, glial fibrillary acidic protein; IL-6, interleukin-6; MMP-9, matrix metalloproteinase-9; NfL, neurofilament light chain; sTREM2, soluble triggering receptor expressed on myeloid cells 2; T0, preoperative; T1, 3–6 months postoperative; T2, long-term follow-up.

**Table 5:** Serum neuroimmune biomarker concentrations at three time points stratified by Arcuate Fasciculus disconnection status (n = 72), extended panel.

Biomarker	T0 AF-Int (n=48)	T0 AF-Dis (n=24)	T1 AF-Int	T1 AF-Dis	T2 AF-Int/AF-Dis	P (Time) a	P (Int.) b
Core panel: neuroaxonal and pro-inflammatory							
NfL, pg/mL	17.5 [11.8–25.2]	19.4 [13.5–28.8]	38.2 [24.1–58.6]	52.4 [35.8–82.5]	16.5 [10.8–24.1]/28.4 [18.2–42.6]	<0.001	0.008
GFAP, pg/mL	85.2 [48.5–142.8]	92.4 [52.1–158.5]	245.6 [128.4–468.2]	342.8 [185.2–585.4]	124.5 [72.4–215.8]/198.2 [108.5–345.6]	<0.001	0.024
S100B, µg/L	0.06 [0.04–0.09]	0.07 [0.04–0.10]	0.15 [0.08–0.28]	0.22 [0.12–0.38]	0.08 [0.05–0.12]/0.14 [0.08–0.22]	<0.001	0.042
IL-6, pg/mL	3.8 [2.1–6.2]	4.2 [2.4–7.1]	11.5 [6.8–18.4]	15.2 [8.5–24.8]	4.2 [2.5–7.8]/8.5 [4.8–14.2]	<0.001	0.018
TNF-α, pg/mL	5.2 [3.4–8.1]	5.8 [3.8–8.5]	8.4 [5.2–12.8]	10.2 [6.5–15.4]	5.8 [3.6–8.5]/7.8 [4.8–11.2]	<0.001	0.085
IL-1β, pg/mL	1.2 [0.6–2.4]	1.4 [0.7–2.8]	3.5 [1.8–6.2]	4.8 [2.4–8.5]	1.5 [0.8–2.8]/2.8 [1.4–5.2]	<0.001	0.068
Extended panel: matrix remodeling, immunosuppression, DAM activation, glial cross-talk							
MMP-9, ng/mL	182 [115–268]	195 [122–285]	268 [168–425]	425 [285–612]	142 [88–215]/168 [105–248]	<0.001	<0.001
Active TGF-β1, ng/mL	8.2 [5.4–12.8]	8.8 [5.8–13.4]	14.2 [9.5–22.4]	19.8 [13.5–28.6]	11.5 [7.8–17.4]/18.2 [12.4–26.8]	<0.001	0.006
IL-10, pg/mL	1.8 [0.9–3.2]	2.0 [1.0–3.5]	4.5 [2.4–7.8]	6.2 [3.5–10.4]	2.6 [1.4–4.5]/4.2 [2.2–7.1]	<0.001	0.024
sTREM2, ng/mL	9.8 [6.4–14.2]	10.4 [6.8–15.1]	14.5 [9.8–22.4]	18.6 [12.4–28.5]	11.2 [7.5–16.8]/18.4 [12.4–27.6]	<0.001	0.003
CHI3L1, ng/mL	68 [42–105]	72 [45–112]	115 [72–188]	168 [102–258]	82 [52–128]/148 [92–228]	<0.001	<0.001

Values are presented as median [interquartile range]. The biomarker sub-cohort comprised 72 patients with banked serum at all three time points; AF-Intact (disconnection probability < 0.50) n = 48; AF-Disconnected (probability ≥ 0.50) n = 24. Assay platforms: NfL, GFAP, sTREM2, and CHI3L1 on Simoa HD-X (Quanterix); S100B by ECLIA (Roche Elecsys); IL-6, TNF-α, IL-1β, MMP-9, TGF-β1, IL-10 by Luminex xMAP (Bio-Rad Bio-Plex Pro). All samples analyzed in duplicate; intra- and inter-assay CVs < 15%. Values below LLOQ were imputed as LLOQ/√2; all analytes were log_10_-transformed prior to modeling. a P (Time): Friedman’s non-parametric repeated-measures ANOVA across T0, T1, T2 within the total biomarker cohort. b P (Interaction): Time × Group interaction term from aligned-rank-transform two-way ANOVA. Mann–Whitney U comparisons at T2 between AF-Intact and AF-Disconnected yielded the following P-values: NfL = 0.002, GFAP = 0.008, S100B = 0.015, IL-6 = 0.006, TNF-α = 0.032, IL-1β = 0.041, MMP-9 = 0.31 (not significant at T2; peak at T1), TGF-β1 = 0.006, IL-10 = 0.024, sTREM2 = 0.003, CHI3L1 < 0.001. Bold P-values indicate statistical significance (P < 0.05). NfL, neurofilament light chain; GFAP, glial fibrillary acidic protein; S100B, S100 calcium-binding protein B; IL-6/10/1β, interleukin-6/10/1β; TNF-α, tumor necrosis factor-alpha; MMP-9, matrix metalloproteinase-9; TGF-β1, transforming growth factor-beta 1 (active form, post acid activation); sTREM2, soluble triggering receptor expressed on myeloid cells 2; CHI3L1, chitinase-3-like protein 1 (YKL-40); ECLIA, electrochemiluminescence immunoassay; LLOQ, lower limit of quantification; CV, coefficient of variation.

#### 3.9.2 Extended panel

**Figures 7d–f** shows the longitudinal MMP-9, sTREM2, and CHI3L1 trajectories. MMP-9 peaked at T1 and was higher in AF-disconnected patients (425 vs. 268 ng/mL, P < 0.001), but the T2 between-group difference was not significant (P = 0.31). At T2, sTREM2 was 18.4 vs. 11.2 ng/mL (P = 0.003) and CHI3L1 was 148 vs. 82 ng/mL (P < 0.001), comparing AF-disconnected with AF-intact patients. **Figure 7g** includes the 45 patients with both evaluable T2 serum sTREM2 and peri-tumoral C4 measurements; the association was Spearman ρ = 0.42 (P = 0.004). This complete-pair cross-compartment association was not imputed and does not identify the cellular source of circulating sTREM2. The trajectory comparison supported by **Figure 7** and **Table 5** remains unchanged: T2 IL-6 was 4.2 vs. 8.5 pg/mL in AF-intact and AF-disconnected patients, respectively (P = 0.006). In the multivariable models presented in **Supplementary Table S9**, higher log10-transformed IL-6 was associated with a lower Language Z-score after adjustment for age, education, radiotherapy dose, AF disconnection probability, and the other serum biomarkers (β = −0.24, SE = 0.100, 95% CI −0.44 to −0.04; two-sided P = 0.019). Corresponding adjusted associations were observed for the Memory Z-score (β = −0.22, SE = 0.100, 95% CI −0.42 to −0.02; P = 0.032) and Global Composite (β = −0.22, SE = 0.090, 95% CI −0.40 to −0.04; P = 0.018).

### 3.10 Sequential mediation analysis of the hypothesized myeloid–myelin–disconnection–cognition pathway

The tissue-connectomic model included 162 complete cases (**Figure 8b**). The total adjusted association between GAM-PCI and the 5-year Language Z-score was β = −0.38 per SD (bootstrap 95% CI, −0.52 to −0.24; P < 0.001), and the estimated serial indirect association through peri-tumoral MBP loss and AF disconnection was β = −0.19 (95% CI, −0.29 to −0.10; P < 0.001), corresponding to 50.0% within this specified model. A separate serum-path model included 45 complete paired cases (**Figure 8c**) and yielded an estimated indirect association through T2 sTREM2/IL-6 of β = −0.06 (95% CI, −0.11 to −0.02; P = 0.004), corresponding to 15.8% within that model. Because the two estimates arose from different complete-case subsets and different model specifications, they were displayed separately and were not combined into a single 100% decomposition. The tissue-path indirect estimate remained statistically supported until approximately ρ = 0.26 in the residual-confounding sensitivity analysis (**Figure 8d**).

**Figure 8:**
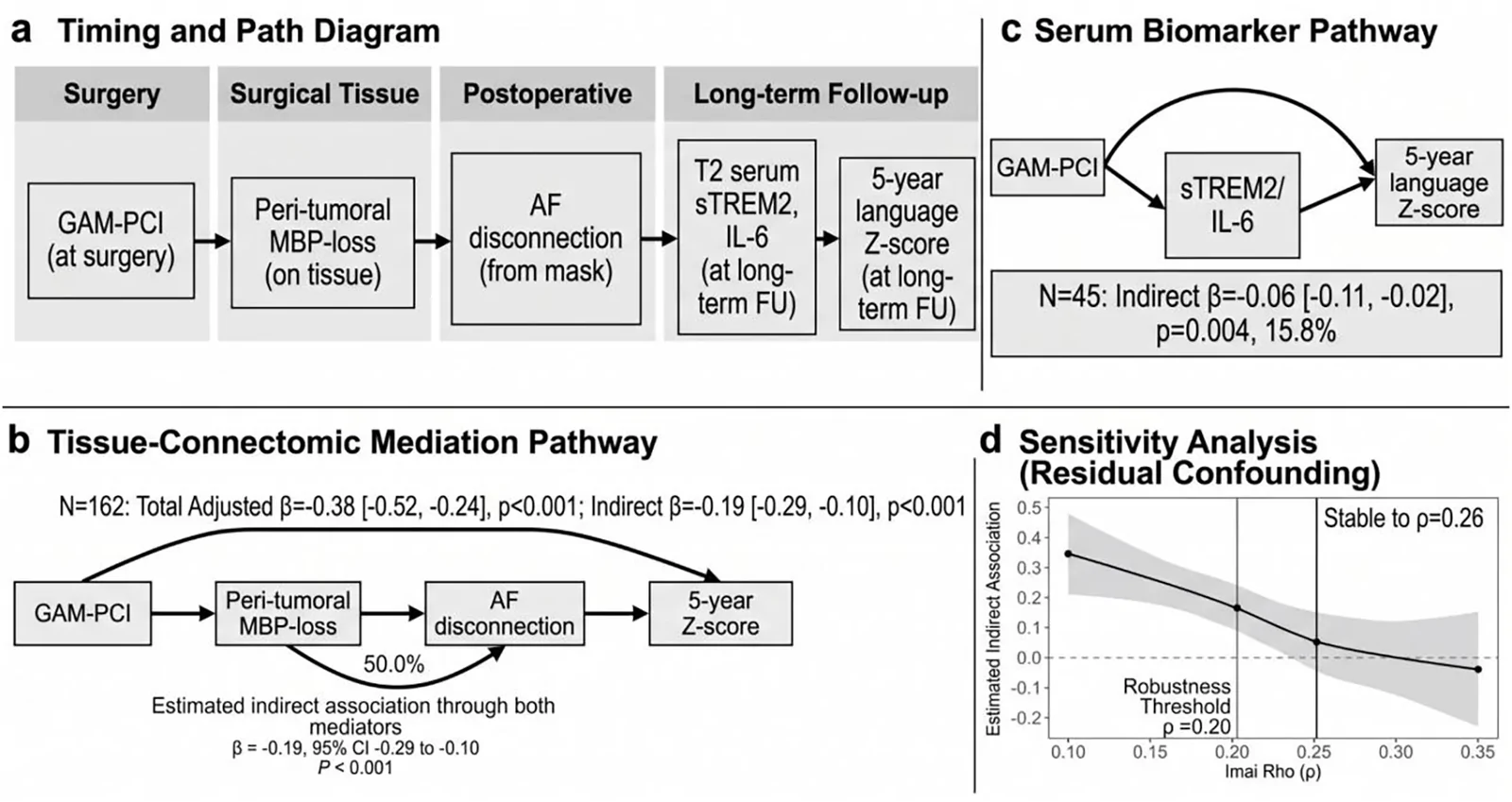
Observational sequential mediation analysis under prespecified temporal-ordering and confounding assumptions. **(a)** Timing-aware hypothesized path structure. GAM-PCI and peri-tumoral MBP loss were measured in surgical tissue; AF disconnection was derived from the postoperative lesion mask; T2 sTREM2, IL-6, and the Language Z-score were assessed at long-term follow-up. Arrows represent the prespecified model structure rather than established causal direction. **(b)** Tissue-connectomic complete-case model (n = 162): total adjusted association β = −0.38 per SD (95% bootstrap CI, −0.52 to −0.24; P < 0.001) and estimated serial indirect association through MBP loss and AF disconnection β = −0.19 (95% CI, −0.29 to −0.10; P < 0.001), corresponding to 50.0% within this model. **(c)** Separate serum-path model (n = 45): estimated indirect association through T2 sTREM2/IL-6 β = −0.06 (95% CI, −0.11 to −0.02; P = 0.004), corresponding to 15.8% within that model. **(d)** Imai ρ sensitivity analysis for residual mediator–outcome confounding; the tissue-path indirect estimate remained statistically supported until approximately ρ = 0.26, exceeding the prespecified ρ = 0.20 threshold. The two models used different complete-case subsets and were not combined into a single additive decomposition. AF, arcuate fasciculus; CI, confidence interval; GAM-PCI, glioma-associated myeloid polarization continuum index; IL-6, interleukin-6; MBP, myelin basic protein; sTREM2, soluble triggering receptor expressed on myeloid cells 2.

### 3.11 IDH mutation-stratified analysis

Pre-specified IDH-stratified analyses are summarized in **Table 6** and **Figure 9**. Among tissue-available patients, peri-tumoral CD68+ density was 58.6 cells/HPF in IDH-mutant tumors and 92.4 cells/HPF in IDH-wildtype tumors; Iba1+ density was 54.3 and 85.5 cells/HPF, respectively (both P < 0.001; **Figure 9a**). GAM-PCI was lower in IDH-mutant than IDH-wildtype tumors (median, −0.42 vs. +0.31; P < 0.001; **Figure 9b**). C4 proportion was 16.8% versus 18.1% (difference, +1.3 percentage points; 95% CI, −3.8 to +6.4; P = 0.62; **Figure 9c**). The Hodges–Lehmann shift was +1.2 percentage points (95% CI, −3.6 to +6.1), and equivalence within a ±5-percentage-point margin was not established (90% CI, −2.98 to +5.58; TOST P = 0.078). AF disconnection was associated with long-term cognitive impairment in the overall cohort (adjusted odds ratio, 1.52 per 10% increase; 95% CI, 1.25–1.88; P < 0.001) and in the IDH-mutant subgroup (1.68; 95% CI, 1.32–2.15; P < 0.001), but not in the IDH-wildtype subgroup (1.28; 95% CI, 0.88–1.85; P = 0.19); the AF × IDH interaction was P = 0.032 (**Figure 9d**). The Language trajectory interaction was significant in IDH-mutant patients (P < 0.001; T1→T2 recovery d = 1.28 for AF-intact and 0.15 for AF-disconnected; **Figure 9e**) but not in IDH-wildtype patients (P = 0.12; **Figure 9f**).

**Table 6:** Multivariable logistic regression for long-term cognitive impairment, stratified by IDH mutation status.

Predictor variable	IDH-mutant aOR (95% CI) (n=168)	P	IDH-wildtype aOR (95% CI) (n=46)	P	P (Interaction) c	VIF
Demographic and clinical						
Age, per 5-year increase	1.28 (1.08–1.52)	0.004	1.15 (0.82–1.62)	0.42	0.38	1.12
Education, per year increase	0.84 (0.76–0.93)	<0.001	0.92 (0.78–1.08)	0.31	0.22	1.18
RT dose > 54 Gy	2.15 (1.08–4.28)	0.030	1.58 (0.62–4.05)	0.34	0.45	1.24
Connectomic disconnection						
AF disconnection, per 10% a	1.68 (1.32–2.15)	<0.001	1.28 (0.88–1.85)	0.19	0.032	1.85
IFOF disconnection, per 10% a	1.35 (1.08–1.68)	0.008	1.18 (0.82–1.70)	0.38	0.28	1.92
Immune microenvironment (mIF-derived)						
GAM-PCI, per SD increase b	1.42 (1.12–1.82)	0.004	1.28 (0.82–1.98)	0.28	0.38	2.18
FlowSOM C4 (TREM2^hi^), per 10%	1.32 (1.08–1.62)	0.008	1.22 (0.85–1.75)	0.28	0.42	1.92
Peri-WM MBP-loss index, per 0.1	1.28 (1.08–1.52)	0.004	1.18 (0.82–1.70)	0.38	0.48	2.12
M2/M1 ratio (legacy)	1.18 (0.85–1.65)	0.32	1.12 (0.62–2.05)	0.68	0.78	2.38
Interaction terms						
Education × AF disconnection	β = −0.08	0.022	β = −0.03	0.45	0.048	1.52
GAM-PCI × AF disconnection	β = 0.12	0.14	β = 0.08	0.62	0.55	1.68

Binary logistic regression with global cognitive impairment (composite Z-score < −1.5 SD at T2) as the dependent variable, estimated separately within each IDH subgroup. Variables with P < 0.10 in pooled univariable analysis were entered with backward stepwise selection (likelihood ratio test). a, Connectome-based Lesion-Symptom Mapping (CLSM)-derived disconnection probability entered as continuous variable per 10% increment (primary analysis). b, GAM-PCI (Glioma-Associated Myeloid Polarization Continuum Index) is the first principal component of the FlowSOM meta-cluster proportion vector from multiplex immunofluorescence (mIF; see **Table 2**). c, P (Interaction): formal test of multiplicative interaction between the predictor and IDH status, obtained by adding the interaction term to the pooled full-cohort model. Bold P-values indicate statistical significance (P < 0.05). Key finding: AF disconnection probability was a significantly stronger predictor of long-term cognitive impairment in IDH-mutant patients (aOR 1.68, P < 0.001) compared to IDH-wildtype patients (aOR 1.28, P = 0.19), with a significant IDH interaction (P = 0.032). The Education × AF disconnection interaction was significant only in the IDH-mutant subgroup (β = −0.08, P = 0.022), indicating that cognitive reserve partially buffers the impact of white matter disconnection in this molecularly defined subtype. aOR, adjusted odds ratio; CI, confidence interval; AF, arcuate fasciculus; IFOF, inferior fronto-occipital fasciculus; RT, radiotherapy; mIF, multiplex immunofluorescence; DAM, disease-associated microglia; MBP, myelin basic protein; WM, white matter; M2/M1 ratio, CD163^+^/CD68^+^ ratio; VIF, variance inflation factor; SD, standard deviation.

**Figure 9:**
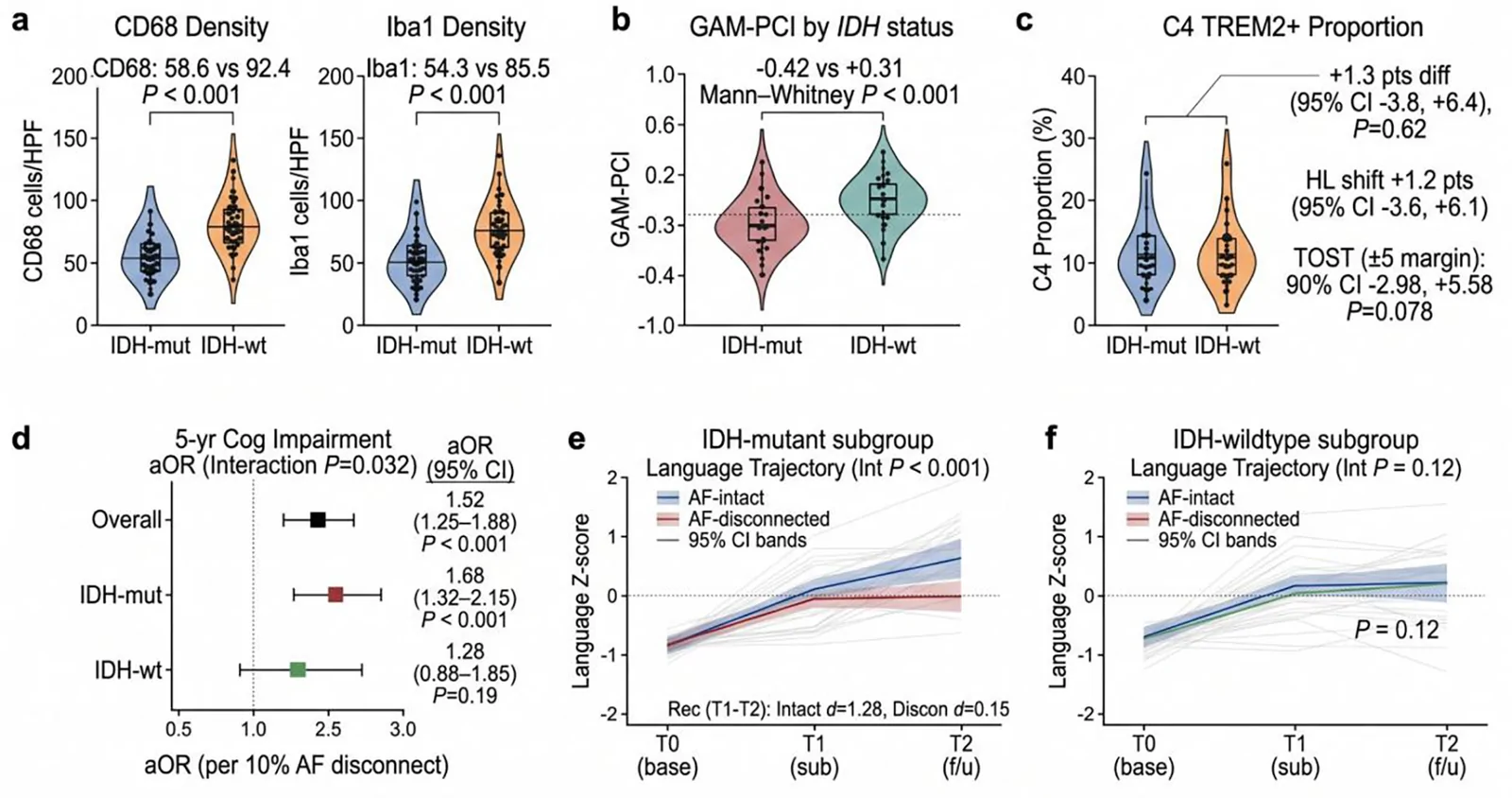
IDH mutation–stratified myeloid and connectomic associations with long-term language outcome. **(a)** Patient-level peri-tumoral CD68+ and Iba1+ densities in tissue-available IDH-mutant and IDH-wildtype tumors. CD68+ density was 58.6 versus 92.4 cells/HPF, and Iba1+ density was 54.3 versus 85.5 cells/HPF (both P < 0.001). **(b)** GAM-PCI by IDH status (median, −0.42 vs. +0.31; Mann–Whitney P < 0.001). **(c)** C4 TREM2-high proportion by IDH status (16.8% vs. 18.1%; difference, +1.3 percentage points; 95% CI, −3.8 to +6.4; P = 0.62). The Hodges–Lehmann shift was +1.2 percentage points (95% CI, −3.6 to +6.1), and equivalence within ±5 percentage points was not established (90% CI, −2.98 to +5.58; TOST P = 0.078). **(d)** Adjusted odds ratios for 5-year cognitive impairment per 10% AF disconnection increase: overall, 1.52 (95% CI, 1.25–1.88); IDH-mutant, 1.68 (1.32–2.15); IDH-wildtype, 1.28 (0.88–1.85); AF × IDH interaction P = 0.032. **(e)** Language trajectories in IDH-mutant patients by AF status (Time × AF P < 0.001; T1→T2 recovery d = 1.28 for AF-intact and 0.15 for AF-disconnected). **(f)** Corresponding IDH-wildtype trajectories (Time × AF P = 0.12). Lack of a detectable C4 difference is not evidence of IDH independence. AF, arcuate fasciculus; aOR, adjusted odds ratio; CI, confidence interval; GAM-PCI, glioma-associated myeloid polarization continuum index; HPF, high-power field; IDH, isocitrate dehydrogenase; TOST, two one-sided tests.

The three-way Education × AF × time interaction was significant only in IDH-mutant patients (P = 0.015), consistent with an evolving cognitive-reserve buffering effect across the 5-year window. At the cross-sectional level, the Education × AF disconnection interaction was significant only in the IDH-mutant subgroup (β = −0.08, P = 0.022), where the three education tertile regression lines showed a divergent fan pattern of predicted 5-year Language Z-scores as AF disconnection probability increased, with low-education (<12 y) patients crossing below the clinical impairment threshold (Z = −1.5) near 60% disconnection while high-education (≥16 y) patients remained above threshold even at 100% disconnection (**Supplementary Figure S4a**). In the IDH-wildtype subgroup, the three education tertile lines ran approximately parallel across the full range of disconnection (β = −0.03, P = 0.45; **Supplementary Figure S4b**), indicating that cognitive reserve acts as an effective buffer only when overall disease burden does not overwhelm it.

### 3.12 Multimodal prediction of 5-year cognitive outcomes

#### 3.12.1 Primary clinical–connectomic nomogram

The final multivariable logistic model for 5-year global cognitive impairment included five predictors (**Table 7**; **Figure 10a**): age per 5 years (adjusted odds ratio, 1.24; 95% CI, 1.08–1.45; P = 0.003), education per year (0.86; 95% CI, 0.79–0.94; P < 0.001), radiotherapy >54 Gy (1.92; 95% CI, 1.04–3.68; P = 0.019), AF disconnection per 10% increase (1.52; 95% CI, 1.25–1.88; P < 0.001), and IFOF disconnection per 10% increase (1.28; 95% CI, 1.05–1.56; P = 0.014). The corresponding risk nomogram is shown in **Figure 10b**. With 88 events and five retained predictors, the final-model events-per-variable ratio was 17.6; the previously reported value of 14.7 referred to six candidate predictors before final retention. Discrimination is shown in **Figure 10c**: apparent AUC/C-index was 0.82 (95% CI, 0.78–0.86), the optimism-corrected C-index after 1,000 bootstrap resamples was 0.79, and repeated 10-fold cross-validation yielded a mean AUC of 0.81 (SD, 0.04). **Figure 10d** presents apparent and bootstrap bias-corrected calibration; the Hosmer–Lemeshow P value was 0.42. **Figure 10e** shows decision-curve analysis, with the model providing greater net benefit than treat-all or treat-none over the prespecified 15–85% threshold range. These are internal-validation results and do not constitute external validation.

**Table 7:** Multivariable logistic regression analysis identifying predictors of long-term global cognitive impairment (full cohort, N = 214), with sensitivity analysis.

Predictor variable	Univariable OR (95% CI)	P	Multivariable aOR (95% CI)	P	VIF
Demographic and clinical					
Age at diagnosis, per 5-year increase	1.32 (1.15–1.54)	<0.001	1.24 (1.08–1.45)	0.003	1.15
Education, per year increase	0.82 (0.75–0.89)	<0.001	0.86 (0.79–0.94)	<0.001	1.22
KPS score (preoperative)	0.96 (0.92–1.01)	0.12	—	—	—
Tumor and molecular					
Tumor volume, per 10 cm³	1.18 (1.05–1.34)	0.006	1.05 (0.92–1.21)	0.45 †	1.85
IDH status (wildtype vs mutant)	2.15 (1.12–4.15)	0.021	1.45 (0.75–2.85)	0.26	1.42
WHO grade (4 vs 2/3)	1.95 (1.05–3.65)	0.035	—	—	—
Treatment					
Adjuvant radiotherapy > 54 Gy	1.85 (1.02–3.45)	0.042	1.92 (1.04–3.68)	0.019	1.18
Extent of resection (STR vs GTR)	1.45 (0.85–2.45)	0.18	—	—	—
Connectomic disconnection (CLSM, per 10% increment)					
Left AF disconnection	1.65 (1.35–2.05)	<0.001	1.52 (1.25–1.88)	<0.001	2.15
Left IFOF disconnection	1.42 (1.18–1.72)	<0.001	1.28 (1.05–1.56)	0.014	2.08
Left UF disconnection	1.15 (0.95–1.38)	0.15	—	—	—
Anterior thalamic radiation	1.25 (1.02–1.54)	0.032	1.10 (0.88–1.35)	0.38	1.65
Sensitivity analysis: primary aOR for left AF disconnection (per 10%)					
Complete-case (primary)	—	—	1.52 (1.25–1.88)	<0.001	—
Multiple imputation (MICE, m = 40)	—	—	1.53 (1.27–1.85)	<0.001	—
Inverse probability-weighted (IPW)	—	—	1.49 (1.22–1.82)	<0.001	—
Second-reader segmentation masks (n = 20)	—	—	1.49 (1.21–1.85)	<0.001	—
Threshold = 0.40 (dichotomized)	—	—	1.42 (1.18–1.72)	<0.001	—
Threshold = 0.60 (dichotomized)	—	—	1.61 (1.28–2.02)	<0.001	—
E-value (point estimate/lower CI)	—	—	1.77/1.48	—	—

Binary logistic regression used global cognitive impairment (composite Z-score < −1.5 SD at T2) as the dependent variable in the full cohort (N = 214; 88 events). The final model retained five predictors, giving a final-model EPV of 17.6; the ratio of 14.7 refers to six candidate predictors before final retention. Model performance was C-index 0.82 (95% CI, 0.78–0.86), optimism-corrected C-index 0.79, Hosmer–Lemeshow P = 0.42, and repeated 10-fold cross-validated AUC 0.81 (SD, 0.04). The IPW sensitivity analysis used stabilized inverse-probability-of-inclusion weights estimated in the 466-patient source population (214 long-term cohort participants and 252 patients who died or were not observed before 3 years), with percentile-based weight truncation and no trimming. AF, arcuate fasciculus; AUC, area under the receiver operating characteristic curve; CI, confidence interval; CLSM, connectome-based lesion-symptom mapping; EPV, events per variable; IDH, isocitrate dehydrogenase; IFOF, inferior fronto-occipital fasciculus; IPW, inverse probability weighting; MICE, multiple imputation by chained equations; OR, odds ratio; VIF, variance inflation factor.

**Figure 10:**
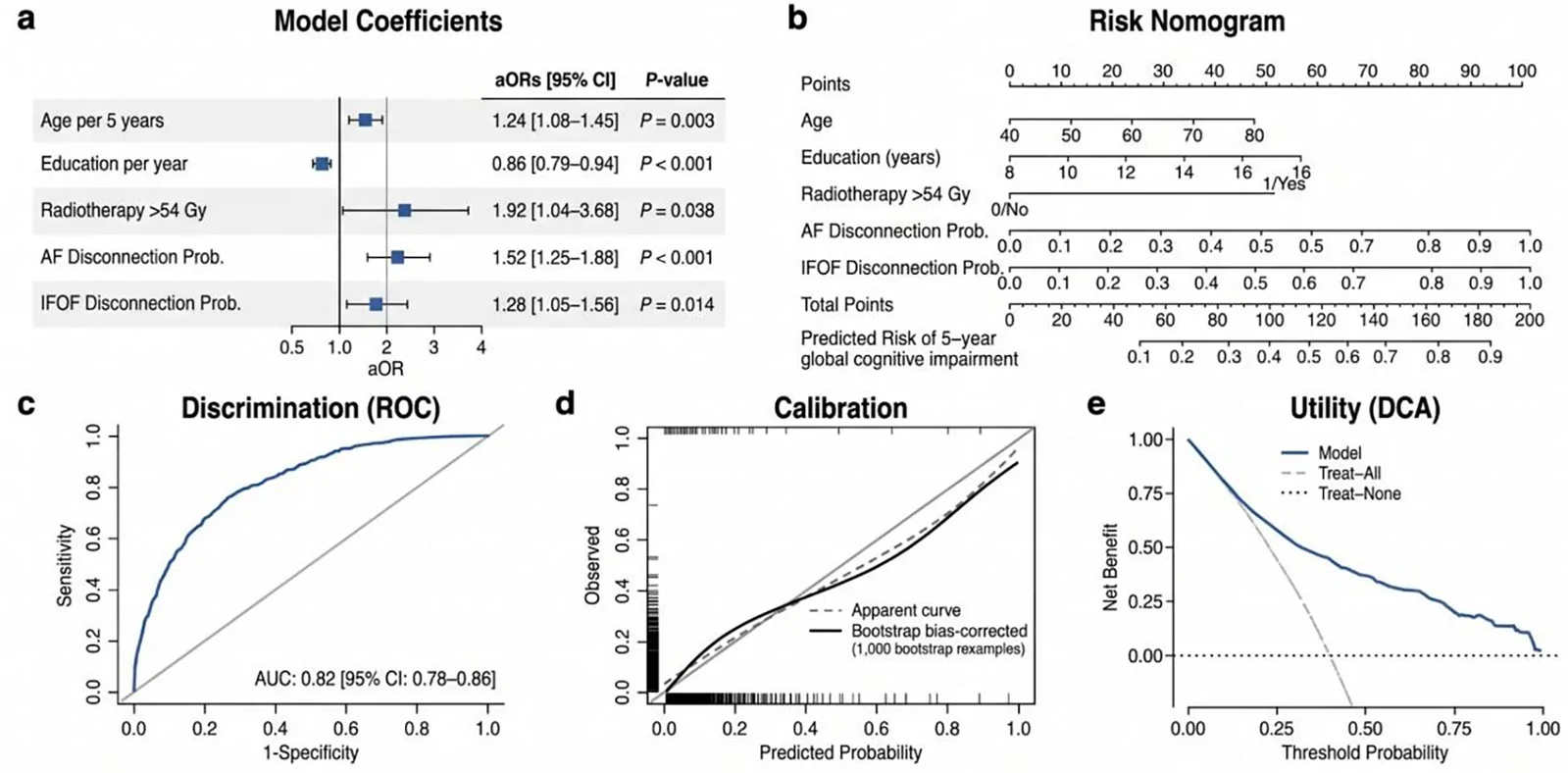
Clinical–connectomic prognostic model for 5-year global cognitive impairment with internal validation. **(a)** Forest plot of the five retained predictors in the final model (N = 214; 88 events): age per 5 years, aOR 1.24 (95% CI, 1.08–1.45; P = 0.003); education per year, 0.86 (0.79–0.94; P < 0.001); radiotherapy >54 Gy, 1.92 (1.04–3.68; P = 0.019); AF disconnection per 10% increase, 1.52 (1.25–1.88; P < 0.001); and IFOF disconnection per 10% increase, 1.28 (1.05–1.56; P = 0.014). Final-model EPV was 17.6. **(b)** Risk nomogram generated from the final fitted model. **(c)** ROC analysis: apparent AUC/C-index 0.82 (95% CI, 0.78–0.86), optimism-corrected C-index 0.79, and repeated 10-fold cross-validated AUC 0.81 (SD, 0.04). **(d)** Apparent and bootstrap bias-corrected calibration after 1,000 resamples; Hosmer–Lemeshow P = 0.42. **(e)** Decision-curve analysis versus treat-all and treat-none over threshold probabilities of 0.15–0.85. All performance estimates are internal-validation results. AF, arcuate fasciculus; aOR, adjusted odds ratio; AUC, area under the receiver operating characteristic curve; CI, confidence interval; DCA, decision-curve analysis; EPV, events per variable; IFOF, inferior fronto-occipital fasciculus; ROC, receiver operating characteristic.

#### 3.12.2 Exploratory biomarker-augmented nomogram

An extended nomogram incorporating T2 serum NfL and T2 IL-6 alongside the clinical and connectomic predictors was developed in the biomarker sub-cohort (n = 72; **Supplementary Figure S5a**: extended nomogram structure). Because this sub-cohort has an events-per-variable ratio of approximately 4.3 (**Supplementary Table S4**, Model 3 column, EPV = 4.3), below the conventional threshold of 10, the extended model was designated *a priori* as exploratory and hypothesis-generating. Nested model comparison (**Supplementary Table S3**) demonstrated that Model 2 (clinical + CLSM + NfL) provided only marginal improvement over Model 1 (AUC 0.82 → 0.84; DeLong P = 0.065; NRI non-significant), whereas Model 3 (clinical + CLSM + NfL + IL-6) provided statistically significant incremental value: AUC improved from 0.82 to 0.86 (ΔAUC = +0.04, DeLong P = 0.028), NRI = 0.12 (95% CI 0.03–0.21, P = 0.009), IDI = 0.032 (95% CI 0.008–0.056, P = 0.012), and AIC decreased from 68.4 to 62.1 (likelihood ratio test P = 0.008). Calibration curves tracked the ideal diagonal closely for both models (**Supplementary Figure S5b**: base model C = 0.82 vs. extended model C = 0.86, ΔC = +0.04). Decision curve analysis showed that the extended model provided incremental net benefit specifically in the 30–60% threshold probability range — the clinical decision zone of greatest uncertainty — while tracking the base model closely at lower and higher thresholds (**Supplementary Figure S5c**). A prospective validation cohort of n ≥ 280 with ≥115 events would be required to validate the extended nomogram at C-index 0.84 with ±0.05 precision. No clinical recommendation is made on the basis of this exploratory model.

### 3.13 Sensitivity analysis confirm robustness of primary findings

#### 3.13.1 AF disconnection threshold sensitivity

Across six candidate binarization thresholds from ≥0.30 to ≥0.80, the Time × Lesion Status interaction coefficient from the linear mixed-effects model for the Language domain ranged from 0.95 to 1.48 (all P < 0.001), and the AUC of binary AF status as a single predictor of LTCD ranged from 0.68 to 0.79 (**Supplementary Table S8**). The threshold of ≥0.50 was empirically selected by maximizing the Youden Index (Sensitivity + Specificity − 1 = 0.50) from the ROC curve predicting LTCD, yielding Sensitivity 0.72, Specificity 0.78, and AUC 0.79 (95% CI 0.73–0.85; **Supplementary Figure S6**: ROC curve with all six threshold operating points annotated; the selected ≥0.50 point is indicated with an enlarged crimson marker at the Youden-optimal position). Lower thresholds (≥0.30, ≥0.40) increased sensitivity (0.82 and 0.78) but substantially reduced specificity (0.58 and 0.68), while higher thresholds (≥0.70, ≥0.80) yielded diminishing AUC (0.74 and 0.68) due to reduced sample size in the Disconnected group (n = 42 and n = 28, respectively). The continuous-variable analysis (primary; AF disconnection per 10% increment) yielded an adjusted odds ratio of 1.52, consistent with the finding that the main cognitive–connectomic relationship does not depend on any specific threshold. Restricted cubic spline modeling (4 knots at the 5th, 35th, 65th, and 95th percentiles) identified a statistically significant non-linear relationship (likelihood ratio test P = 0.04 versus linear model), with a plateau-and-rise pattern inflecting near AF disconnection probability 0.45.

#### 3.13.2 Multiple imputation sensitivity analysis

Little’s MCAR test rejected MCAR for the cognitive matrix (P < 0.001) but not for the biomarker matrix (P = 0.21), consistent with attrition-driven missingness in cognitive data. Multiple imputation by chained equations with m = 40 imputations was applied to the analysis dataset, incorporating auxiliary variables (MGMT methylation, 1p/19q status, baseline Hospital Anxiety and Depression Scale scores, marital status). The imputation-pooled AF disconnection adjusted odds ratio for the primary endpoint was 1.53 (95% CI 1.27–1.85), virtually identical to the complete-case estimate of 1.52 (95% CI 1.25–1.88), satisfying the pre-specified concordance criterion. All other primary predictors retained their direction and significance under multiple imputation.

#### 3.13.3 Loss-to-follow-up tipping-point and E-value

The pattern-mixture tipping-point analysis for the 252 patients who died within 3 years showed that the primary AF-disconnection adjusted odds ratio remained statistically significant (lower 95% CI > 1.0) until at least 70% of these patients were assigned cognitively preserved outcomes in the simulated scenarios. Because long-term cognitive decline occurred in 41.1% of the cohort, the odds ratio was converted using RR ≈ √OR before calculation of the E-value. The E-value was 1.77 for the point estimate and 1.48 for the lower 95% confidence limit.

#### 3.13.4 Secondary hypothesis testing under hierarchical multiplicity control

Under the prespecified hierarchical testing strategy, the primary gatekeeper hypothesis (AF disconnection predicts the primary endpoint) was supported at P < 0.001, permitting formal testing of the secondary hypotheses. The secondary analysis supported associations between GAM-PCI and AF disconnection (P < 0.001), a model-based indirect association involving peri-tumoral MBP loss and AF disconnection (P < 0.001), and higher T2 sTREM2 and CHI3L1 concentrations in AF-disconnected patients (P = 0.003 and P < 0.001, respectively). Benjamini–Hochberg FDR correction at q = 0.05 did not alter the significance classification of the primary or secondary findings.

## 4 Discussion

In this single-center retrospective cohort of 214 adults with WHO grade 2–4 diffuse glioma followed for a median of 5.2 years, long-term cognitive decline occurred in 41.1%. Three findings support an integrated neuroimmune-connectomic association framework. First, AF disconnection independently predicted long-term global cognitive impairment. Second, FlowSOM analysis of 2.41 × 10^6^ CD68+/Iba1+ cells from 186 patients resolved six stable marker-defined myeloid states, including a peri-tumoral TREM2-high DAM-like protein phenotype. Third, the 162-patient tissue-connectomic model and the separate 45-patient serum-path model yielded indirect-association estimates consistent with the prespecified pathways. Because temporal ordering was assumed and the two models used different complete-case subsets, these findings are structural decompositions rather than causal effects or components of a single additive mechanism.

The observation that AF disconnection outperformed tumor volume, IDH status, and extent of resection in predicting cognitive outcome aligns with the conceptualization of glioma as a network disorder rather than a strictly focal lesion (25). The magnitude of effect (adjusted odds ratio 1.52 per 10% increment) is consistent with post-stroke disconnectome models in which structural disconnection outperforms lesion volume for predicting sensorimotor and language deficits (26). The anatomical dissociation between transient deficits (localized to cortical gray matter and superficial U-fibers) and permanent deficits (confined to deep white matter bottlenecks) provides a structural explanation for divergent “V-shaped” and “L-shaped” cognitive trajectories: damage to cortical hubs permits perilesional or contralateral compensation, whereas damage to deep bottleneck tracts prevents efficient rerouting of information flow (27). Restricted cubic spline analysis (likelihood ratio test P = 0.04 versus linear model) identified a plateau-and-rise inflection near AF disconnection probability 0.45, empirical support for a biologically meaningful but not threshold-specific transition beyond which compensation becomes limited. The concordance between normative Disconnectome-derived probabilities and patient-specific DTI-derived fractional anisotropy (Spearman ρ = −0.72 for AF; ρ = −0.61 for UF) supports the reliability of the normative approach for clinical workflows lacking routine individual diffusion acquisition (28).

The identification of six myeloid meta-clusters (bootstrap Jaccard 0.78–0.88), including a 20.6% CD163hi iNOShi dual-high transitional population, shows that the dichotomous M1/M2 framework that has dominated glioma immunology does not adequately describe these data. These findings converge with single-cell and CITE-seq data from Pombo Antunes et al. (29) and Ochocka et al. (23), and with the mass-cytometry interrogation of brain tumor microenvironments describing continuous myeloid landscapes resistant to binary classification (30). The application of high-dimensional unsupervised clustering to protein-level multiplex immunofluorescence data extends to human glioma the approach previously used to resolve microglial heterogeneity in non-neoplastic human brain (31). Per-patient GAM-PCI captured 47.2% of variance in myeloid composition and correlated with the legacy CD163^+^/CD68^+^ ratio (ρ = 0.78), but outperformed it in association with AF disconnection (R = 0.45 vs. R = 0.38), indicating that continuum representation captures prognostically relevant information lost in dichotomous reduction. The enrichment of CD163/CD206-high (C3) and TREM2-high (C4) myeloid cells at the peri-tumoral white matter zone relative to the tumor core (C3: 26.9% vs. 17.6%; C4: 21.0% vs. 13.8%; both paired Wilcoxon P < 0.001), together with the spatial colocalization of CD163^+^CD206^+^ cells with MBP^+^ myelin fragments in that zone (Morisita–Horn index 0.71) and their proximity to the white matter boundary (mean nearest-neighbor distance 38 ± 14 µm vs. null 87 µm, P < 0.001) (32), provides spatial evidence that M2/TREM2-skewed myeloid cells are positioned within the compartment in which peri-tumoral myelin injury occurs. We note that the colocalization and distance statistics were computed on CD163^+^CD206^+^ cells rather than on the TREM2-high cluster, and that the distance measured is to the white matter boundary rather than to myelin fragments (33, 34).

The TREM2-high C4 state has a protein phenotype compatible with a DAM-like program, but the designation rests on TREM2 protein expression and external transcriptomic triangulation rather than molecular validation in the same specimens (35–37). In 45 patients with paired measurements, T2 serum sTREM2 was associated with C4 proportion (ρ = 0.42, P = 0.004), but sTREM2 is not DAM-specific and the analysis does not establish its cellular source. C4 proportion did not differ detectably by IDH status, and equivalence was not established. These qualifications limit inference about lineage, program identity, and cross-compartment origin.

Confirming that C4 corresponds to the DAM program in these tumours requires single-cell or spatial transcriptomic profiling of the same specimens, which we have not performed. And because the panel resolves activation state rather than ontogeny, we cannot determine whether the cells constituting C4 are resident microglia that have acquired a TREM2-high state or infiltrating monocyte-derived macrophages. Adding P2RY12 and TMEM119 to the panel would improve, but would not resolve, this question: both markers are progressively downregulated as microglia acquire the DAM/MGnD program and both are lost at chronic active lesion borders and in peri-lesional white matter, so a P2RY12-low cell at the glioma–brain interface remains ambiguous between an infiltrating monocyte-derived macrophage and a state-transitioned resident microglial cell (38). Our own regional data are consistent with this: the P2RY12^+^ fraction of Iba1^+^ cells falls from 54.2% at the infiltrative margin to 22.4% in the tumour core, and correlates negatively with the TREM2-high compartment at the patient level. Definitive ontogenetic assignment in human tissue requires lineage-informative approaches unavailable in an archival formalin-fixed cohort — chimerism after sex-mismatched transplantation, or single-cell transcriptomic or epigenomic profiling of fresh tissue (39, 40). The limitation is therefore a constraint on the class of inference available from any protein panel in this setting, not a deficiency of a particular antibody selection. In the 162-patient tissue-connectomic model, the estimated serial indirect association was β = −0.19 (95% CI, −0.29 to −0.10; P < 0.001), corresponding to 50.0% within that model. In the separate 45-patient serum-path model, the estimated indirect association was β = −0.06 (95% CI, −0.11 to −0.02; P = 0.004), corresponding to 15.8% within that model. The tissue-path sensitivity analysis remained statistically supported until approximately ρ = 0.26. These estimates were not combined because they were derived from different complete-case subsets and model specifications (41). The attenuation pattern in the joint regression was consistent with, but did not prove, the prespecified tissue-connectomic ordering.

The AF × IDH interaction may have translational relevance because the association between AF disconnection and cognitive outcome was stronger in IDH-mutant survivors than in the smaller IDH-wildtype subgroup. However, the IDH-wildtype estimate was imprecise, and the study was not designed to establish equivalence of the C4 state across molecular subtypes (42, 43). These subgroup findings require external validation and should not be interpreted as evidence that C4 is biologically IDH-independent. The longitudinal serum panel demonstrated persistent differences between the AF-disconnected and AF-intact groups, including higher T2 concentrations of NfL, IL-6, sTREM2, and CHI3L1 in patients with AF disconnection. In the adjusted T2 linear regression analysis, higher log10-transformed IL-6 was associated with lower Language, Memory, and Global Composite Z-scores. The estimated association was largest for the language domain (β = −0.24, SE = 0.100, 95% CI −0.44 to −0.04; P = 0.019), although the confidence interval remained relatively wide. The Spearman analysis also showed that IL-6 was correlated with AF disconnection probability (ρ = 0.35) and inversely correlated with the Language Z-score (ρ = −0.24). Because the biomarker and cognitive measurements were obtained at the same long-term follow-up assessment, these results cannot establish whether elevated IL-6 preceded cognitive dysfunction, resulted from structural or treatment-related injury, or represented a parallel systemic response (44–47).

The association of a peri-tumoral TREM2-high state with myelin injury and cognitive outcome motivates mechanistic evaluation of the TREM2 axis in glioma, but the present data do not demonstrate that C4 has tumor-promoting or tissue-protective functions, nor do they establish that systemic TREM2 blockade would improve or worsen cognition. Any therapeutic study should therefore assess tumor response, cognitive outcomes, and longitudinal connectomic measures and should distinguish tumor-core from peri-tumoral myeloid effects. The paired serum–tissue association (n = 45; ρ = 0.42, P = 0.004) supports prospective evaluation of sTREM2 as a candidate monitoring biomarker. It does not validate sTREM2 for pharmacodynamic use, establish specificity for C4, or identify the cellular source of circulating sTREM2.

### 4.1 Relation to the layered spatial organization of glioma

Our findings should be read against the spatial architecture of glioma described by Greenwald et al., who combined spatial transcriptomics of 19 glioma sections with 13 published glioblastoma sections and spatial proteomics of 12 samples, and found that structured tumor regions are arranged in five layers ordered by a hypoxia gradient: a hypoxic mesenchymal core; a hypoxia-adjacent layer containing mesenchymal malignant states together with an inflammatory macrophage program; an immune and angiogenic layer; a layer of neurodevelopmental malignant states; and, outermost, the infiltrated brain parenchyma of oligodendrocytes, neurons and reactive astrocytes (48). The harmonized GBmap atlas independently recovers hypoxia-dependent niches as a dominant axis of glioblastoma spatial organization and relates them to prognosis (20), and earlier spatially resolved multi-omic work likewise found hypoxic and inflammatory neighborhoods to be spatially segregated from neurodevelopmental ones (34). We set out below what can and cannot be corroborated across these datasets and the present cohort, and how hypoxia bears on our results (**Supplementary Table S15**).

The level of inference available is limited in one important respect. Greenwald et al. resolve myeloid cells into two spatial metaprograms, Inflammatory-Mac and Mac; a TREM2-high or DAM-like state is not among the metaprograms they define, and their spatial proteomic panel was constructed principally around the malignant states defined by single-cell RNA sequencing. Their data therefore cannot serve as a cell-level test of our C4 assignment, and we do not present them as such. What they can be asked is whether the anatomical position we assign to the TREM2-high state is compatible with the layered architecture, and whether hypoxia — as the dominant organizer of that architecture — offers an alternative explanation for our observations.

On the first question, the peri-tumoral white matter compartment we sampled, within 5 mm of the tumor–brain interface, corresponds to the outer, hypoxia-resolved end of the gradient rather than to the peri-necrotic layers, and our data are positioned accordingly. The TREM2-high meta-cluster was enriched at the peri-tumoral interface and was at its lowest value in the tumor core (21.0% versus 13.8%; +7.2 percentage points, P < 0.001), whereas the population enriched at the infiltrative margin was the classically activated CD68hi iNOShi cluster (19.3% versus 11.2%, P = 0.001). A myeloid state generated by the hypoxia gradient would be expected to concentrate in the hypoxia-adjacent layer, immediately outside the mesenchymal-hypoxic core; the state we describe does the opposite. The hypoxia-proximal myeloid program in the layered model is also phenotypically distinct from ours, being defined by upregulated inflammatory cytokines and downregulated MHC class II rather than by TREM2 (33). A complementary observation follows from the same model: the outermost layer is the only one that contains the oligodendrocyte-lineage and astrocytic populations whose injury we measured — myelin basic protein and Olig2 in tissue, glial fibrillary acidic protein and CHI3L1 in serum (48, 49). The layered architecture therefore supplies a principled anatomical reason why the myeloid states relevant to peri-tumoral myelin integrity, and hence to cognitive survivorship, should be those positioned at the tumor–parenchyma boundary rather than those occupying the hypoxic core, which is what we observe.

On the second question, hypoxia remains a plausible upstream determinant of myeloid polarization that we did not measure, and it bears on this work in three ways. First, as a determinant of polarization within our cohort, on which our panel is silent because it contains no hypoxia marker. Two features of the cohort nevertheless constrain how much of the finding hypoxia could explain: 168 of 214 patients (78.5%) had IDH-mutant tumors, which typically lack hypoxia and are correspondingly disorganized (33), and only 35 patients (16.4%) had WHO grade 4 tumors, for which necrosis and microvascular proliferation are defining criteria; and the TREM2-high proportion did not differ detectably by IDH status (+1.3 percentage points, 95% CI −3.8 to +6.4, P = 0.62), whereas a hypoxia-organized architecture would predict a larger TREM2-high compartment and a steeper peri-tumoral gradient in the hypoxic stratum. We present this as a directional prediction that our data do not support rather than as a positive claim, since the study was not powered for it and equivalence was explicitly not established, and since hypoxia-associated organization extends beyond histologically visible necrosis (33). Second, and more consequentially, as a confounder of the in silico triangulation: SPP1 is hypoxia-responsive and both public atlases we interrogated are dominated by core resections, so part of the TREM2hi/APOEhi/SPP1hi population identified there may reflect a hypoxia-associated rather than a disease-associated-microglia program. This does not affect the primary protein-level analysis, in which C4 is defined on TREM2 alone within a panel containing no hypoxia marker, but it does weaken the triangulation step. Third, as a sampling issue, addressed in the Limitations. Direct resolution of all three points requires hypoxia markers such as carbonic anhydrase IX, GLUT1 or HIF-1α together with distance-to-necrosis measurements on the same sections, and spatially resolved transcriptomics of peri-tumoral tissue, neither of which was performed here (50–53).

### 4.2 Limitations

Several limitations should be acknowledged. First, the single-center retrospective design introduces selection bias and requires prospective multicenter validation. Second, the biomarker sub-cohort (n = 72) had an events-per-variable ratio of approximately 4.3, so the biomarker-augmented model is exploratory. Third, spatial colocalization was two-dimensional and performed on FFPE sections. Fourth, the exposure, first mediator, and disconnection measure were obtained at common or overlapping perioperative time points; temporal ordering was assumed rather than observed, reverse ordering cannot be excluded, and the mediation estimates are model-based structural decompositions rather than identified causal effects. The ρ-sensitivity analysis addresses only a restricted class of residual mediator–outcome confounding. Fifth, cross-cultural generalization of cognitive cut-points derived from a Chinese reference sample remains to be established. Sixth, the six-marker mIF panel resolves activation state but not ontogeny; resident microglia and monocyte-derived macrophages cannot be distinguished, and adjacent-section P2RY12 immunohistochemistry provides regional rather than cell-level evidence. Seventh, the DAM-like designation rests on TREM2 protein expression and external transcriptomic triangulation, without transcriptional or functional validation in the same specimens. Eighth, although the clustering solution was stable across bootstrap resampling, seeds, grid sizes, and an independent algorithm, it remains one defensible partition of a continuous phenotypic space. Ninth, tissue spatial organization and hypoxia were not directly measured. Finally, the long-term follow-up requirement introduced survivorship bias. IPW, tipping-point analysis, and comparisons with excluded patients yielded concordant estimates but cannot remove truncation by death or guarantee generalizability to unselected IDH-wildtype glioblastoma populations.

### 4.3 Conclusions

In 214 adults with WHO grade 2–4 diffuse glioma followed for a median of 5.2 years, long-term cognitive outcome was associated with critical white matter disconnection, marker-defined peri-tumoral myeloid states, and persistent systemic neuroimmune abnormalities. Separate observational mediation models were statistically consistent with tissue-connectomic and serum pathways under explicitly stated assumptions. These data do not establish causal direction, cellular origin, or treatment effects and require prospective multicenter and mechanistic validation.

## Data Availability

The datasets presented in this study can be found in online repositories. The names of the repository/repositories and accession number(s) can be found in the article/**Supplementary Material**.
