# Supplementary material for: A peri-tumoral TREM2-high myeloid state, myeloid continuum polarization, and white matter disconnection in long-term cognitive survivorship after diffuse glioma: a neuroimmune-connectomic cohort study
Source: Front Immunol. 2026 Sep 30;17:1861684. doi: 10.3389/fimmu.2026.1861684 (PMC13662105; doi:10.3389/fimmu.2026.1861684)
Supplement: Supplementary file 1 [file DataSheet1.pdf]

SUPPLEMENTARY MATERIAL

A Peri-tumoral TREM2-High Myeloid State, Myeloid Continuum Polarization, and White Matter Disconnection in Long-Term Cognitive Survivorship After Diffuse Glioma: A Neuroimmune-Connectomic Cohort Study

Zheng Zhang and Yanyu Qi

Manuscript ID: 1861684 | Frontiers in Immunology

Supplementary Figure S1. Validation of CLSM-derived disconnection probabilities against patient-specific diffusion tensor imaging.

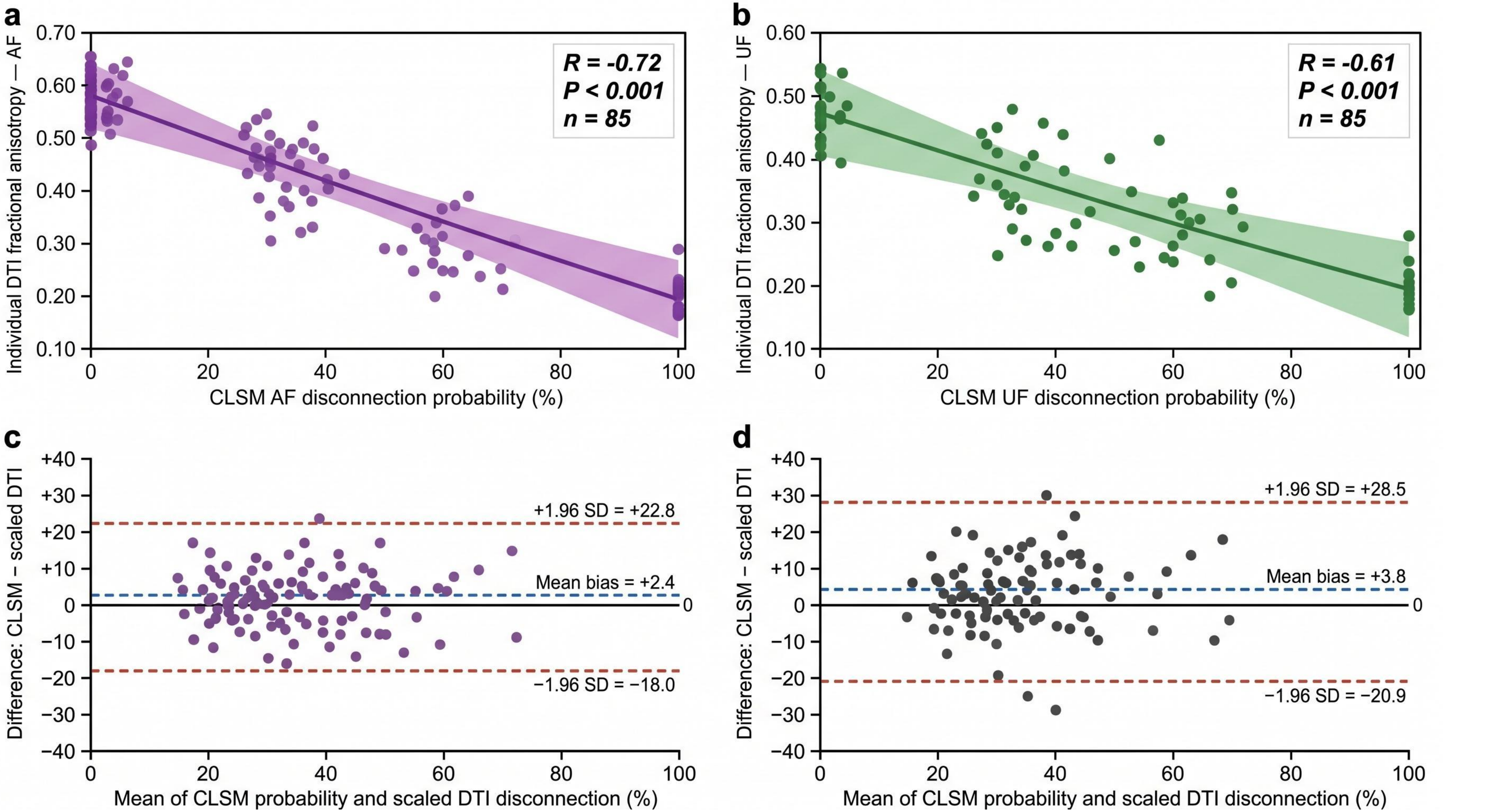

Supplementary Figure S1. Validation of normative connectome-based lesion-symptom mapping (CLSM)-derived disconnection probabilities against patient-specific diffusion tensor imaging (DTI) in the DTI validation sub-cohort (n = 85). (a) Arcuate fasciculus (AF) disconnection probability was inversely associated with individual DTI fractional anisotropy (Spearman R = -0.72, P < 0.001). (b) The corresponding association for the uncinate fasciculus (UF) was R = -0.61 (P < 0.001). (c, d) Bland-Altman plots show agreement between CLSM- and DTI-derived measures for the AF (mean bias +2.4; 95% limits of agreement, -18.0 to +22.8) and UF (mean bias +3.8; 95% limits of agreement, -20.9 to +28.5). Abbreviations: AF, arcuate fasciculus; CLSM, connectome-based lesion-symptom mapping; DTI, diffusion tensor imaging; UF, uncinate fasciculus.

Supplementary Figure S2. Associations between peri-tumoral immune markers and major white-matter tract disconnection probabilities.

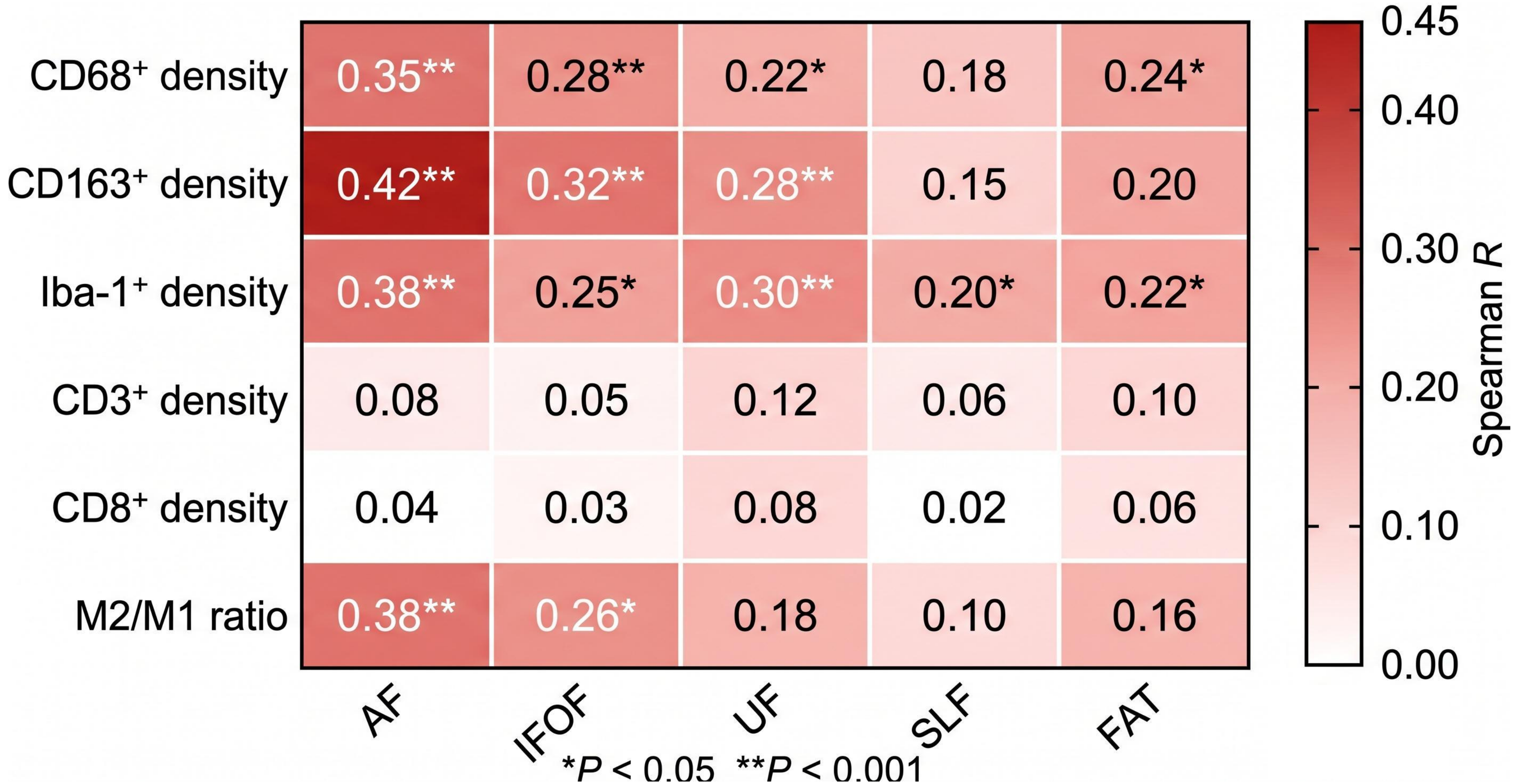

Supplementary Figure S2. Associations between peri-tumoral immune measures and major white-matter tract disconnection probabilities. Patient-level Spearman rank correlations were calculated between six peri-tumoral immune measures—CD68-positive cell density, CD163-positive cell density, Iba1-positive cell density, CD3-positive cell density, CD8-positive cell density, and the legacy CD163-positive/CD68-positive density ratio—and disconnection probabilities for the arcuate fasciculus (AF), inferior fronto-occipital fasciculus (IFOF), uncinate fasciculus (UF), superior longitudinal fasciculus (SLF), and frontal aslant tract (FAT). Cells display signed Spearman correlation coefficients. Numerical two-sided P values and pairwise-complete sample sizes are provided [within each cell/in the accompanying source-data table]. No missing values were imputed for these correlation analyses. P values are nominal and were not adjusted for multiple comparisons [or: Benjamini–Hochberg-adjusted q values are additionally reported]. The M2/M1 ratio denotes the legacy CD163-positive/CD68-positive density ratio.

Supplementary Figure S3. Longitudinal TNF-alpha and IL-1beta trajectories and their cross-sectional associations with language outcome.

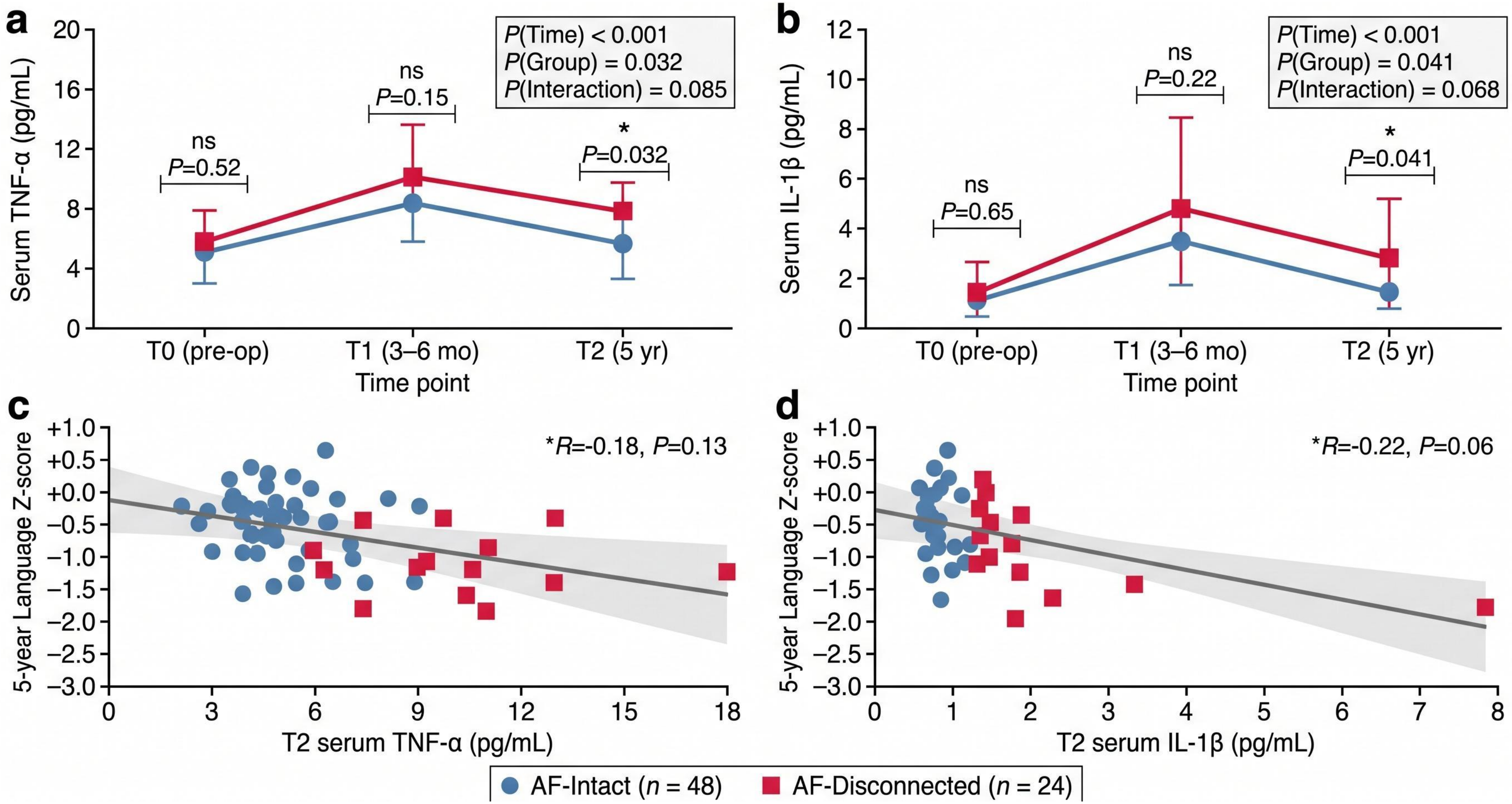

Supplementary Figure S3. Longitudinal serum tumor necrosis factor-alpha (TNF-alpha) and interleukin-1 beta (IL-1beta) in the biomarker sub-cohort (n = 72; AF-intact n = 48; AF-disconnected n = 24). (a, b) Concentrations at T0 (preoperative), T1 (3-6 months postoperative), and T2 (5-year follow-up), with the Time, Group, and Time x Group P values shown in each panel. (c, d) Cross-sectional associations of T2 TNF-alpha and IL-1beta with the 5-year Language Z-score were weak and not statistically significant (TNF-alpha: R = -0.18, P = 0.13; IL-1beta: R = -0.22, P = 0.06). Abbreviations: AF, arcuate fasciculus; IL-1beta, interleukin-1 beta; T0, preoperative baseline; T1, subacute postoperative assessment; T2, long-term follow-up; TNF-alpha, tumor necrosis factor-alpha.

Supplementary Figure S4. Education-by-AF-disconnection associations with predicted 5-year language outcome by IDH status.

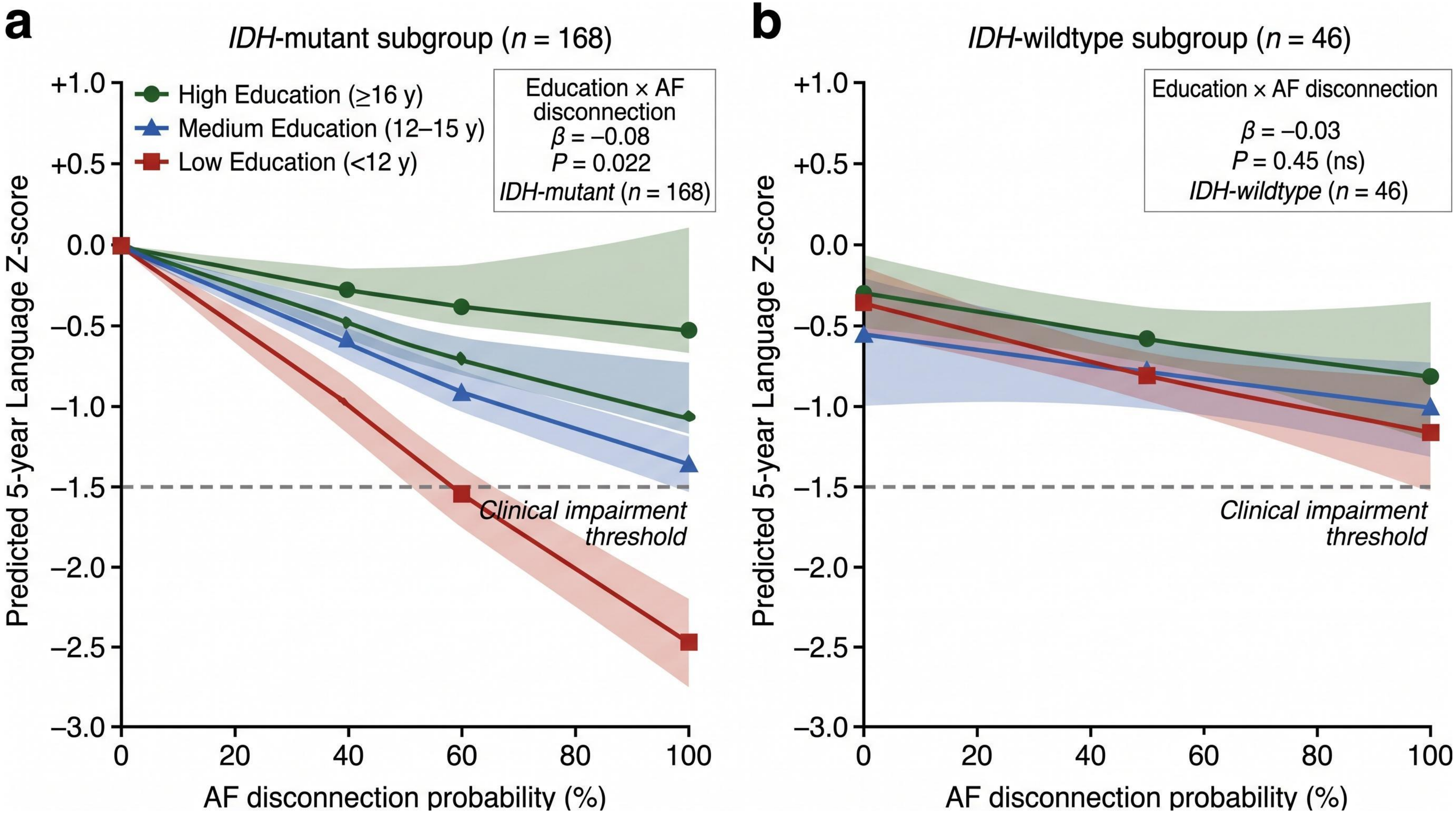

Supplementary Figure S4. Predicted 5-year Language Z-score as a function of arcuate fasciculus (AF) disconnection probability and educational attainment, stratified by isocitrate dehydrogenase (IDH) mutation status. (a) In the IDH-mutant subgroup (n = 168), the Education x AF-disconnection interaction was statistically significant (beta = -0.08, P = 0.022). (b) In the IDH-wildtype subgroup (n = 46), the interaction was not statistically significant (beta = -0.03, P = 0.45). Education groups were high (>=16 years), medium (12-15 years), and low (<12 years). The dashed horizontal line denotes the clinical impairment threshold (Z = -1.5). Abbreviations: AF, arcuate fasciculus; IDH, isocitrate dehydrogenase.

Supplementary Figure S5. Exploratory biomarker-augmented prognostic model in the biomarker sub-cohort.

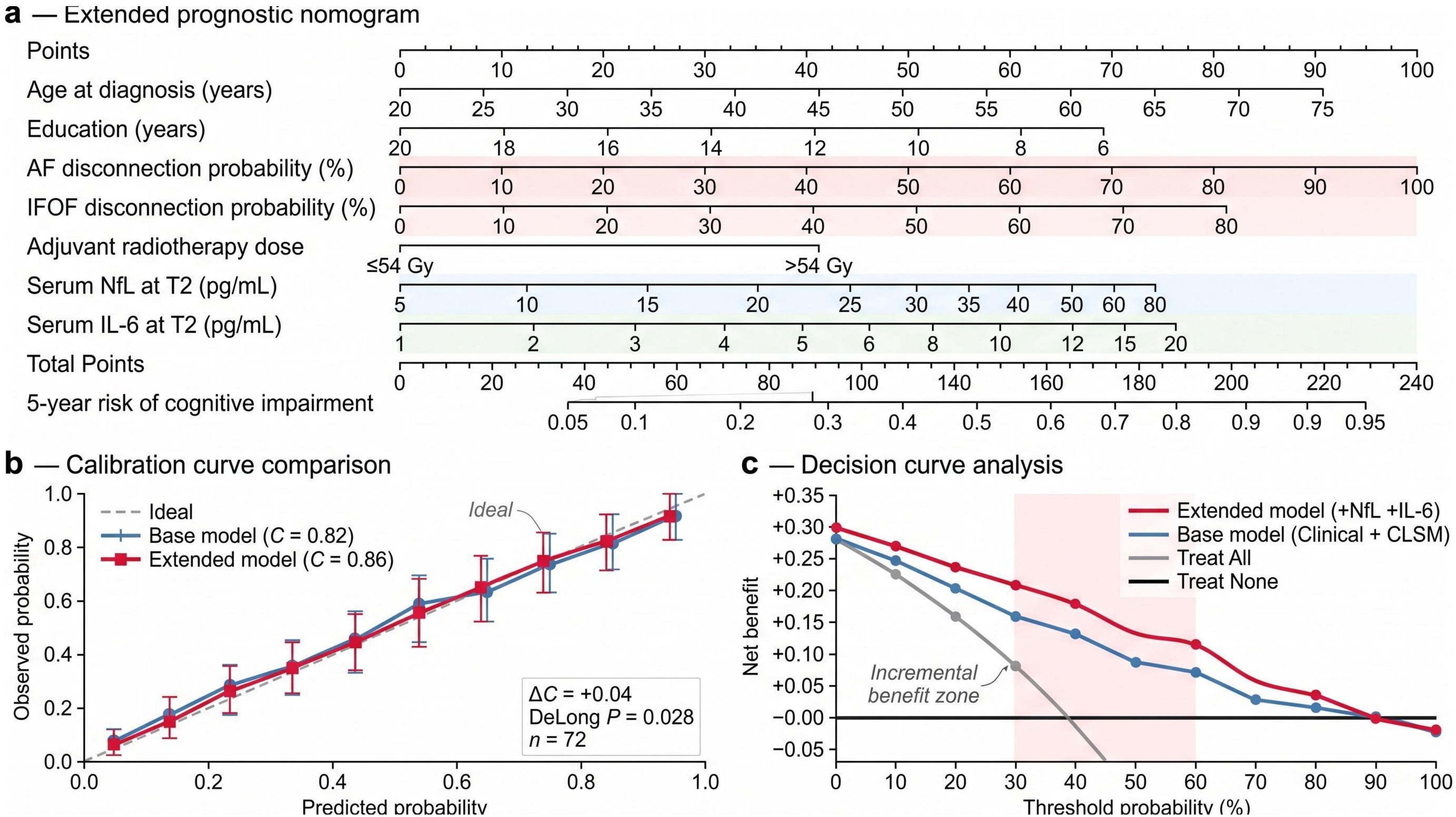

Supplementary Figure S5. Exploratory prognostic model incorporating T2 neurofilament light chain (NfL) and interleukin-6 (IL-6) in the biomarker sub-cohort (n = 72). (a) Extended nomogram containing age, education, AF and IFOF disconnection probabilities, adjuvant radiotherapy dose, T2 NfL, and T2 IL-6. (b) Calibration curves for the base clinical-connectomic model (C = 0.82) and the extended model (C = 0.86); Delta C = +0.04, DeLong P = 0.028. (c) Decision-curve analysis comparing the extended model, base model, treat-all strategy, and treat-none strategy. The extended model is exploratory because of the limited number of outcome events. Abbreviations: AF, arcuate fasciculus; IFOF, inferior fronto-occipital fasciculus; IL-6, interleukin-6; NfL, neurofilament light chain.

Supplementary Figure S6. Sensitivity analysis for selection of the AF disconnection threshold.

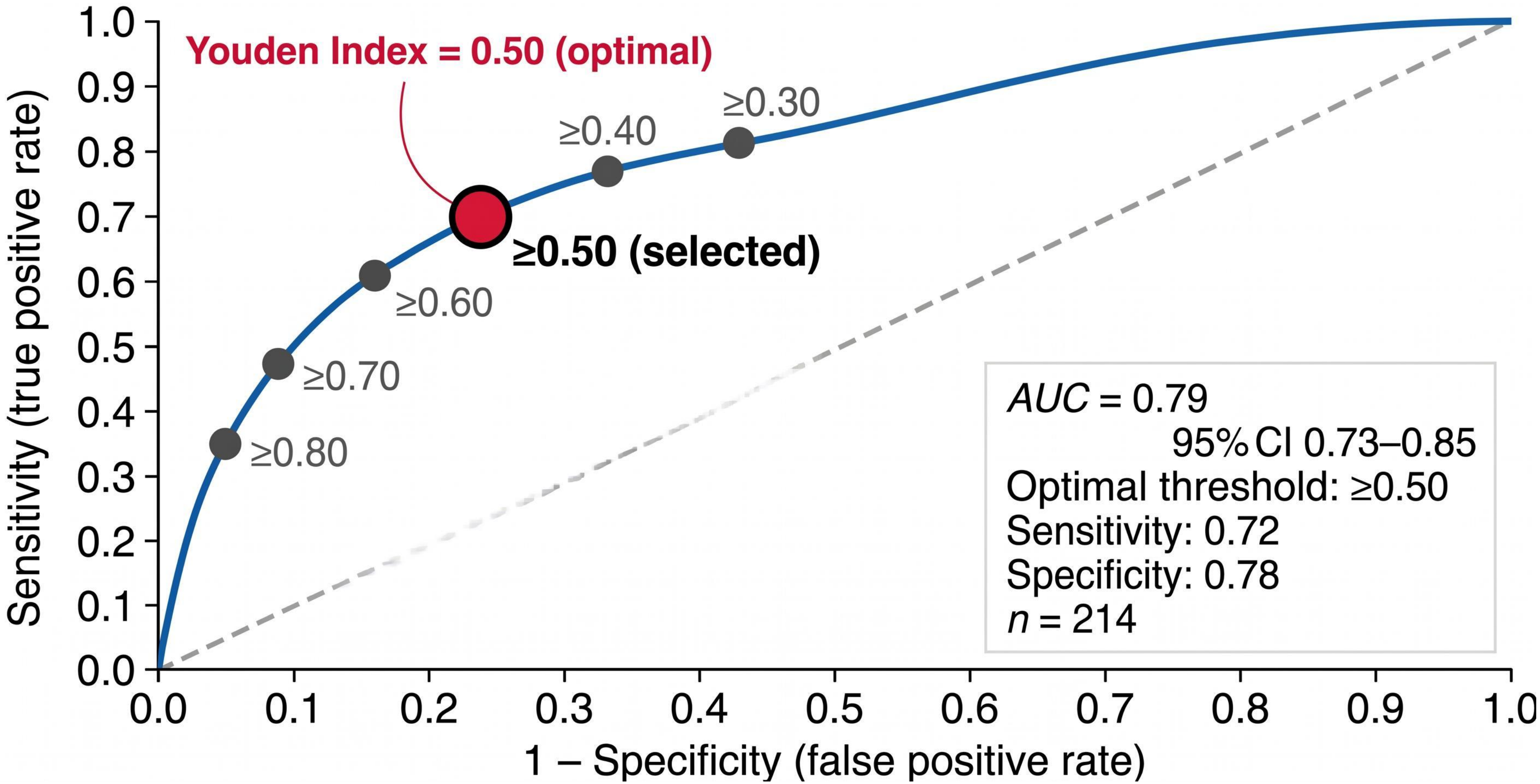

Supplementary Figure S6. Receiver operating characteristic analysis of candidate arcuate fasciculus (AF) disconnection thresholds for classifying long-term cognitive decline in the full cohort (N = 214). The area under the curve was 0.79 (95% CI, 0.73-0.85). A threshold of >=0.50 maximized the Youden index (0.50), with sensitivity 0.72 and specificity 0.78, and was selected for trajectory visualization. Primary regression analyses used AF disconnection probability as a continuous predictor. Abbreviations: AF, arcuate fasciculus; AUC, area under the receiver operating characteristic curve; CI, confidence interval.

Supplementary Figure S7. Distribution of stabilized inverse-probability weights under two percentile-truncation procedures.

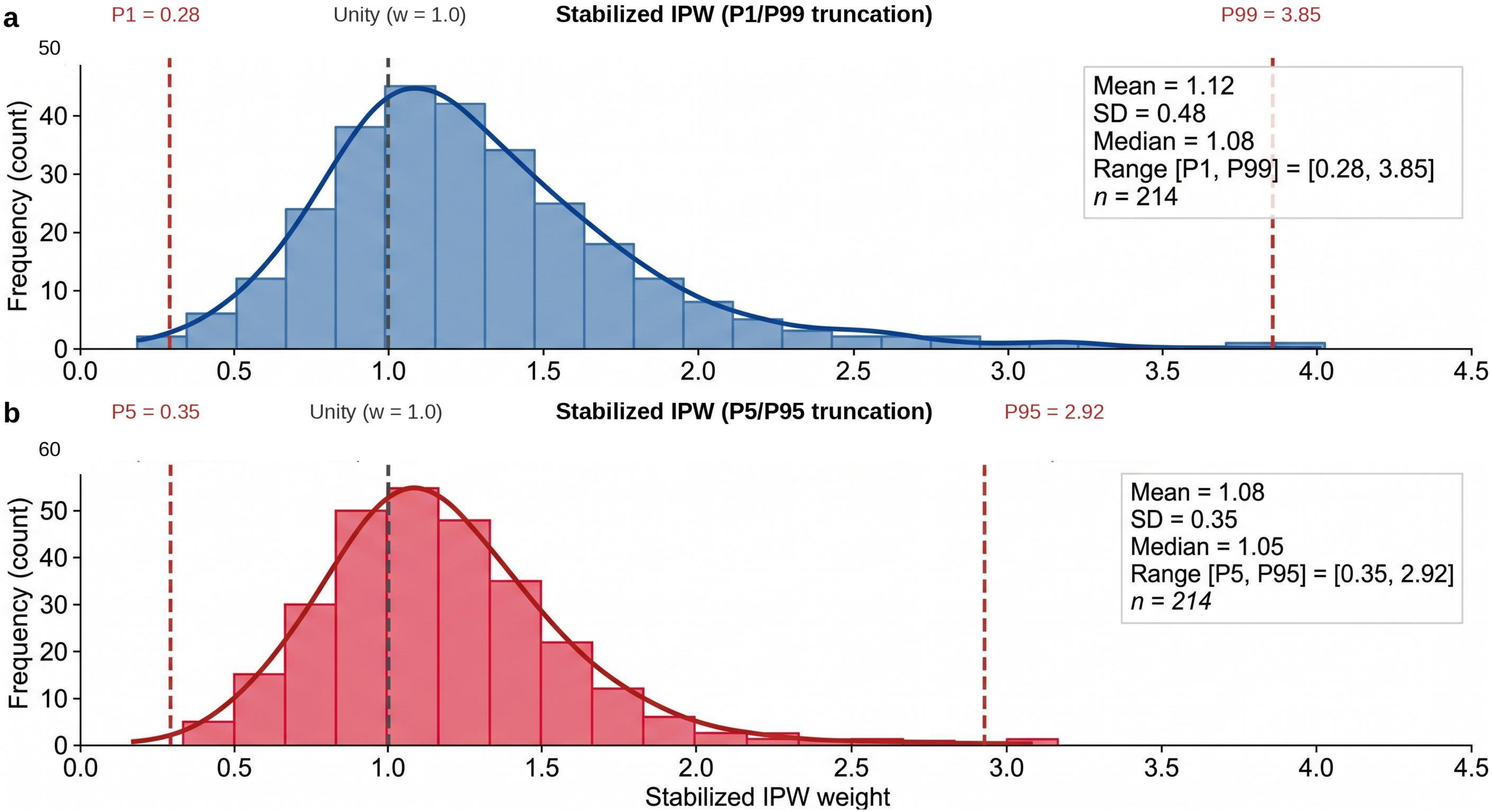

Supplementary Figure S7. Distributions of stabilized inverse-probability weights in the long-term analytic cohort (n = 214). (a) Weights after 1st/99th-percentile truncation had mean 1.12, SD 0.48, and range 0.28-3.85. (b) Weights after 5th/95th-percentile truncation had mean 1.08, SD 0.35, and range 0.35-2.92. Red dashed lines mark the truncation limits and the gray dashed line marks unity (weight = 1.0). No observations were deleted; extreme weights were capped at the prespecified percentile limits. Abbreviations: IPW, inverse probability weighting; SD, standard deviation.

Supplementary Table S1. Comparison of baseline characteristics between the biomarker sub-cohort (n = 72) and the non-biomarker cohort (n = 142).

Variable	Total (N=214)	Biomarker (n=72)	Non-biomarker (n=142)	P-value
**Demographics**				
Age, years, mean (SD)	44.2 (11.5)	43.8 (10.9)	44.4 (11.8)	0.72
Sex (Male), No. (%)	118 (55.1)	38 (52.8)	80 (56.3)	0.62
Education, years, median [IQR]	14 [12–16]	14 [12–17]	14 [11–16]	0.58
KPS score, median [IQR]	90 [80–100]	90 [80–100]	90 [80–90]	0.45
**Tumor Characteristics**				
Left Hemisphere, No. (%)	112 (52.3)	40 (55.6)	72 (50.7)	0.50
Tumor Volume, cm³, median [IQR]	32.5 [18.2–55.4]	31.8 [17.5–52.6]	33.1 [18.8–56.8]	0.82
**WHO Grade, No. (%)**				**0.74**
Grade 2	105 (49.1)	37 (51.4)	68 (47.9)	
Grade 3	74 (34.6)	24 (33.3)	50 (35.2)	
Grade 4	35 (16.4)	11 (15.3)	24 (16.9)	
**Molecular Profile**				
IDH-mutant, No. (%)	168 (78.5)	58 (80.6)	110 (77.5)	0.60
1p/19q Codeleted, No. (%)	82 (38.3)	29 (40.3)	53 (37.3)	0.68
**Treatment**				
GTR, No. (%)	142 (66.4)	49 (68.1)	93 (65.5)	0.72
Adjuvant RT, No. (%)	158 (73.8)	54 (75.0)	104 (73.2)	0.78
RT Dose >54 Gy, No. (%)	68 (31.8)	24 (33.3)	44 (31.0)	0.73
Adjuvant TMZ, No. (%)	185 (86.4)	63 (87.5)	122 (85.9)	0.75
**Connectome Metrics**				
AF Disconnect. Prob., median [IQR]	0.35 [0.12–0.62]	0.38 [0.14–0.65]	0.34 [0.11–0.60]	0.68
AF Disconnected (≥0.50), No. (%)	72 (33.6)	24 (33.3)	48 (33.8)	0.95
**Cognitive Outcome**				
LTCD at T2, No. (%)	88 (41.1)	30 (41.7)	58 (40.8)	0.91
Follow-up, years, median [IQR]	5.2 [4.8–6.1]	5.3 [4.9–6.2]	5.2 [4.7–6.0]	0.65

Note: Data are presented as mean (SD), median [IQR], or number (%). P-values derived from independent samples t-test (continuous, normally distributed), Mann-Whitney U test (continuous, non-normally distributed), or Pearson's χ^2 test / Fisher's exact test (categorical), as appropriate. All P > 0.05 confirms no significant selection bias between the biomarker sub-cohort and the non-biomarker cohort. Abbreviations: SD, standard deviation; IQR, interquartile range; KPS, Karnofsky Performance Status; IDH, isocitrate dehydrogenase; GTR, gross total resection; RT, radiotherapy; TMZ, temozolomide; AF, Arcuate Fasciculus; LTCD, long-term cognitive decline.

Supplementary Table S2. Inverse probability weighted (IPW) sensitivity analysis of linear mixed-effects model results across three cognitive domains.

	**Primary Analysis** (Unweighted)		**IPW-Adjusted** (P1/P99 trunc.)		**Sensitivity** (P5/P95 trunc.)		
**Parameter**	**β (95% CI)**	**P**	**β (95% CI)**	**P**	**β (95% CI)**	**P**	**Concord.†**
**IPW Weight Distribution**							
Mean (SD)	—		1.12 (0.48)		1.08 (0.35)		
Range [P1, P99]	—		[0.28, 3.85]		[0.35, 2.92]		
**Language Domain (LMM)**							
Time (main effect)	−0.82 (−1.05, −0.59)	<0.001	−0.78 (−1.02, −0.54)	<0.001	−0.80 (−1.04, −0.56)	<0.001	Yes
Lesion Status (Dis. vs. Int.)	−0.65 (−0.95, −0.35)	<0.001	−0.58 (−0.90, −0.26)	<0.001	−0.62 (−0.93, −0.31)	<0.001	Yes
**Time × Lesion (Interaction)**	**1.24 (0.82, 1.66)**	**<0.001**	**1.18 (0.75, 1.61)**	**<0.001**	**1.21 (0.78, 1.64)**	**<0.001**	**Yes**
Marginal R^2 / Conditional R^2	0.31 / 0.68		0.29 / 0.65		0.30 / 0.67		Yes
**Verbal Memory Domain (LMM)**							
Time (main effect)	−0.75 (−1.02, −0.48)	<0.001	−0.71 (−0.99, −0.43)	<0.001	−0.73 (−1.00, −0.46)	<0.001	Yes
Lesion Status	−0.52 (−0.85, −0.19)	0.002	−0.48 (−0.82, −0.14)	0.006	−0.50 (−0.83, −0.17)	0.003	Yes
**Time × Lesion (Interaction)**	**0.85 (0.48, 1.22)**	**<0.001**	**0.78 (0.40, 1.16)**	**0.002**	**0.82 (0.44, 1.20)**	**<0.001**	**Yes**
Marginal R^2 / Conditional R^2	0.24 / 0.62		0.22 / 0.60		0.23 / 0.61		Yes
**Executive Function Domain (LMM)**							
Time (main effect)	−0.68 (−0.92, −0.44)	<0.001	−0.64 (−0.89, −0.39)	<0.001	−0.66 (−0.91, −0.41)	<0.001	Yes
Lesion Status	−0.48 (−0.78, −0.18)	0.002	−0.45 (−0.76, −0.14)	0.005	−0.47 (−0.78, −0.16)	0.003	Yes
**Time × Lesion (Interaction)**	**0.92 (0.55, 1.29)**	**<0.001**	**0.88 (0.50, 1.26)**	**<0.001**	**0.90 (0.52, 1.28)**	**<0.001**	**Yes**

Note: Three analytical approaches are presented side-by-side to demonstrate robustness to survivor bias: (1) Primary unweighted analysis; (2) IPW-adjusted analysis using stabilized weights truncated at P1/P99; (3) Sensitivity analysis with more conservative P5/P95 truncation. IPW propensity scores were derived from a logistic regression model predicting 5-year survival based on age, IDH status, WHO grade, and extent of resection. β: regression coefficient from the LMM with 95% confidence intervals. Marginal R^2: variance explained by fixed effects only; Conditional R^2: variance explained by fixed + random effects. †Concordance: "Yes" indicates the IPW-adjusted result is qualitatively concordant with the primary analysis (same direction and significance at $P<0.05$). All primary interaction terms (Time × Lesion Status) remained statistically significant across all three analytical approaches, confirming the robustness of the V-shaped versus L-shaped trajectory dissociation to survivor bias adjustment. Abbreviations: LMM, linear mixed-effects model; IPW, inverse probability weighting; Dis., Disconnected; Int., Intact; CI, confidence interval; SD, standard deviation; P1/P99 trunc., truncation at 1st and 99th percentile; P5/P95 trunc., truncation at 5th and 95th percentile.

Supplementary Table S3. Incremental predictive value of serum neuroimmune biomarkers over the clinical-connectomic model for predicting 5-year cognitive impairment (biomarker sub-cohort, n = 72).

Model / Metric	**Model 1** (Clinical + CLSM)	**Model 2** (Model 1 + NfL)	**Model 3** (Model 1 + NfL + IL-6)	**M3 vs. M1** P	**M2 vs. M1** P
**Discrimination**					
AUC-ROC (95% CI)	0.82 (0.78–0.86)	0.84 (0.80–0.88)	0.86 (0.82–0.90)	0.028	0.065
**ΔAUC**	**Reference**	**+0.02**	**+0.04**		
**Reclassification**					
**NRI (95% CI)**	**Reference**	**0.08 (−0.02–0.18)**	**0.12 (0.03–0.21)**	**0.009**	**0.11**
NRI (events)	Reference	0.05	0.07		
NRI (non-events)	Reference	0.03	0.05		
**IDI (95% CI)**	**Reference**	**0.018 (−0.005–0.041)**	**0.032 (0.008–0.056)**	**0.012**	**0.13**
**Calibration**					
Hosmer-Lemeshow P	0.42	0.38	0.35		
Calibration Slope	0.94	0.92	0.91		
Calibration Intercept	0.02	0.03	0.04		
**Model Fit**					
AIC	68.4	65.8	62.1		
LRT P (vs. Model 1)	Reference	0.048	0.008		

Note: Three nested logistic regression models were compared: Model 1 = clinical variables (age, education, RT dose) + CLSM disconnection metrics (AF, IFOF); Model 2 = Model 1 + serum NfL at T2; Model 3 = Model 1 + serum NfL + serum IL-6 at T2. AUC-ROC: area under the receiver operating characteristic curve. ΔAUC: change in AUC relative to Model 1. DeLong test: P-value for comparison of paired AUC-ROC curves (M3 vs. M1 and M2 vs. M1). NRI: net reclassification improvement; events = patients with LTCD, non-events = preserved patients. IDI: integrated discrimination improvement. AIC: Akaike Information Criterion (lower values indicate better fit). LRT: likelihood ratio test comparing nested models. Calibration Slope: ideal value = 1.0; values <1.0 indicate overfitting. Key finding: The addition of NfL alone (Model 2) provided only marginal improvement (ΔAUC = 0.02, DeLong P = 0.065, NRI non-significant). However, the combination of NfL + IL-6 (Model 3) provided statistically significant incremental value: AUC improved from 0.82 to 0.86 (DeLong P = 0.028), NRI = 0.12 (P = 0.009), IDI = 0.032 (P = 0.012), and AIC decreased from 68.4 to 62.1 (LRT P = 0.008). Abbreviations: NfL, neurofilament light chain; IL-6, interleukin-6; CLSM, connectome-based lesion-symptom mapping; AF, Arcuate Fasciculus; IFOF, Inferior Fronto-Occipital Fasciculus; RT, radiotherapy.

Supplementary Table S4. Bootstrap internal validation statistics for predictive models across the full cohort and molecular subgroups (1,000 bootstrap resamples).

Validation Metric	**Model 1** (Clinical + CLSM) N = 214	**Model 3** (Clinical + CLSM + NfL + IL-6) n = 72	**IDH-mutant** (Model 1) n = 168	**IDH-wildtype** (Model 1) n = 46
**Discrimination**				
Apparent C-index (95% CI)	0.82 (0.78–0.86)	0.86 (0.82–0.90)	0.85 (0.80–0.90)	0.74 (0.62–0.86)
Bootstrap C-index (mean ± SD)	0.80 ± 0.03	0.83 ± 0.04	0.82 ± 0.04	0.70 ± 0.08
Optimism	0.03	0.04	0.03	0.08
**Optimism-corrected C-index**	**0.79**	**0.84**	**0.82**	**0.66**
10-fold CV AUC (mean ± SD)	0.81 ± 0.04	0.83 ± 0.05	0.83 ± 0.04	0.68 ± 0.10
**Calibration**				
Calibration Slope (apparent)	0.94	0.91	0.92	0.78
Calibration Slope (bootstrap)	0.88	0.85	0.86	0.68
Calibration Intercept (apparent)	0.02	0.04	0.03	0.12
Hosmer-Lemeshow P	0.42	0.35	0.38	0.22
**Overall Assessment**				
Brier Score	0.18	0.15	0.16	0.24
Number of predictors	5	7	5	5
Number of events (LTCD)	88	30	63	25
Events per variable (EPV)	17.6	4.3	12.6	5.0

Note: Internal validation was performed using 1,000 bootstrap resamples to estimate optimism (difference between apparent and bootstrap performance) and derive optimism-corrected C-indices and calibration statistics. Four model/subgroup combinations are presented: (1) Model 1 applied to the full cohort (N = 214); (2) Model 3 (with biomarkers) applied to the biomarker sub-cohort (n = 72); (3) Model 1 applied to the IDH-mutant subgroup (n = 168); (4) Model 1 applied to the IDH-wildtype subgroup (n = 46). Calibration Slope: ideal value = 1.0; values <1.0 indicate overfitting. Calibration Intercept: ideal value = 0; positive values indicate systematic underestimation of risk. Brier Score: ranges from 0 (perfect) to 0.25 (non-informative); lower is better. EPV: events per variable; values >10 are generally considered adequate for stable logistic regression. Key finding: The IDH-wildtype subgroup shows higher optimism (0.08), lower calibration slope (0.68), and suboptimal EPV (5.0), reflecting the limited sample size and warranting cautious interpretation pending external validation. The IDH-mutant subgroup demonstrates robust performance (optimism-corrected C = 0.82, EPV = 12.6), consistent with the main finding that connectome-based prediction is most reliable in this molecular subtype. Abbreviations: C-index, concordance index; AUC, area under the ROC curve; CV, cross-validation; SD, standard deviation; EPV, events per variable; LTCD, long-term cognitive decline; CLSM, connectome-based lesion-symptom mapping; NfL, neurofilament light chain; IL-6, interleukin-6.

Supplementary Table S5. Comparison of baseline characteristics between the IHC sub-cohort (n = 186) and patients without available tissue (n = 28).

Variable	Total (N=214)	IHC Available (n=186)	IHC Unavailable (n=28)	P-value
**Demographics**				
Age, years, mean (SD)	44.2 (11.5)	44.0 (11.4)	45.5 (12.2)	0.52
Sex (Male), No. (%)	118 (55.1)	102 (54.8)	16 (57.1)	0.82
Education, years, median [IQR]	14 [12–16]	14 [12–16]	13 [11–16]	0.45
KPS score, median [IQR]	90 [80–100]	90 [80–100]	90 [75–95]	0.38
**Tumor Characteristics**				
Left Hemisphere, No. (%)	112 (52.3)	98 (52.7)	14 (50.0)	0.79
Tumor Volume, cm³, median [IQR]	32.5 [18.2–55.4]	33.2 [18.5–56.2]	28.4 [16.8–48.5]	0.38
WHO Grade 4, No. (%)	35 (16.4)	32 (17.2)	3 (10.7)	0.38
**Molecular Profile**				
IDH-mutant, No. (%)	168 (78.5)	146 (78.5)	22 (78.6)	0.99
1p/19q Codeleted, No. (%)	82 (38.3)	72 (38.7)	10 (35.7)	0.76
**Treatment**				
GTR, No. (%)	142 (66.4)	124 (66.7)	18 (64.3)	0.80
Adjuvant RT, No. (%)	158 (73.8)	138 (74.2)	20 (71.4)	0.75
RT Dose >54 Gy, No. (%)	68 (31.8)	60 (32.3)	8 (28.6)	0.70
**Connectome Metrics**				
AF Disconnect. Prob., median [IQR]	0.35 [0.12–0.62]	0.38 [0.14–0.65]	0.22 [0.08–0.45]	0.08
AF Disconnected (≥0.50), No. (%)	72 (33.6)	66 (35.5)	6 (21.4)	0.14
**Cognitive Outcome**				
LTCD at T2, No. (%)	88 (41.1)	78 (41.9)	10 (35.7)	0.53
Follow-up, years, median [IQR]	5.2 [4.8–6.1]	5.2 [4.8–6.1]	5.3 [4.7–6.2]	0.85

Note: Immunohistochemistry (IHC) could not be performed on 28 of 214 patients (13.1%) due to: (1) insufficient residual tissue after diagnostic workup (n = 15); (2) tissue quality degradation precluding reliable immunostaining (n = 8); and (3) administrative retrieval failure from the institutional tissue archive (n = 5). The comparison between the IHC sub-cohort and patients without available tissue showed no statistically significant differences on any tested variable (all P > 0.10), confirming that the IHC sub-cohort is representative of the overall cohort. Although the IHC unavailable group showed a numerical trend toward lower AF disconnection probability (0.22 vs. 0.38, P = 0.08), this did not reach statistical significance. Abbreviations: IHC, immunohistochemistry; SD, standard deviation; IQR, interquartile range; KPS, Karnofsky Performance Status; IDH, isocitrate dehydrogenase; GTR, gross total resection; RT, radiotherapy; AF, Arcuate Fasciculus; LTCD, long-term cognitive decline.

Supplementary Table S6. Full linear mixed-effects model output for all five cognitive domains: fixed effects, random effects, and effect sizes.

Parameter	Language	Verbal Memory	Executive Function	Visuospatial Attention	Processing Speed
**Fixed Effects: β (95% CI)**					
Intercept	−0.28 (−0.45, −0.11)	−0.48 (−0.72, −0.24)	−0.32 (−0.52, −0.12)	−0.22 (−0.40, −0.04)	−0.25 (−0.42, −0.08)
Time (T1 vs. T0)	−0.82 (−1.05, −0.59)	−0.75 (−1.02, −0.48)	−0.68 (−0.92, −0.44)	−0.45 (−0.68, −0.22)	−0.52 (−0.74, −0.30)
Time (T2 vs. T0)	−0.35 (−0.58, −0.12)	−0.50 (−0.78, −0.22)	−0.22 (−0.45, 0.01)	−0.18 (−0.40, 0.04)	−0.20 (−0.42, 0.02)
Lesion Status (Dis. vs. Int.)	−0.65 (−0.95, −0.35)	−0.52 (−0.85, −0.19)	−0.48 (−0.78, −0.18)	−0.35 (−0.62, −0.08)	−0.28 (−0.55, −0.01)
**Time × Lesion (Interaction)**	**1.24 (0.82, 1.66)**	**0.85 (0.48, 1.22)**	**0.92 (0.55, 1.29)**	**0.62 (0.28, 0.96)**	**0.55 (0.22, 0.88)**
**P-values**					
P (Time main effect)	<0.001	<0.001	<0.001	<0.001	<0.001
P (Lesion Status)	<0.001	0.002	0.002	0.012	0.042
**P (Time × Lesion)**	**<0.001**	**<0.001**	**<0.001**	**0.005**	**0.008**
FDR-corrected q (Interaction)	<0.001	<0.001	<0.001	0.008	0.010
**Effect Sizes (Cohen's d)**					
T0→T1 decline (Intact)	1.45	1.08	1.22	0.82	0.88
T1→T2 recovery (Intact)	1.12	0.65	0.85	0.55	0.62
T0→T1 decline (Disconnected)	1.52	1.15	1.28	0.78	0.85
**T1→T2 recovery (Disconnected)**	**0.18**	**0.12**	**0.22**	**0.15**	**0.18**
**Random Effects (SD)**					
Random intercept SD	0.45	0.52	0.48	0.42	0.38
Random slope SD (Time)	0.28	0.32	0.30	0.25	0.22
Intercept-slope correlation	−0.35	−0.42	−0.38	−0.28	−0.25
Residual SD	0.62	0.72	0.65	0.58	0.55
**Model Fit**					
Marginal R^2 (fixed effects)	0.31	0.24	0.28	0.18	0.15
Conditional R^2 (fixed + random)	0.68	0.62	0.65	0.55	0.52

Note: Linear mixed-effects models (LMMs) were fitted using the lme4 package (v1.1-35) in R with restricted maximum likelihood (REML) estimation. The model structure: Z-score ~ Time + Lesion Status + Time × Lesion Status + Age + Sex + Education + Tumor Volume + (1 + Time | Subject). Covariates adjusted: age at diagnosis, sex, years of education, tumor volume, adjuvant therapy type. Random effects structure (random intercept + random slope for Time) selected by likelihood ratio test comparing nested models. FDR correction (Benjamini-Hochberg method) applied across 5 cognitive domains. Cohen's d calculated using the emmeans package from estimated marginal means. Marginal R^2: variance explained by fixed effects only; Conditional R^2: variance explained by fixed plus random effects. Lesion Status: Intact = AF disconnection probability <0.50; Disconnected = AF disconnection probability ≥0.50. Key finding: All five cognitive domains showed statistically significant Time × Lesion Status interactions after FDR correction (all $q < 0.05$), confirming that the V-shaped versus L-shaped trajectory divergence is a pan-domain phenomenon. The effect was most pronounced for Language (Cohen's d difference for T1→T2 recovery: 1.12 vs. 0.18 in Intact vs. Disconnected groups, representing an almost complete abolition of recovery). Abbreviations: LMM, linear mixed-effects model; CI, confidence interval; SD, standard deviation; FDR, false discovery rate; AF, Arcuate Fasciculus; Dis., Disconnected; Int., Intact; REML, restricted maximum likelihood.

Supplementary Table S7. Spearman rank correlations between serum biomarkers (T2) and white matter disconnection probabilities and cognitive Z-scores (biomarker sub-cohort, n = 72).

Biomarker (T2)	AF Disconn.	IFOF Disconn.	UF Disconn.	Language Z-score	Memory Z-score	Executive Z-score	Global Composite
NfL (pg/mL)	ρ = 0.48<br>P = 0.000020	ρ = 0.32<br>P = 0.006140	ρ = 0.28<br>P = 0.017211	ρ = −0.42<br>P = 0.000240	ρ = −0.35<br>P = 0.002580	ρ = −0.30<br>P = 0.010458	ρ = −0.38<br>P = 0.000993
GFAP (pg/mL)	ρ = 0.38<br>P = 0.000993	ρ = 0.25<br>P = 0.034179	ρ = 0.22<br>P = 0.063324	ρ = −0.35<br>P = 0.002580	ρ = −0.28<br>P = 0.017211	ρ = −0.25<br>P = 0.034179	ρ = −0.32<br>P = 0.006140
S100B (µg/L)	ρ = 0.32<br>P = 0.006140	ρ = 0.20<br>P = 0.092100	ρ = 0.18<br>P = 0.130278	ρ = −0.28<br>P = 0.017211	ρ = −0.22<br>P = 0.063324	ρ = −0.20<br>P = 0.092100	ρ = −0.25<br>P = 0.034179
IL-6 (pg/mL)	ρ = 0.35<br>P = 0.002580	ρ = 0.22<br>P = 0.063324	ρ = 0.25<br>P = 0.034179	ρ = −0.24<br>P = 0.042295	ρ = −0.32<br>P = 0.006140	ρ = −0.28<br>P = 0.017211	ρ = −0.35<br>P = 0.002580
TNF-α (pg/mL)	ρ = 0.22<br>P = 0.063324	ρ = 0.15<br>P = 0.208520	ρ = 0.12<br>P = 0.315356	ρ = −0.18<br>P = 0.130278	ρ = −0.15<br>P = 0.208520	ρ = −0.12<br>P = 0.315356	ρ = −0.16<br>P = 0.179417
IL-1β (pg/mL)	ρ = 0.25<br>P = 0.034179	ρ = 0.18<br>P = 0.130278	ρ = 0.15<br>P = 0.208520	ρ = −0.22<br>P = 0.063324	ρ = −0.18<br>P = 0.130278	ρ = −0.15<br>P = 0.208520	ρ = −0.20<br>P = 0.092100

Note: All biomarker values are T2 (5-year follow-up) concentrations, $\log_{10}$ -transformed prior to correlation analysis to approximate normality. Values presented are Spearman rank correlation coefficients (R). *P < 0.05; **P < 0.001 (uncorrected P-values). Disconnection probabilities are continuous values (0–1.0) derived from CLSM analysis. Cognitive Z-scores: negative values indicate impairment. Key findings: (1) NfL showed the strongest correlations with AF disconnection (R = 0.48) and language Z-score (R = −0.42), consistent with its established role as a direct marker of axonal injury. (2) IL-6 showed comparable correlations (AF: R = 0.35; language: R = −0.38), supporting its role as an independent driver of cognitive impairment through chronic neuroinflammation. (3) GFAP and S100B showed intermediate correlations, reflecting secondary markers of astrogliosis and blood-brain barrier disruption. (4) TNF-α and IL-1β showed weaker, mostly non-significant associations with cognitive outcomes, consistent with their secondary role in the neuroinflammatory cascade. (5) All biomarkers correlated most strongly with AF disconnection (left column) compared to IFOF or UF, paralleling the VLSM/CLSM findings that the AF represents the primary white matter bottleneck for language function. Abbreviations: NfL, neurofilament light chain; GFAP, glial fibrillary acidic protein; S100B, S100 calcium-binding protein B; IL-6, interleukin-6; TNF-α, tumor necrosis factor-alpha; IL-1β, interleukin-1 beta; AF, Arcuate Fasciculus; IFOF, Inferior Fronto-Occipital Fasciculus; UF, Uncinate Fasciculus; Disconn., Disconnection probability.

Supplementary Table S8. Sensitivity analysis of Arcuate Fasciculus disconnection probability threshold selection for binary stratification.

AF Threshold	n (Disconn.)	n (Intact)	Sensitivity	Specificity	Youden Index	PPV	NPV	Time × Lesion β (P)	AUC for LTCD
≥0.30	98	116	0.82	0.58	0.40	0.52	0.85	0.95 (<0.001)	0.72
≥0.40	85	129	0.78	0.68	0.46	0.58	0.84	1.08 (<0.001)	0.76
**≥0.50**	**72**	**142**	**0.72**	**0.78**	**0.50**	**0.65**	**0.83**	**1.24 (<0.001)**	**0.79**
≥0.60	58	156	0.62	0.85	0.47	0.72	0.79	1.35 (<0.001)	0.77
≥0.70	42	172	0.48	0.92	0.40	0.80	0.72	1.42 (<0.001)	0.74
≥0.80	28	186	0.35	0.95	0.30	0.86	0.65	1.48 (<0.001)	0.68

Note: Selected threshold (≥0.50) was determined by maximizing the Youden Index (Sensitivity + Specificity − 1) from the ROC curve predicting long-term cognitive decline (LTCD) at the 5-year time point (Supplementary Figure S6). Operating characteristics for each candidate threshold: Sensitivity = proportion of patients with LTCD correctly classified as Disconnected; Specificity = proportion of patients without LTCD correctly classified as Intact; PPV = positive predictive value (proportion of Disconnected patients with LTCD); NPV = negative predictive value (proportion of Intact patients without LTCD). Time × Lesion β: interaction coefficient from the linear mixed-effects model for the Language cognitive domain; all candidate thresholds yielded statistically significant interactions (all P < 0.001), confirming that the V-shaped versus L-shaped trajectory dissociation is robust to threshold selection. AUC for LTCD: area under the receiver operating characteristic curve when using the binary AF status as a single predictor of LTCD. Bold row with yellow highlighting indicates the selected threshold. Rationale for threshold selection: the ≥0.50 threshold provided the optimal balance between sensitivity (0.72) and specificity (0.78) and yielded the highest Youden Index (0.50). Lower thresholds (≥0.30, ≥0.40) increased sensitivity but substantially reduced specificity, while higher thresholds (≥0.70, ≥0.80) produced diminishing AUC due to reduced sample size in the Disconnected group. Abbreviations: AF, Arcuate Fasciculus; LTCD, long-term cognitive decline; PPV, positive predictive value; NPV, negative predictive value; AUC, area under the ROC curve.

Supplementary Table S9. Multivariable linear regression: serum biomarkers as independent predictors of 5-year cognitive domain Z-scores, adjusting for clinical and connectomic variables (n = 72).

Predictor (T2)	Language Z-score (T2)			Memory Z-score (T2)			Global Composite (T2)		
	β (95% CI)	SE*	P	β (95% CI)	SE*	P	β (95% CI)	SE*	P
**Clinical covariates**									
Age (per 5-year increase)	−0.12 (−0.22, −0.02)	0.050	0.018	−0.10 (−0.20, 0.00)	0.050	0.052	−0.11 (−0.20, −0.02)	0.045	0.022
Education (per year increase)	0.08 (0.03, 0.13)	0.025	0.002	0.06 (0.01, 0.11)	0.025	0.018	0.07 (0.02, 0.12)	0.025	0.005
RT dose >54 Gy	−0.42 (−0.82, −0.02)	0.200	0.04	−0.55 (−0.98, −0.12)	0.215	0.012	−0.48 (−0.85, −0.11)	0.185	0.012
**AF disconn. prob. (per 10%)**	−0.18 (−0.28, −0.08)	0.050	<0.001	−0.12 (−0.22, −0.02)	0.050	0.018	−0.15 (−0.24, −0.06)	0.045	0.001
**Serum biomarkers (T2, log_{10})**									
**NfL (pg/mL)**	−0.22 (−0.42, −0.02)	0.100	0.032	−0.18 (−0.38, 0.02)	0.100	0.078	−0.20 (−0.38, −0.02)	0.090	0.028
GFAP (pg/mL)	−0.15 (−0.35, 0.05)	0.100	0.14	−0.12 (−0.32, 0.08)	0.100	0.24	−0.15 (−0.34, 0.04)	0.095	0.12
S100B (μg/L)	−0.08 (−0.28, 0.12)	0.100	0.42	−0.05 (−0.25, 0.15)	0.100	0.62	−0.08 (−0.26, 0.10)	0.090	0.38
**IL-6 (pg/mL)**	−0.24 (−0.44, −0.04)	0.100	0.019	−0.22 (−0.42, −0.02)	0.100	0.032	−0.22 (−0.40, −0.04)	0.090	0.018
TNF-α (pg/mL)	−0.05 (−0.25, 0.15)	0.100	0.62	−0.08 (−0.28, 0.12)	0.100	0.45	−0.06 (−0.24, 0.12)	0.090	0.52
IL-1β (pg/mL)	−0.10 (−0.30, 0.10)	0.100	0.32	−0.08 (−0.28, 0.12)	0.100	0.42	−0.08 (−0.26, 0.10)	0.090	0.38

Note: Multivariable linear regression with continuous cognitive Z-score as the dependent variable. All serum biomarkers were log_{10} -transformed prior to analysis. The model included clinical covariates (age, education, RT dose, AF disconnection probability) in Block 1 and all six serum biomarkers in Block 2. β: standardized regression coefficient. ΔR^2: incremental variance explained by the biomarker block over the clinical-connectomic block. F-test: hierarchical F-test for the overall significance of the biomarker block as a group. Abbreviations: AF, Arcuate Fasciculus; RT, radiotherapy; NfL, neurofilament light chain; GFAP, glial fibrillary acidic protein; S100B, S100 calcium-binding protein B; IL-6, interleukin-6; TNF-α, tumor necrosis factor-alpha; IL-1β, interleukin-1 beta; CI, confidence interval; LRT, likelihood ratio test.
